# Chemical Relationship among Genetically Authenticated Medicinal Species of Genus Angelica

**DOI:** 10.3390/plants13091252

**Published:** 2024-04-30

**Authors:** Jung-Hoon Kim, Eui-Jeong Doh, Han-Young Kim, Guemsan Lee

**Affiliations:** 1 Division of Pharmacology, School of Korean Medicine, Pusan National University, Yangsan 50612, Republic of Korea; kmsct@pusan.ac.kr; 2 Research Center of Traditional Korean Medicine, Wonkwang University, Iksan 54538, Republic of Korea; bluemoon-lion@hanmail.net; 3 School of Korean Medicine, Pusan National University, Yangsan 50612, Republic of Korea; whatsnikers00@pusan.ac.kr; 4 Department of Herbology, College of Korean Medicine, Wonkwang University, Iksan 54538, Republic of Korea

**Keywords:** medicinal *Angelica* species, DNA barcoding, high-performance liquid chromatography, quantitative analysis, chemotaxonomic classification

## Abstract

The genus *Angelica* comprises various species utilized for diverse medicinal purposes, with differences attributed to the varying levels or types of inherent chemical components in each species. This study employed DNA barcode analysis and HPLC analysis to genetically authenticate and chemically classify eight medicinal *Angelica* species (*n* = 106) as well as two non-medicinal species (*n* = 14) that have been misused. Nucleotide sequence analysis of the nuclear internal transcribed spacer (ITS) region revealed differences ranging from 11 to 117 bp, while *psbA-trnH* showed variances of 3 to 95 bp, respectively. Phylogenetic analysis grouped all samples except *Angelica sinensis* into the same cluster, with some counterfeits forming separate clusters. Verification using the NCBI database confirmed the feasibility of species identification. For chemical identification, a robust quantitative HPLC analysis method was developed for 46 marker compounds. Subsequently, two *A. reflexa*-specific and seven *A. biserrata*-specific marker compounds were identified, alongside non-specific markers. Moreover, chemometric clustering analysis reflecting differences in chemical content between species revealed that most samples formed distinct clusters according to the plant species. However, some samples formed mixed clusters containing different species. These findings offer crucial insights for the standardization and quality control of medicinal *Angelica* species.

## 1. Introduction

The plants of the genus *Angelica* (family Apiaceae), along with their botanical synonyms, have been used as herbal medicines for various medicinal purposes, i.e., the roots of *Angelica gigas* Nakai (Korean herbal name: Dang-gwi), the roots of *A. acutiloba* (Siebold & Zucc.) Kitag. (Korean herbal name: Il-Dang-gwi), the roots of *A. sinensis* (Oliv.) Diels (Korean herbal name: Chinese Dang-gwi), the roots of *A. decursiva* Franch. & Sav. (=*Peucedanum decursivum* Maxim.) (Korean herbal name: Jeon-ho), the roots of *A. dahurica* (Hoffm.) Benth. & Hook.f. ex Franch. & Sav. (Korean herbal name: Baek-ji), the rhizomes and roots of *Conioselinum tenuissimum* (Nakai) Pimenov & Kljuykov [=*A. tenuissima* Nakai =*Ligusticum tenuissimum* (Nakai) Kitag.] (Korean herbal name: Go-bon), the roots of *A. biserrata* (R.H.Shan & Yuan) C.Q.Yuan & R.H.Shan [=*A. pubescens* f. *biserrata* R.H.Shan & C.Q.Yuan] (Korean herbal name: Chinese Dok-hwal), and the roots of *A. reflexa* B.Y.Lee (Korean herbal name: Gang-hwal) [1,2,3,4,5]. According to traditional theory, these *Angelica* herbal medicines possess distinctive therapeutic efficacy, addressing various ailments: Dang-gwi for tonifying blood circulation; Gang-hwal, Baek-ij, and Go-bon for dispersing wind-cold; Dok-hwal for dispelling wind dampness and alleviating impediment pain; and Jeon-ho for clearing and resolving heat phlegm [6]. These therapeutic variations among *Angelica* species may be attributed to differences in their innate chemical components, which are strongly influenced by interspecies differences [7].

Previous research endeavors have sought to categorize *Angelica* species based on their chemical, genetic, or morphological differences. Chromatographic techniques such as TLC, HPLC, and LC/MS have been employed to discern *A. sinensis*, *A. pubescens* f. *biserrata*, *A. dahurica,* and other related Umbelliferae plants by analyzing coumarins, phthalides, phenolics, and polyacetylene [8]. Additionally, seven *Angelica* species (*A. gigas*, *A. acutiloba*, *A. tenuissima*, *A. dahurica*, *A. koreana*, *A. polymorpha,* and *A. decusriva*) were authenticated through quantitative analysis of coumarins and micro-morphologies [9]. Furthermore, HPLC was utilized to differentiate *A. sinensis*, *A. acutiloba*, *A. acutiloba* var. *sugiyamae,* and other related Umbelliferae herbs based on their chromatographic fingerprints [10]. Lastly, quantitative analysis of coumarins and phenolics using HPLC enabled the chemical differentiation of three *Angelica* species of Dang-gwi (*A. gigas*, *A. acutiloba,* and *A. sinensis*) [11].

In this study, genetic analysis was employed as a tool to explore the phylogenetic relationships among various *Angelica* species as well as related plants within the Apiaceae family. These species were genetically classified using a combination of nrDNA internal transcribed spacer (ITS) and external transcribed spacer sequences, cpDNA sequences (*rpsl6* intron, *rpsl6-tmK*, *rpl32-trnL,* and *trnL-trnT*), and macro- and micro-morphological characteristics [12]. DNA barcoding regions, which included three chloroplast regions (*rbcL*, *matK,* and *trnH-psbA*) and the nuclear ITS region, were utilized to determine phylogenetic relationships among *A. sinensis*, *A. biserrata* and *A. dahurica* [13]. Chloroplast genome sequences were used to establish the phylogenetic relationships of 33 *Angelica* species and 31 other Apioideae species [14]. Another study reported the use of 5S-rRNA spacer domains and chemical components (ferulic acid and Z-ligustilide) as genetic and chemical markers, respectively, to compare species differences among *A. gigas*, *A. sinensis,* and *A. acutiloba* [15]. However, there were limitations in the study, as the samples of *Angelica* species used in the chemical analysis were not guaranteed by their exact botanical species, and the chemical relationships among the *Angelica* samples in the genetic analyses were not confirmed.

DNA authentication combined with chemical quantification has emerged as a powerful tool for determining chemotaxonomic relationships among botanically relevant species used for herbal medicines aided by chemometric analysis. The nuclear gene ITS and the chloroplast gene *trn*H-*psb*A were utilized to identify three species of the *Glycyrrhiza* genus, namely *Glycyrrhiza uralensis* Fisch., *G. inflata* Bat., and *G. glabra* L., supported by quantitative HPLC analysis of four marker compounds [16]. Three Arnebiae species, including *Arnebia decumbens* (Vent.) Coss. et Kralik, *A. euchroma* (Royle) Johnst, and *A. guttata,* were authenticated using DNA barcodes of ITS2 and quantitative analysis of seven naphthoquinones [17]. ITS, ITS2, and *psb*A-*trn*H were also employed to distinguish nine species of Fritillariae Bulbus such as *Fritillaria cirrhosa* D. Don, *F. delavayi* Franch, *F. przezvalskii* Maxim., *F. taipaiensis* P. Y. Li, *F. unibracteata* Hsiao et K. C. Hsia, *F. walujewii* Regel, *F. ussuriensis* Maxim., *F. thunbergii*, *F. pallidiflora* Schrenk, and *F. hupehensis* Hsiao, which was supported by HPLC/ELSD analysis of four alkaloids [18]. Our research team established a DNA authentication-hyphenated chemical profiling method and performed the classification of several herbal species, specifically the *Peucedanum*, *Amomum,* and *Atractylodes* genera [19,20,21].

As mentioned above, herbal medicines from *Angelica* species have been utilized for their medicinal purposes in Korean traditional medicine. However, there is controversy in defining the original species of Gang-hwal, of which the botanical origin is *Ostericum koreanum* Maximowicz in Korean pharmacopeia [1]. Therefore, in this study, to find the possible quantitative explanations for the differences in genus *Angelica*-oriented herbal medicines, we genetically identified eight species of medicinal *Angelica* genus and two non-medicinal *Angelica* species, namely, *A. polymorpha* Maxim. (Korean name: Gunggungi) and *Ostericum grossiserratum* (Maxim.) Kitag. (=*O. koreanum* (Maxim.) Kitag., *A. grosseserrata* Maxim., and *A. koreana* Maxim.) (Korean name: Singamchae). Those two non-medicinal species were examined to define the original species of Gang-hwal. We used the ITS and *psbA-trnH* regions for genetic identification. Furthermore, we chemically distinguished these species for chemotaxonomic classification using quantitative HPLC analysis of forty-six marker compounds. Subsequently, we investigated the chemical relationships among medicinal *Angelica* species as well as non-medicinal species and thereby found feasible alternatives to medicinal species.

## 2. Results

### 2.1. DNA Barcode Analysis

To identify the species among 120 samples derived from 8 medicinal species and 2 non-medicinal species of *Angelica* genus, including 13 samples of *A. acutiloba*, 10 samples of *A. biserrata*, 15 samples of *A. dahurica*, 13 samples of *A. decursiva*, 11 samples of *A. gigas*, 24 samples of *A. reflexa*, 12 samples of *A. sinensis*, 8 samples of *C. tenuissimum*, 6 samples of *A. polymorpha,* and 8 samples of *O. grossiserratum*, the nucleotide sequences of the ITS and *psbA-trnH* regions were analyzed. In the ITS region, approximately 688–694 bases of amplified product sequences were examined for each species. No intraspecies variation was observed in either the ITS or *psbA-trnH* nucleotide sequences. The analysis revealed nucleotide sequence differences ranging from 11 bp to 117 bp depending on the species, with an efficient classification of 10 species (sequence identity matrix range 0.832–0.978, Table S1). For the *psbA-trnH* region, approximately 307–350 bases of amplified product sequences were analyzed for each species. This region exhibited a 3–95 bp nucleotide sequence difference depending on the species (sequence identity matrix range 0.990–0.738, Table S2). The results of species discrimination in both the ITS and *psbA-trnH* regions were found to be consistent. The base sequence of the analyzed sample was confirmed for species identification through dual verification using both standard sample data and the NCBI database (Table 1).

### 2.2. Phylogenetic Analysis

**Figure 1:**
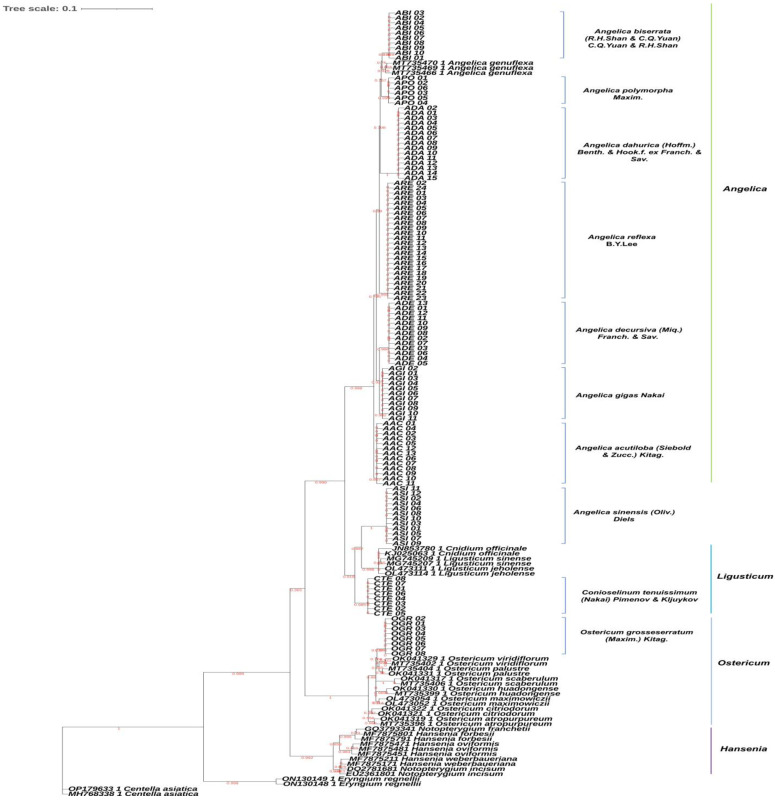
Phylogenetic tree representing the relationships among samples from various species based on the combination nucleotide sequences of internal transcribed spacer (ITS) and *psbA-trnH* region. *Angelica acutiloba* (AAC), *A. biserrata* (ABI), *A. dahurica* (ADA), *A. decursiva* (ADE), *Angelica gigas* (AGI), *A. polymorpha* (APO), *A. reflexa* (ARE), *A. sinensis* (ASI), *Conioselinum tenuissimum* (CTE), and *Ostericum grossiserratum* (OGR).

In the PhyML + SMS (Maximum likelihood-based inference of phylogenetic trees with Smart Model Selection) tree constructed based on concatenated nucleotide sequences of the ITS and *psbA-trnH* regions (Figure 1), the phylogenetic tree displayed clear separation by species, thereby supporting the accuracy of the identification results based on the two DNA barcode regions. All samples derived from the genus *Angelica* were clustered together, except for ASI (*A. sinense*). ASI was closely grouped with *L. jeholense* and *L. tenuissium* (=CTE), distinct from other genus *Angelica* samples. Additionally, one of the non-herbal species, *O. grossiserratum* (OGR), was closely clustered with other *Ostericum* species, forming a distinct cluster.

### 2.3. Quantitative Comparison of the Marker Compounds in Different *Angelica* Species

**Figure 2:**
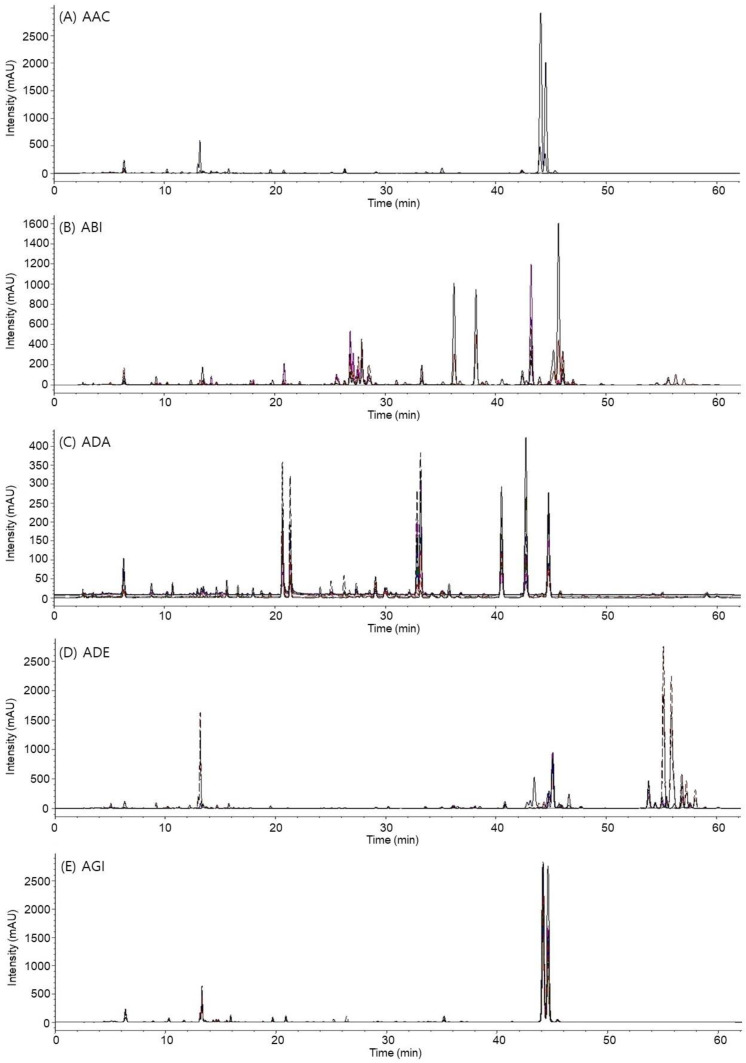

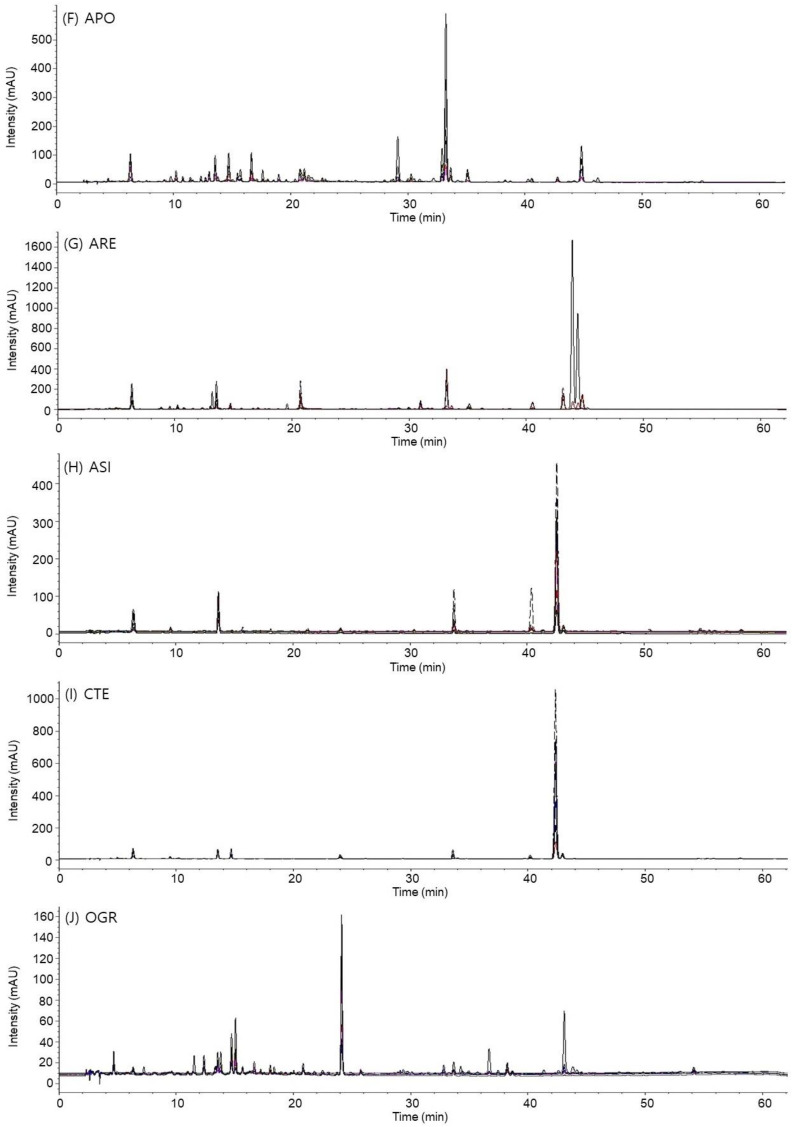
Overlapping chromatograms of representative samples from each species, labeled as follows: *Angelica acutiloba* (AAC, (**A**)), *A. biserrata* (ABI, (**B**)), *A. dahurica* (ADA, (**C**)), *A. decursiva* (ADE, (**D**)), *Angelica gigas* (AGI, (**E**)), *A. polymorpha* (APO, (**F**)), *A. reflexa* (ARE, (**G**)), *A. sinensis* (ASI, (**H**)), *Conioselinum tenuissimum* (CTE, (**I**)), and *Ostericum grossiserratum* (OGR, (**J**)).

The 46 marker compounds, encompassing 30 coumarins such as nodakenin (**5**), umbelliferone (**6**), xanthotoxol (**10**), marmesin (**12**), oxypeucedanin hydrate (**14**), decursinol (**15**), bergaptol (**16**), byakangelicin (**17**), psoralen (**18**), angelol B (**19**), angelol H (**20**), angelicin (**21**), xanthotoxin (**22**), angelol A (**23**), angelol G (**24**), bergapten (**25**), ostenol (**26**), byakangelicol (**28**), oxypeucedanin (**29**), columbianetin acetate (**30**), imperatorin (**34**), phellopterin (**36**), osthol (**37**), decursin (**38**), decursinol angelate (**39**), isoimperatorin (**40**), suberosin (**41**), columbianadin (**42**), praeruptorin B (**44**) and praeruptorin C (**46**), 6 phthalides including senkyunolide I (**9**), senkyunolide H (**11**), senkyunolide A (**32**), 3-*n*-butyl-phthalide (**33**), ligustilide (**35**), and levistilide A (**45**), 4 phenolic acids like chlorogenic acid (**1**), caffeic acid (**2**), ferulic acid (**7**), and benzoic acid (**8**), and 3 chromones including prim-O-glucosyl-cimifugin (**3**), cimifugin (**4**), and sec-O-glucosyl-hamaudol (**13**), along with a sesquiterpene (bisabolangelone (**27**)), a monolignol (coniferyl ferulate (**31**)), and a polyacetylene (falcarindiol (**43**)), were effectively separated on the chromatograms for quantification (Figure 2 and Figure S1). The calibration curves of these 46 marker compounds exhibited a linear correlation between serial concentrations and absolute areas with *r*^2^ values > 0.9992. The LODs ranged from 0.01 μg/mL to 0.62 μg/mL and LOQs ranged from 0.03 μg/mL to 2.07 μg/mL (as listed in Table S3). Intraday and interday precisions for the marker compounds were <2.0% of relative standard deviation (RSD) values (with accuracies ranging from 89.91% to 101.73%) and <6.0% of RSD values (with accuracies from 93.29% to 101.81%), respectively (Table S4). Recoveries of the marker compounds fell within the range of 92.72–111.13%, with RSD values < 8.6% (Table S5).

As depicted in Figure S2 and Tables S6–S15, the marker compounds were categorized into species-specific and non-specific markers. Cimifugin (**4**) and sec-O-glucosyl-hamaudol (**13**) were exclusively detected in the ARE samples, whereas angelol B (**19**), angelol H (**20**), angelicin (**21**), angelol G (**24**), and praeruptorin B (**44**) were found solely in the ABI samples.

Among the non-specific markers, xanthotoxol (**10**) and phellopterin (**36**) exhibited significantly higher levels in the ADA samples compared to both the AAC samples and APO samples, respectively. Similarly, the contents of angelol A (**23**), columbianetin acetate (**30**), and columbianadin (**42**) were notably elevated in the ABI samples compared to those in the ADA samples, ARE samples, and ADE samples, respectively. However, no significant quantitative differences were observed for prim-O-glucosyl-cimifugin (**3**) between the ADA and ARE samples nor for osthol (**37**) between the ABI and ARE samples.

Nodakenin (**5**) exhibited significantly higher contents in both the ADE and AGI samples, while umbelliferone (**6**) displayed elevated levels specifically in the ADE samples and benzoic acid (**8**) demonstrated increased content in both the ASI and CTE samples compared to the AAC samples. Moreover, the contents of marmesin (**12**), decursinol (**15**), decursin (**38**), and decursinol angelate (**39**) were notably higher in the AGI samples. Similarly, byakangelicin (**17**), byakangelicol (**28**), and imperatorin (**34**) exhibited elevated levels in the ADA samples, while xanthotoxin (**22**) was more abundant in the AAC samples. Additionally, bergapten (**25**) showed increased content in the ABI samples, while ostenol (**26**) and bisabolangelone (**27**) displayed higher levels in the ARE samples. Moreover, senkyunolide A (**32**) exhibited elevated content in the CTE samples and falcarindiol (**43**) demonstrated significantly higher levels in the OGR samples compared to other samples.

Chlorogenic acid (**1**) displayed significantly higher levels in the AGI and ARE samples, while ferulic acid (**7**) exhibited elevated content in the ARE and ASI samples. Senkyunolide I (**9**), senkyunolide H (**11**), ligustilide (**35**), and levistilide A (**45**) demonstrated increased levels in the ASI and CTE samples, while oxypeucedanin hydrate (**14**) and isoimperatorin (**40**) showed elevated contents in the ADA samples. Additionally, coniferyl ferulate (**31**) exhibited significantly higher levels in the APO and CTE samples compared to other samples. In contrast, caffeic acid (**2**) showed significantly lower contents in the ASI and CTE samples and 3-*n*-butyl-phthalide (**33**) demonstrated decreased levels in the AAC samples compared to other samples.

### 2.4. Chemometric Clustering Analysis

**Figure 3:**
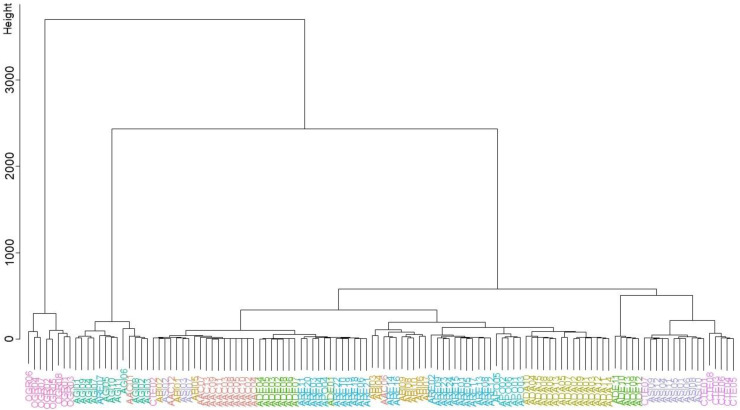
Dendrogram of samples, including *Angelica acutiloba* (AAC), *A. biserrata* (ABI), *A. dahurica* (ADA), *A. decursiva* (ADE), *Angelica gigas* (AGI), *A. polymorpha* (APO), *A. reflexa* (ARE), *A. sinensis* (ASI), *Conioselinum tenuissimum* (CTE), and *Ostericum grossiserratum* (OGR).

In the hierarchical clustering analysis (HCA), the majority of samples formed distinct clusters corresponding to their botanical species, with the occasional insertion of samples from other species. Notably, AAC samples (excluding AAC01, AAC06, and AAC12) and ABI05 clustered together, along with a close positioning of a mixture of samples (AAC12, ABI01, –02, ASI02, –03, –07, and CTE02). The remaining ABI samples were clustered with AAC06, ARE14, and ARE16. ADA samples formed a separate cluster without any interspersions. ADE samples were split into two distinct clusters within their species (ADE02−ADE08 vs. ADE09–ADE13). All AGI samples, along with AAC01 and ARE07, formed a distinct cluster. APO samples, except for APO04, also formed their own cluster. Although three samples (ARE07, ARE14, and ARE16) were positioned adjacent to other samples, ARE samples were divided into two separate clusters, closely associated with ADE samples and APO and ADA samples, respectively. The remaining ASI samples were combined with CTE01 and CTE07, near the cluster of CTE samples. OGR samples formed a separate cluster, distinctly different from other samples (Figure 3).

**Figure 4:**
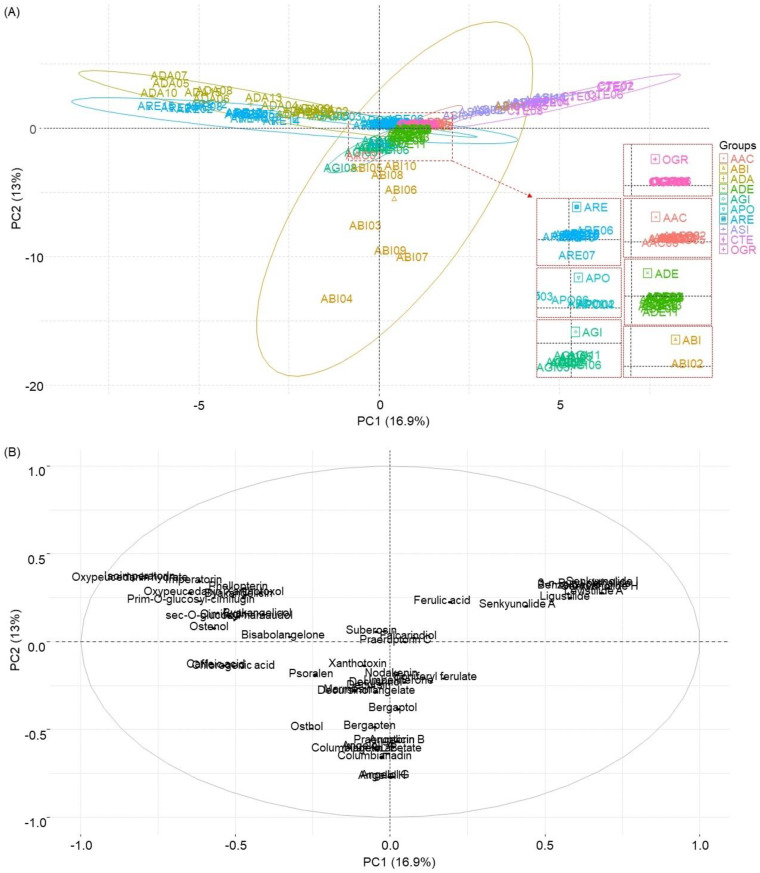
Principal component score plot (**A**) and loading plot (**B**), featuring *Angelica acutiloba* (AAC), *A. biserrata* (ABI), *A. dahurica* (ADA), *A. decursiva* (ADE), *Angelica gigas* (AGI), *A. polymorpha* (APO), *A. reflexa* (ARE), *A. sinensis* (ASI), *Conioselinum tenuissimum* (CTE), and *Ostericum grossiserratum* (OGR).

In the principal component (PC) score plot, most ADA samples and ARE samples exhibited negative PC1 scores and positive PC2 scores. Conversely, ASI and CTE samples displayed positive scores for both PC1 and PC2. The ABI samples, except for two samples, had negative PC2 scores but varied in their PC1 scores between positive and negative values. These sample distributions were distinctly discernible based on PC scores. However, there were compact distributions of AAC, ADE, AGI, APO, OGR, and some ARE samples near ‘zero’ PC scores. Similar to the clustering in Figure 3, the ARE samples were distributed as a Nam-Gang-hwal group including ARE07 and –20 and a Buk-Gang-hwal group including ARE13 and –14. Although samples were grouped by their respective species, there was notable overlap in their distributions (Figure 4).

### 2.5. Correlation Analysis

**Figure 5:**
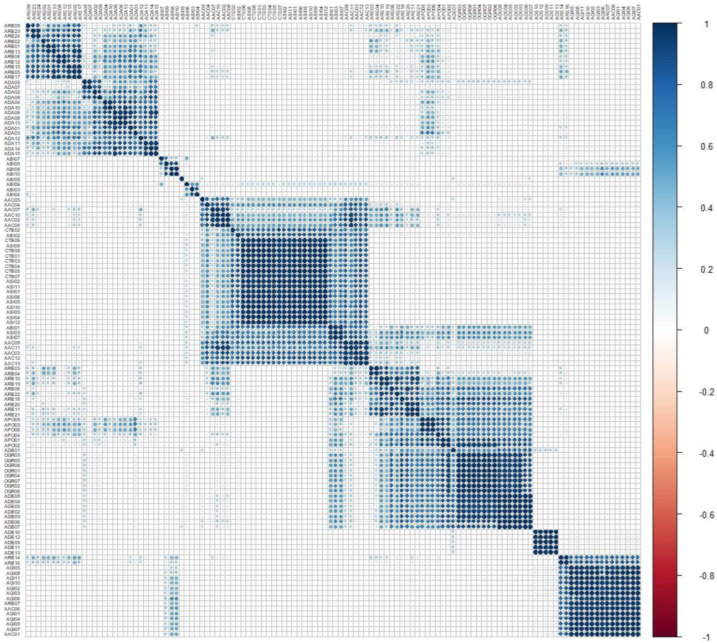
Graphic view of Pearson’s correlation coefficients among the samples, including *Angelica acutiloba* (AAC), *A. biserrata* (ABI), *A. dahurica* (ADA), *A. decursiva* (ADE), *Angelica gigas* (AGI), *A. polymorpha* (APO), *A. reflexa* (ARE), *A. sinensis* (ASI), *Conioselinum tenuissimum* (CTE), and *Ostericum grossiserratum* (OGR).

Chemical correlations among *Angelica* species were assessed by computing Pearson’s correlation coefficient (*r*) for both intra- and interspecies comparisons, as outlined in Table 2 and illustrated in Figure 5. Among the *Angelica* species, AGI, APO, ASI, CTE, and OGR samples exhibited notably high intraspecies coefficients, with mean and median *r* values > 0.9. Following closely were the ADA and AAC samples, displaying mean *r* values ranging from 0.6 to 0.8 and median *r* values ranging from 0.8 to 0.9. However, distinct outliers were observed among the samples such as AAC01, AAC06, ASI03, ASI07, and CTE02 in terms of intraspecies coefficients. In contrast, ADE and ARE samples showed lower mean *r* values of 0.4–0.5 and median *r* values of 0.3–0.4, respectively. The ABI samples exhibited the lowest mean and median *r* values of coefficients, both below 0.2 (Figure S3).

Interspecies correlations presented a wider range of values compared to intraspecies correlations. AAC samples displayed relatively higher mean and median coefficients with ASI and CTE samples (both *r* > 0.5), while coefficients with other samples were generally below 0.2 (except for ARE samples with mean *r* values > 0.2) and even showed negative values with ADA samples. ABI samples showed mean coefficients below 0.2, with most median values being negative. ADA samples exhibited mean and median *r* values ranging from 0.2 to 0.4 with APO and ARE samples but negative values with samples from other species. ADE samples displayed relatively higher mean coefficients with APO samples (*r* > 0.4) and OGR samples (*r* > 0.5) and lower mean values with ARE samples (*r* > 0.1). AGI samples showed coefficients mostly close to zero, with both positive and negative values, when compared to other samples. APO samples showed higher coefficients with OGR samples (mean *r* value > 0.8 and median *r* value > 0.7), followed by higher values with ARE samples (mean and median *r* values > 0.4). However, the coefficients of CTE samples with OGR samples were negative in both mean and median values (Figure S4).

Pearson’s correlation coefficient (*r*) analysis further confirmed the stronger associations of Nam-Gang-hwal samples with ADA samples (mean *r* value = 0.44) and Buk-Gang-hwal samples with AAC (mean *r* value = 0.34) and ADE samples (mean *r* value = 0.34), compared to the correlations of Buk-Gang-hwal samples with ADA samples (mean *r* value = 0.09) and Nam-Gang-hwal samples with AAC (mean *r* value = 0.13) and ADE samples (mean *r* values < 0), respectively. Meanwhile, APO and OGR samples displayed closer correlations with Buk-Gang-hwal samples (both mean *r* value > 0.5) than with Nam-Gang-hwal samples.

## 3. Discussion

Recently, entire cp genome analysis has been widely used for gene-based species discrimination or identification research. Those versatile characteristics make the cp region specifically applicable to plants, and its genetically informative feature is quite an attractive approach to botanical identification. However, the cp genome analysis has a limitation on the samples being processed via multiple steps. Therefore, DNA barcoding and phylogenetic analysis were used to identify the specific botanical species among samples of the *Angelica* genus. Previous studies highlighted the effectiveness of ITS as a robust tool, but analysis of the *psbA-trnH* region was also conducted in this study to increase result accuracy [22,23]. Despite some reported issues with indels in the *psbA-trnH* region due to its non-coding region, it remains an efficient tool for analyzing herbal medicines due to its relatively short length and abundant variation compared to other cpDNA barcode regions such as *rbcL* and *MatK* [24]. The results of this study also revealed that while the *psbA-trnH* sequence length is approximately half that of ITS, the variation in sequence between species is similar to that of ITS.

The taxonomic classification of *A. reflexa* (Korean name: Gang-hwal), of which the Korean botanical name is the same as the Korean herbal name, has been quite complex. It was initially classified as *A. koreana* by Maximowicz (1886) [25] and later transferred to the genus *Ostericum* (*O. koreanum*) by Kitagawa (1936) [26] due to its external morphological similarity. Subsequently, Kitagawa (1971) [27] recognized the taxonomic identity between *A. koreana* (=*O. koreanum*) and *O. grossiserratum*, treating *A. koreana* (=*O. koreanum*) as a synonym of *O. grossiserratum*. This is the reason why *O. koreanum* still remains a synonym of *O. grossiserratum*. However, molecular phylogenetic study indicated that *A. koreana* (=*O. koreanum*) may be independent from *O. grossiserratum* [28]. Furthermore, both external morphological examination and molecular phylogeny using nuclear DNA ITS sequences have shown that commercial medicinal plants cultivated as Gang-hwal are neither *A. koreana* nor *O. grossiserratum* [29]. After careful observation of morphological and anatomical characters and examination of relevant specimens, Lee et al. (2013) [5] ultimately proposed this as a new species of Angelica, named *A. reflexa*. Some researchers still consider Gang-hwal to be *A. genuflexa* due to strong morphological similarity and regard *A. reflexa* as a synonym of *A. genuflexa* [29]. However, careful observation [5] and our DNA barcode analysis support the difference between *A. reflexa* and *A. genuflexa*. As shown in Figure 1, all *A. reflexa* (ARE) samples were located within the ‘*Angelica*’ clade, not within the ‘Ostericum’ clade, while *O. grossiserratum* was clearly classified as genus Ostericum and separated from *A. genuflexa*. According to the results of phylogenetic analysis, *A. genuflexa* was closely grouped with *A. biserrata* (ABI) and *A. polymorpha* (APO) rather than *A. reflexa*. Moreover, the herbal samples of Gang-hwal acquired for this study were clearly identified as *A. reflexa*, not *O. koreanum* or *O. grossiserratum*.

Interesting findings were observed for the three *Angelica* species commonly used as Dang-gwi (Figure 1). Despite *A. gigas* (AGI) and *A. acutiloba* (AAC) being grouped within the same *Angelica* clade, they are distinctly separated into different clusters (AGI clustered with ADE). Additionally, *A. sinensis* (ASI) was clustered with *L. sinense* and *L. jeholense*, positioned within the *Ligusticum* clade including CTE samples rather than the *Angelica* clade.

Quantification of the marker compounds in genetically identified *Angelica* samples was employed to explore chemical relationships among *Angelica* species using various chemometric tools. Angelol B (**19**), angelol H (**20**), angelicin (**21**), angelol A (**23**), angelol G (**24**), bergapten (**25**), columbianetin acetate (**30**), columbianadin (**42**), and praeruptorin B (**44**) were possibly responsible for discerning the ABI samples [8,30]. The ADA samples were characterized by xanthotoxol (**10**), oxypeucedanin hydrate (**14**), byakangelicin (**17**), byakangelicol (**28**), imperatorin (**34**), phellopterin (**36**), and isoimperatorin (**40**) [8,31,32]. Marmesin (12), decursinol (**15**), decursin (**38**), and decursinol angelate (**39**) were notable discerning compounds of the AGI samples [10,11]. Senkyunolide A (**32**), 3-n-butyl-phthalide (**33**), ligustilide (**35**), and levistilide A (**45**) were distinctive in separating the CTE samples [33,34]. Cimifugin (**4**), sec-O-glucosyl-hamaudol (**13**), ostenol (**26**), and bisabolangelone (**27**) were distinct discerning compounds in the ARE samples [35,36]. Falcarindiol (**43**) was apparently discernable in the OGR samples. Ferulic acid (**7**), senkyunolide I (**9**), senkyunolide H (**11**), ligustilide (**35**), and levistilide A (**45**), which have been known as the main constituents of *A. sinensis* [11,37,38], affected the discerning of ASI samples as well as CTE samples. In the HCA dendrogram, the ABI, ADE, and ARE samples were divided into two separate groups. The separation of the ABI samples into two different clusters in the dendrogram and the broad distributions by the PC1 and PC2 scores might be attributed to the lowest intraspecies correlations among the ABI samples, which were represented by the lowest correlation coefficients (mean *r* value < 0.2). The chemical division of the ADE samples as well as the ABI samples could be influenced by the differences in their geographic origins, as geographical variations among different countries can impact the chemical compositions of *Angelica* species [39].

The roots of *A. reflexa* are utilized in Korean herbal markets to produce two distinct types of Gang-hwal, known as ‘Nam-Gang-hwal’ through seed-propagation and ‘Buk-Gang-hwal’ via root propagation [40,41]. These differing cultivation methods yield variations in the chemical compositions between Nam-Gang-hwal and Buk-Gang-hwal [35]. In this study, the ARE samples were also categorized into the Nam-Gang-hwal group and the Buk-Gang-hwal group. These differences in sample types led to the division of ARE samples into separate groups in both the HCA dendrogram and the PC score plot. Additionally, Nam-Gang-hwal samples exhibited a closer chemical relationship with the ADA samples, while Buk-Gang-hwal samples showed a closer relationship with the ADE and AAC samples, forming clusters of closely related samples [42].

The chemical relationships among three *Angelica* species of Dang-gwi were depicted differently in the dendrogram and Pearson’s coefficients. In the dendrogram, the AAC samples and ASI samples were distinctly separated into individual clusters, while the AGI samples formed a secondary cluster, situated apart from the clusters of AAC and ASI samples. Pearson’s coefficients also highlighted the chemical heterogeneity of AGI samples from AAC and ASI samples, with mean *r* values < 0.2. However, a higher r-value of AAC–ASI (mean *r* value > 0.5) indicated a closer chemical relationship between AAC and ASI samples compared to the combination with AGI samples. Previous studies have reported chemical heterogeneity among three *Angelica* species of Dang-gwi, but in contrast to these findings, *A. gigas* and *A. acutiloba* exhibited a closer relationship than with *A. sinensis* samples [10,14].

Overall, the chemical relationship between ASI and CTE samples appears to reflect their phylogenetic relevance, despite their difference in therapeutic activities. The ARE samples (i.e., Nam-Gang-hwals) exhibited chemical and phylogenetic relevance with ADA samples, while ARE samples (i.e., Buk-Gang-hwals) showed relevance with AAC and ADE samples. The APO samples, classified as non-medicinal species, displayed partial chemical and genetic relevance with ABI, ADA, and ARE samples and a stronger correlation with OGR samples. Although the OGR samples, another non-medicinal species, exhibited the lowest genetic relationship with other species, their chemical relationship with other species varied, either parallel or opposite to the genetic results, depending on the statistical tools used.

This study has several limitations: (1) the unequal distribution of samples among species; (2) the limited representativeness of the selected 46 marker compounds to whole chemical characteristics of all species samples; (3) inconsistencies in the chemical relationships among the samples in the chemometric analyses; and (4) the lack of comparison between ARE samples and their therapeutic analogous *Notopterygium* species. Despite these limitations, this study represents the first attempt to genetically authenticate and chemically classify medicinal *Angelica* species, as well as non-medicinal species. The quantitative explanation of the differences among the *Angelica* species, including the alternative use of non-medicinal species, would be further supported by pharmacological study.

## 4. Materials and Methods

### 4.1. Plant Materials

**Figure 6:**
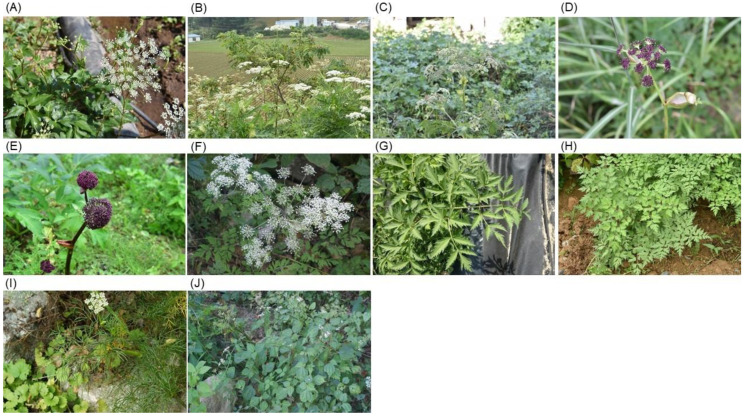
Botanical pictures of *Angelica* species. (**A**) *Angelica acutiloba*, (**B**) *A. biserrata*, (**C**) *A. dahurica*, (**D**) *A. decursiva*, (**E**) *A. gigas*, (**F**) *A. polymorpha*, (**G**) *A. reflexa*, (**H**) *A. sinensis*, (**I**) *Conioselinum tenuissimum*, and (J) *Ostericum grossiserratum*. The photo of *A. reflexa* was provided by KIOM.

The roots or rhizomes of various *Angelica* species, including *A. acutiloba* (AAC), *A. biserrata* (ABI), *A. dahurica* (ADA), *A. decursiva* (ADE), *Angelica gigas* (AGI), *A. polymorpha* (APO), *A. reflexa* (ARE), *A. sinensis* (ASI), *Conioselinum tenuissimum* (CTE), and *Ostericum grossiserratum* (OGR), were mostly provided by the Korea Institute of Oriental Medicine (KIOM; Naju, Jeonnam, Republic of Korea). Some were purchased from herbal companies and others were collected from their natural habitats in Korea and China (Table 1). The samples of ARE01, –02, –05, –08, –09, –12, –15, –16, –17, –23, and –24 were obtained as ‘Nam-Gang-hwal’, those of ARE03, –04, –06, –10, –11, –18, –19, –21, and –22 were obtained as ‘Buk-Gang-hwal’, and those of ARE07, –13, –14, and –20 had a lack of information. All samples were firstly authenticated using organoleptic examination by herbal experts of KIOM and authors. Voucher specimens (2022-PNUKM-AAC01–AAC13, ABI01–ABI10, ADA01–ADA15, ADE01–ADE13, AGI01–AGI11, APO01–APO06, ARE01–ARE24, ASI01–ASI12, CTE01–CTE08, and OGR01–OGR08) have been deposited at the School of Korean Medicine, Pusan National University (Yangsan, Republic of Korea). The botanical pictures of each species are shown in Figure 6.

### 4.2. Preparation of Genomic DNA

The genomic DNA was extracted from the samples following the instructions provided in the NucleoSpin^®^ Plant II kit manual (Macherey-Nagel, Dueren, Germany). This process involved the use of a PL1 lysis buffer during a lysis step that lasted at least 2 h. For certain samples, an additional step was incorporated, which involved the use of 10% cetyltrimethyl ammonium bromide (CTAB) and 0.7M NaCl. This extra step was employed to remove phenolic compounds and polysaccharides after the extraction of DNA using the kit.

### 4.3. PCR Amplification for DNA Barcode Analysis

PCR amplification for ITS was conducted using a T-personal cycler (Biometra, Jena, Germany). Briefly, a 600 nM primer set of ITS1 (5′-TCCGTAGGTGAACCTGCGG-3′) and ITS4 (5′-TCCTCCGCTT ATTGATATGC-3′) [43], 1X AccuPower^®^ GoldHotStart Taq PCR PreMix (Bioneer, Daejeon, Republic of Korea), and 30 ng of genomic DNA were used for PCR amplification. The PCR cycling conditions included a pre-denaturation process (95 °C, 5 min), followed by a denaturation process (95 °C, 30 s), annealing progress (52 °C, 30 s), and extension process (72 °C, 40 s) for 36 cycles and a final extension process (72 °C, 5 min). For chloroplast DNA barcoding regions, the trnH2 (5′-CGCGCATGGTGGATTCACAATCC-3′)/psbAF (5′-GTTATGCATGAACGTAATGCTC-3′) set was used for *psbA-trnH* regions [44]. The amplified PCR product was separated from other gradients using 1.5% agarose gel electrophoresis and stained with Safe-whiteTM (abm, Richmond, Canada). The amplified products were analyzed using MyImage (Seoulin Biotechnology, Seongnam, Republic of Korea).

### 4.4. Determination of DNA Sequences of PCR Product

The PCR product, separated from the agarose gel using the MagListo™ 5M PCR/Gel Purification Kit (Bioneer, Daejeon, Republic of Korea), was cloned using the TOPcloner™ TA Kit (Enzynomics, Daejeon, Republic of Korea). The DNA sequences of the cloned PCR product were then determined through analysis performed by Bioneer (Daejeon, Republic of Korea).

### 4.5. Analysis of DNA Sequences and Preparation of Phylogenetic Tree

DNA sequences were analyzed using ClustalW multiple sequence alignment (Bioedit, v7.7.1) and confirmed with multiple sequence alignment in MAFFT (MAFFT, v7) [45]. To verify the polymorphisms represented by IUPAC symbols in the sequence data, all sequences were generated at least twice. The chromatograms of nucleotide sequences, provided by the Bioneer sequencing service, were compared. Evolutionary analyses were conducted in MEGA X (ver. 10.0.5). Phylogenetic analysis of two concatenated DNA barcode regions (ITS and *psbA-trnH*) was constructed using the PhyML + SMS/OneClick method, which showed a workflow of MAFFT, BMGE, and PhyML + SMS (maximum likelihood-based inference of phylogenetic trees with Smart Model Selection) [46]. All analyzed sequences were compared with NCBI GenBank using BLAST [47]. Newly determined nucleotide sequences were deposited in NCBI GenBank. Two other subfamilies of the Apiaceae, *Eryngium regnellii* (Saniculoideae) and *Centella asiatica* (Mackinlayoideae), were used as outgroups. NCBI data used in the phylogenetic tree analysis were represented with the accession number and scientific names listed in the NCBI database.

### 4.6. Chemicals and Reagents

Analytical-grade acetonitrile, methanol, and water were procured from J.T. Baker Inc. (Phillipsburg, NJ, USA), while trifluoroacetic acid (TFA) was purchased from Sigma-Aldrich (St. Louis, MO, USA). Forty-six marker compounds, including chlorogenic acid (**1**), caffeic acid (**2**), prim-O-glucosyl-cimifugin (**3**), cimifugin (**4**), nodakenin (**5**), umbelliferone (**6**), ferulic acid (**7**), benzoic acid (**8**), senkyunolide I (**9**), xanthotoxol (**10**), senkyunolide H (**11**), marmesin (**12**), sec-O-glucosyl-hamaudol (**13**), oxypeucedanin hydrate (**14**), decursinol (**15**), bergaptol (**16**), byakangelicin (**17**), psoralen (**18**), angelol B (**19**), angelol H (**20**), angelicin (**21**), xanthotoxin (**22**), angelol A (**23**), angelol G (**24**), bergapten (**25**), ostenol (**26**), bisabolangelone (**27**), byakangelicol (**28**), oxypeucedanin (**29**), columbianetin acetate (**30**), coniferyl ferulate (**31**), senkyunolide A (**32**), 3-n-butyl-phthalide (**33**), imperatorin (**34**), ligustilide (**35**), phellopterin (**36**), osthol (**37**), decursin (**38**), decursinol angelate (**39**), isoimperatorin (**40**), suberosin (**41**), columbianadin (**42**), falcarindiol (**43**), praeruptorin B (**44**), levistilide A (**45**), and praeruptorin C (**46**), were acquired from ChemFace (Wuhan, China). The chemical structures of these marker compounds are depicted in Figure S5.

### 4.7. Analytical Sample Preparation

Before use, all samples (each triplicate) were thoroughly dried and then ground to a powder, which was homogenized through a 500 μm testing sieve (Chunggyesanggong-sa; Gunpo, Republic of Korea). A precise weight of the powder (500 mg) was extracted with 5 mL of methanol for 30 min using an ultrasonic extractor (Power Sonic 520; Hwashin Tech, Daegu, Republic of Korea). The extract was then centrifuged at 10,000 rpm for 10 min. The supernatant was transferred to a 1.5-mL microtube and gently dried using a nitrogen-blowing concentrator (MGS2200; Eyela, Tokyo, Japan). The residue was re-dissolved in HPLC-grade methanol at a concentration of 10,000 μg/mL and filtered through a 0.2 μm syringe filter (BioFact, Daejeon, Republic of Korea) before HPLC injection.

### 4.8. HPLC Analytical Conditions

The quantitative analysis of the marker compounds was performed using an Agilent 1200 liquid chromatography system (Agilent Technologies, Palo Alto, CA, USA), equipped with an autosampler, degasser, quaternary solvent pump, and diode array detector. The data acquired were processed using Chemstation software (Rev. B. 04. 03.; Agilent Technologies Inc., USA). A Capcell Pak Mg II C_18_ column (4.6 mm × 250 mm, 5 μm; Shiseido, Tokyo, Japan) was used to separate forty-six marker compounds at 35 °C with a flow rate of 1 mL/min and an injection volume of 10 μL. The mobile phases were pumped via gradient elution by mixing water containing 0.1% TFA (solvent A) and acetonitrile (solvent B). The percentages of mobile phase (solvent B) with equivalent retention times were as follows: 15% for 0–2 min, 15–50% for 2–30 min, 50–50% for 30–32 min, 50–75% for 32–55 min, and 75% for 55–58 min, and then re-equilibrated to 15% until the end of the analysis. The detection wavelength of the diode-array detector was set at 230, 250, 270, 280, and 325 nm.

### 4.9. Validation of the HPLC Method

The stock solution was prepared by dissolving each marker compound in methanol at a concentration of 1000 μg/mL, and the working solution for the calibration curve was generated by serial dilution of the stock solution to seven different concentrations. The correlation coefficients (*r*^2^) were determined to assess the linearity of the calibration curve. The limit of detection and the limit of quantification were established as signal-to-noise (S/N) ratios of 3 and 10, respectively.

Precision, indicative of the repeatability of the analytical method, was assessed by analyzing low and high concentrations of the stock solutions three times within one day (intraday precision) and over three consecutive days (interday precision). Precision was expressed as relative standard deviations (RSDs): RSD (%) = (standard deviation/mean) × 100.

The accuracy of the analytical method, represented by recovery, was evaluated by adding low and high concentrations of the marker compounds to the sample solutions. The equation for calculating recovery was as follows: Recovery (%) = [(detected concentration-initial concentration)/spiked concentration] × 100.

### 4.10. Chemometric Statistical Analysis

The differences in the contents of the marker compounds among the species were assessed using the Kruskal–Wallis rank sum test and the Bonferroni post hoc test. Statistical significance was considered at least *p* < 0.05. The chemical relationships among the samples were investigated using principal component analysis (PCA) and hierarchical clustering analysis (HCA) with a matrix comprising rows (representing the samples) and columns (representing the content of the marker compounds). Pearson’s correlation coefficients (r) were calculated to evaluate both inter- and intraspecies correlations (−1 < *r* < 1). All statistical analyses were conducted using open-source software R (v. 4.3.0; The R Foundation for Statistical Computing).

## 5. Conclusions

In this study, a total of 120 samples from 8 medicinal *Angelica* species and 2 non-medicinal species were phylogenetically authenticated using DNA barcoding analysis with ITS and *psbA-trnH* regions. HPLC analytical methods were successfully established for the quantification of 46 marker compounds, including 30 coumarins, 6 phthalides, 4 phenolic acids, 3 chromones, 1 sesquiterpene, 1 monolignol, and 1 polyacetylene. Chemometric analysis was employed to investigate the chemical relationships among the *Angelica* species. The *A. reflexa* and *A. biserrata* samples each exhibited species-specific marker compounds. Surprisingly, *A. sinensis* samples showed the closest chemical and phylogenetic relationship with *C. tenuissimum samples*, rather than their therapeutic analogs, *A. gigas* and *A. acutiloba*. Within *A. reflexa* samples, which were categorized into Nam-Gang-hwals and Buk-Gang-hwals, chemical similarities were observed with *A. dahurica* samples and *A. acutiloba*−*A. decursiva* samples, respectively. The chemical and genetic proximity of *A. polymorpha* to other medicinal *Angelica* species warrants further investigation into its potential medicinal uses.

## Figures and Tables

**Table 1:** Species identification of the samples based on DNA barcode analysis of the ITS and *psbA-trnH* regions.

Code	Species	Geographic Origin	Acquisition	Code	Species	Geographic Origin	Acquisition
AAC-01	*Angelica acutiloba*	Jecheon, Korea	Provided	APO-01	*A. polymorpha*	Wonju, Korea	Collected
AAC-02	*A. acutiloba*	China	Provided	APO-02	*A. polymorpha*	Wonju, Korea	Collected
AAC-03	*A. acutiloba*	PyeongChang, Korea	Provided	APO-03	*A. polymorpha*	Wonju, Korea	Collected
AAC-04	*A. acutiloba*	Korea	Provided	APO-04	*A. polymorpha*	Wonju, Korea	Collected
AAC-05	*A. acutiloba*	Japan	Provided	APO-05	*A. polymorpha*	Wonju, Korea	Collected
AAC-06	*A. acutiloba*	China	Provided	APO-06	*A. polymorpha*	Wonju, Korea	Collected
AAC-07	*A. acutiloba*	Korea	Provided	ARE-01	*A. reflexa*	Korea	Provided (NG)
AAC-08	*A. acutiloba*	-	Provided	ARE-02	*A. reflexa*	Jecheon, Korea	Provided (NG)
AAC-09	*A. acutiloba*	Yeongju, Korea	Provided	ARE-03	*A. reflexa*	Yeongju, Korea	Provided (BG)
AAC-10	*A. acutiloba*	Yeongju, Korea	Provided	ARE-04	*A. reflexa*	Korea	Provided (BG)
AAC-11	*A. acutiloba*	Yeongju, Korea	Provided	ARE-05	*A. reflexa*	Uljin, Korea	Provided (NG)
AAC-12	*A. acutiloba*	Yeongju, Korea	Provided	ARE-06	*A. reflexa*	Korea	Provided (BG)
AAC-13	*A. acutiloba*	Korea	Purchased	ARE-07	*A. reflexa*	Pyeongchang, Korea	Provided (-)
ABI-01	*A. biserrata*	Gansu, China	Provided	ARE-08	*A. reflexa*	-	Provided (NG)
ABI-02	*A. biserrata*	China	Provided	ARE-09	*A. reflexa*	-	Provided (NG)
ABI-03	*A. biserrata*	Hunan, China	Provided	ARE-10	*A. reflexa*	-	Provided (BG)
ABI-04	*A. biserrata*	Sichuan, China	Provided	ARE-11	*A. reflexa*	Bonghwa, Korea	Provided (BG)
ABI-05	*A. biserrata*	Korea	Provided	ARE-12	*A. reflexa*	Pyeongchang, Korea	Provided (NG)
ABI-06	*A. biserrata*	Hubei, China	Provided	ARE-13	*A. reflexa*	Pyeongchang, Korea	Provided (-)
ABI-07	*A. biserrata*	Guangxi, China	Provided	ARE-14	*A. reflexa*	Jecheon, Korea	Provided (-)
ABI-08	*A. biserrata*	Anhui, China	Provided	ARE-15	*A. reflexa*	Korea	Provided (NG)
ABI-09	*A. biserrata*	-	Provided	ARE-16	*A. reflexa*	Pyeongchang, Korea	Provided (NG)
ABI-10	*A. biserrata*	-	Provided	ARE-17	*A. reflexa*	Korea	Provided (NG)
ADA-01	*A. dahurica*	Korea	Provided	ARE-18	*A. reflexa*	Bonghwa, Korea	Provided (BG)
ADA-02	*A. dahurica*	China	Provided	ARE-19	*A. reflexa*	Korea	Provided (BG)
ADA-03	*A. dahurica*	China	Provided	ARE-20	*A. reflexa*	Bonghwa, Korea	Provided (-)
ADA-04	*A. dahurica*	Gunwi, Korea	Provided	ARE-21	*A. reflexa*	Korea	Provided (BG)
ADA-05	*A. dahurica*	Sichuan, China	Provided	ARE-22	*A. reflexa*	Namwon, Korea	Provided (BG)
ADA-06	*A. dahurica*	Yeongyang, Korea	Provided	ARE-23	*A. reflexa*	Yeongcheon, Korea	Provided (NG)
ADA-07	*A. dahurica*	Sichuan, China	Provided	ARE-24	*A. reflexa*	Uljin, Korea	Provided (NG)
ADA-08	*A. dahurica*	Yeongyang, Korea	Provided	ASI-01	*A. sinensis*	Gansu, China	Provided
ADA-09	*A. dahurica*	China	Provided	ASI-02	*A. sinensis*	Anhui, China	Provided
ADA-10	*A. dahurica*	Andong, Korea	Provided	ASI-03	*A. sinensis*	-	Provided
ADA-11	*A. dahurica*	Jecheon, Korea	Provided	ASI-04	*A. sinensis*	China	Purchased
ADA-12	*A. dahurica*	Yeongju, Korea	Provided	ASI-05	*A. sinensis*	China	Purchased
ADA-13	*A. dahurica*	Andong, Korea	Provided	ASI-06	*A. sinensis*	-	Purchased
ADA-14	*A. dahurica*	Yeongcheon, Korea	Provided	ASI-07	*A. sinensis*	-	Purchased
ADA-15	*A. dahurica*	Namwon, Korea	Provided	ASI-08	*A. sinensis*	-	Purchased
ADE-01	*A. decursiva*	-	Provided	ASI-09	*A. sinensis*	-	Purchased
ADE-02	*A. decursiva*	-	Purchased	ASI-10	*A. sinensis*	-	Purchased
ADE-03	*A. decursiva*	-	Purchased	ASI-11	*A. sinensis*	-	Purchased
ADE-04	*A. decursiva*	-	Purchased	ASI-12	*A. sinensis*	-	Purchased
ADE-05	*A. decursiva*	-	Purchased	CTE-01	*Conioselinum tenuissimum*	Korea	Provided
ADE-06	*A. decursiva*	-	Purchased	CTE-02	*C. tenuissimum*	Korea	Provided
ADE-07	*A. decursiva*	China	Purchased	CTE-03	*C. tenuissimum*	Korea	Provided
ADE-08	*A. decursiva*	China	Purchased	CTE-04	*C. tenuissimum*	Yeongyang, Korea	Provided
ADE-09	*A. decursiva*	Busan, Korea	Collected	CTE-05	*C. tenuissimum*	Yeongyang, Korea	Provided
ADE-10	*A. decursiva*	Busan, Korea	Collected	CTE-06	*C. tenuissimum*	Yeongyang, Korea	Provided
ADE-11	*A. decursiva*	Busan, Korea	Collected	CTE-07	*C. tenuissimum*	Jeongseon, Korea	Provided
ADE-12	*A. decursiva*	Busan, Korea	Collected	CTE-08	*C. tenuissimum*	Korea	Purchased
ADE-13	*A. decursiva*	Busan, Korea	Collected	OGR-01	*Ostericum grossiserratum*	Wonju, Korea	Collected
AGI-01	*A. gigas*	China	Provided	OGR-02	*O. grossiserratum*	Wonju, Korea	Collected
AGI-02	*A. gigas*	Korea	Provided	OGR-03	*O. grossiserratum*	Wonju, Korea	Collected
AGI-03	*A. gigas*	Gyeongbuk, Korea	Provided	OGR-04	*O. grossiserratum*	Wonju, Korea	Collected
AGI-04	*A. gigas*	Pyeongchang, Korea	Provided	OGR-05	*O. grossiserratum*	Wonju, Korea	Collected
AGI-05	*A. gigas*	Pyeongchang, Korea	Provided	OGR-06	*O. grossiserratum*	Wonju, Korea	Collected
AGI-06	*A. gigas*	-	Provided	OGR-07	*O. grossiserratum*	Wonju, Korea	Collected
AGI-07	*A. gigas*	-	Provided	OGR-08	*O. grossiserratum*	Wonju, Korea	Collected
AGI-08	*A. gigas*	Bonghwa, Korea	Provided				
AGI-09	*A. gigas*	Pyeongchang, Korea	Provided				
AGI-10	*A. gigas*	Pyeongchang, Korea	Provided				
AGI-11	*A. gigas*	Korea	Purchased				

‘-’, unknown. ‘Provided’, the samples were provided from the Herbal Medicine Resources Research Center, KIOM (Korea). ‘Purchased’, the samples were purchased from herbal companies in Korea. ‘Provided (NG)’, the samples were provided as the name of ‘Nam-Gang-hwal’. ‘Provided (BG)’, the samples were provided as the name of ‘Buk-Gang-hwal’. ‘Collected’, the samples were collected from wild habitats.

**Table 2:** Summary of the Pearson’s correlation coefficients of the samples.

		AAC	ABI	ADA	ADE	AGI	APO	ARE	ASI	CTE	OGR
AAC	Mean	0.6180									
	Median	0.8351									
ABI	Mean	0.1521	0.1897								
	Median	0.0410	0.0626								
ADA	Mean	−0.0179	−0.0969	0.7723							
	Median	−0.0431	−0.1056	0.8149							
ADE	Mean	0.1096	0.0325	−0.0045	0.4749						
	Median	0.1035	−0.0432	−0.0400	0.3720						
AGI	Mean	0.1235	0.0829	−0.0942	0.0514	0.9824					
	Median	−0.0312	−0.0685	−0.0977	−0.0296	0.9921					
APO	Mean	0.1321	0.0366	0.3481	0.4330	−0.0632	0.9248				
	Median	0.1331	−0.0328	0.3410	0.6627	−0.0657	0.9161				
ARE	Mean	0.2128	0.0238	0.2600	0.1289	0.0782	0.4050	0.4743			
	Median	0.1872	−0.0288	0.2008	−0.0119	−0.0352	0.4234	0.4026			
ASI	Mean	0.5459	0.1659	−0.0658	0.0457	−0.0525	0.0799	0.0075	0.9086		
	Median	0.5913	−0.0599	−0.0794	0.0025	−0.0528	−0.0069	−0.0288	0.9935		
CTE	Mean	0.5257	0.1656	−0.0841	−0.0330	−0.0497	−0.0309	−0.0421	0.9131	0.9388	
	Median	0.5533	−0.0408	−0.0805	−0.0293	−0.0473	−0.0312	−0.0538	0.9738	0.9848	
OGR	Mean	0.1357	0.0616	0.0775	0.5441	−0.0370	0.8105	0.2419	0.1299	−0.0171	0.9999
	Median	0.1290	−0.0491	0.0216	0.8938	−0.0371	0.7678	0.0045	0.0354	−0.0109	0.9999

AAC, *Angelica acutiloba*; ABI, *A. biserrata*; ADA, *A. dahurica*; ADE, *A. decursiva*; AGI, *Angelica gigas*; APO, *A. polymorpha*; ARE, *A. reflexa*; ASI, *A. sinensis*; CTE, *Conioselinum tenuissimum*; OGR, *Ostericum grossiserratum*.

## Data Availability

The original contributions presented in the study are included in the Supplementary Materials.

## Supplementary Materials

The following supporting information can be downloaded at: https://www.mdpi.com/article/10.3390/plants13091252/s1, Figure S1: Single chromatograms of 46 marker compounds; Figure S2: Comparison of the contents of the marker compounds among the samples. *Angelica acutiloba* (AAC), *A. biserrata* (ABI), *A. dahurica* (ADA), *A. decursiva* (ADE), *Angelica gigas* (AGI), *A. polymorpha* (APO), *A. reflexa* (ARE), *A. sinensis* (ASI), *Conioselinum tenuissimum* (CTE), and Ostericum grosseratum (OGR); Figure S3: Intraspecies Pearson’s correlation coefficients among the samples; Figure S4: Interspecies Pearson’s correlation coefficients among the samples; Figure S5: Chemical structures of 46 marker compounds; Table S1: The sequence identity matrix and sequence difference count matrix of ITS nucleotide sequences in Table 1; Table S2: The sequence identity matrix and sequence difference count matrix of *psbA-trnH* nucleotide sequences in Table 1; Table S3: Regression equation, linear range, correlation coefficient, limit of detection, and limit of quantification of the marker compounds; Table S4: Intra- and interday precisions of the marker compounds; Table S5: Recoveries of the marker compounds (n = 3); Table S6: Mean amounts of the marker compounds in the methanol extracts of *Angelica acutiloba* samples (mg/g); Table S7: Mean amounts of the marker compounds in the methanol extracts of *A. biserrata* samples (mg/g); Table S8: Mean amounts of the marker compounds in the methanol extracts of *A. dahurica* samples (mg/g); Table S9: Mean amounts of the marker compounds in the methanol extracts of *A. decursiva* samples (mg/g); Table S10: Mean amounts of the marker compounds in the methanol extracts of *A. gigas* samples (mg/g); Table S11: Mean amounts of the marker compounds in the methanol extracts of *A. polymorpha* samples (mg/g); Table S12: Mean amounts of the marker compounds in the methanol extracts of *A. reflexa* samples (mg/g); Table S13: Mean amounts of the marker compounds in the methanol extracts of *A. sinensis* samples (mg/g); Table S14: Mean amounts of the marker compounds in the methanol extracts of *Conioselinum tenuissimum* samples (mg/g); Table S15: Mean amounts of the marker compounds in the methanol extracts of Ostericum grosserratum samples (mg/g).
