# Supplementary material for: Chemical Relationship among Genetically Authenticated Medicinal Species of Genus Angelica
Source: Plants (Basel). 2024 Apr 30;13(9):1252. doi: 10.3390/plants13091252 (PMC11085054; doi:10.3390/plants13091252)
Supplement: Supplementary file 1 [file plants-13-01252-s001.zip › plants-2968199-supplementary.pdf]

Supplementary materials

Chemical Relationship among Genetically Authenticated Medicinal Species of Genus Angelica

Jung-Hoon Kim[1,‡], Eui-Jeong Doh[2,‡], Han-Young Kim[3], Guemsan Lee[2,4*],

[1]Division of Pharmacology, School of Korean Medicine, Pusan National University, Yangsan, 50612, Republic of Korea

[2]Research Center of Traditional Korean Medicine, Wonkwang University, Iksan, 54538, Republic of Korea

[3]School of Korean Medicine, Pusan National University, Yangsan, 50612, Republic of Korea

[4]Department of Herbology, College of Korean Medicine, Wonkwang University, Iksan, 54538, Republic of Korea

[*]Corresponding author at: Department of Herbology, College of Korean Medicine, Wonkwang University, Iksan, 54538, Republic of Korea

E-mail: rasfin@wku.ac.kr

[‡] These authors contributed equally to this work.

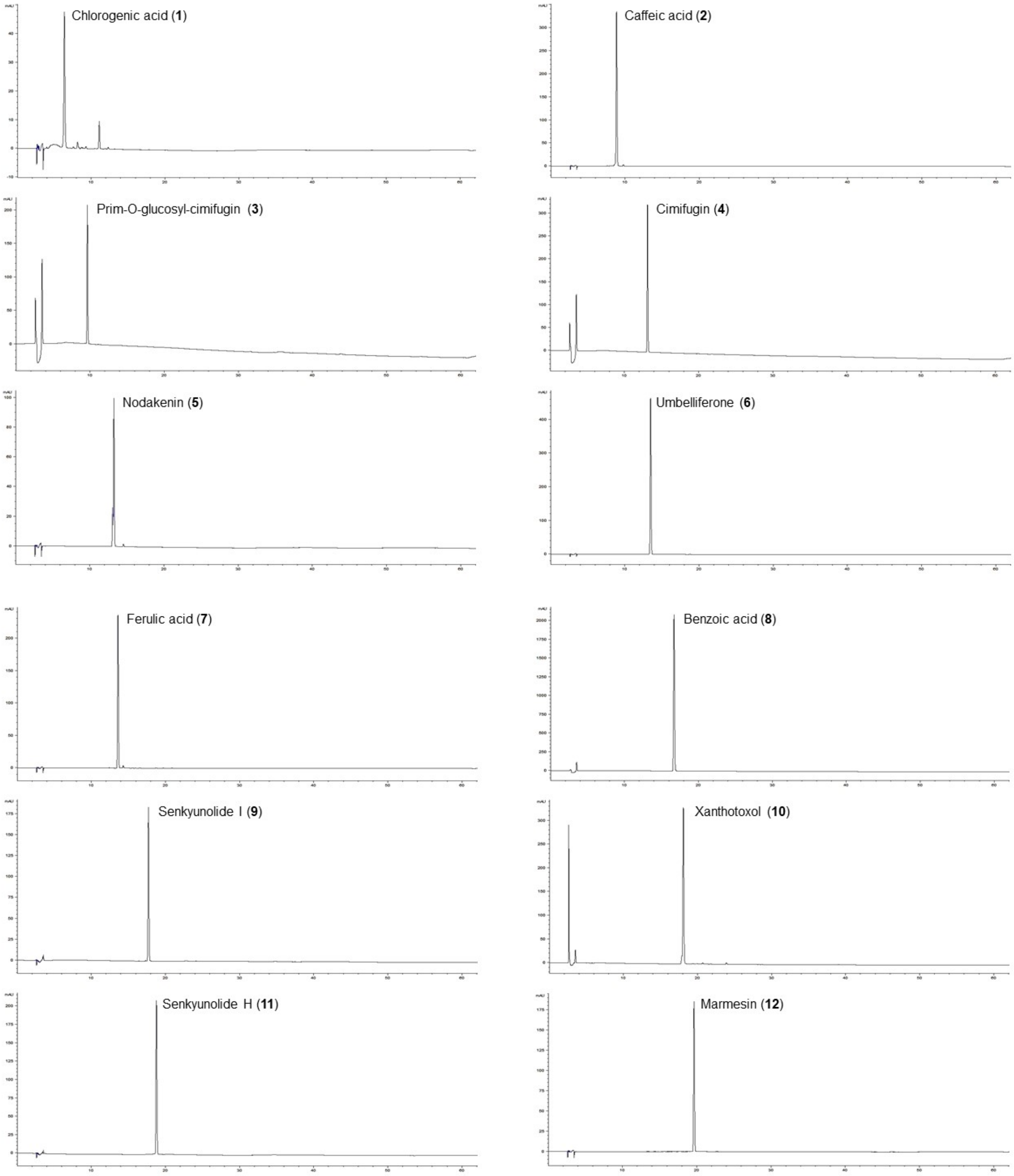

Figure S1. Single chromatograms of 46 marker compound.

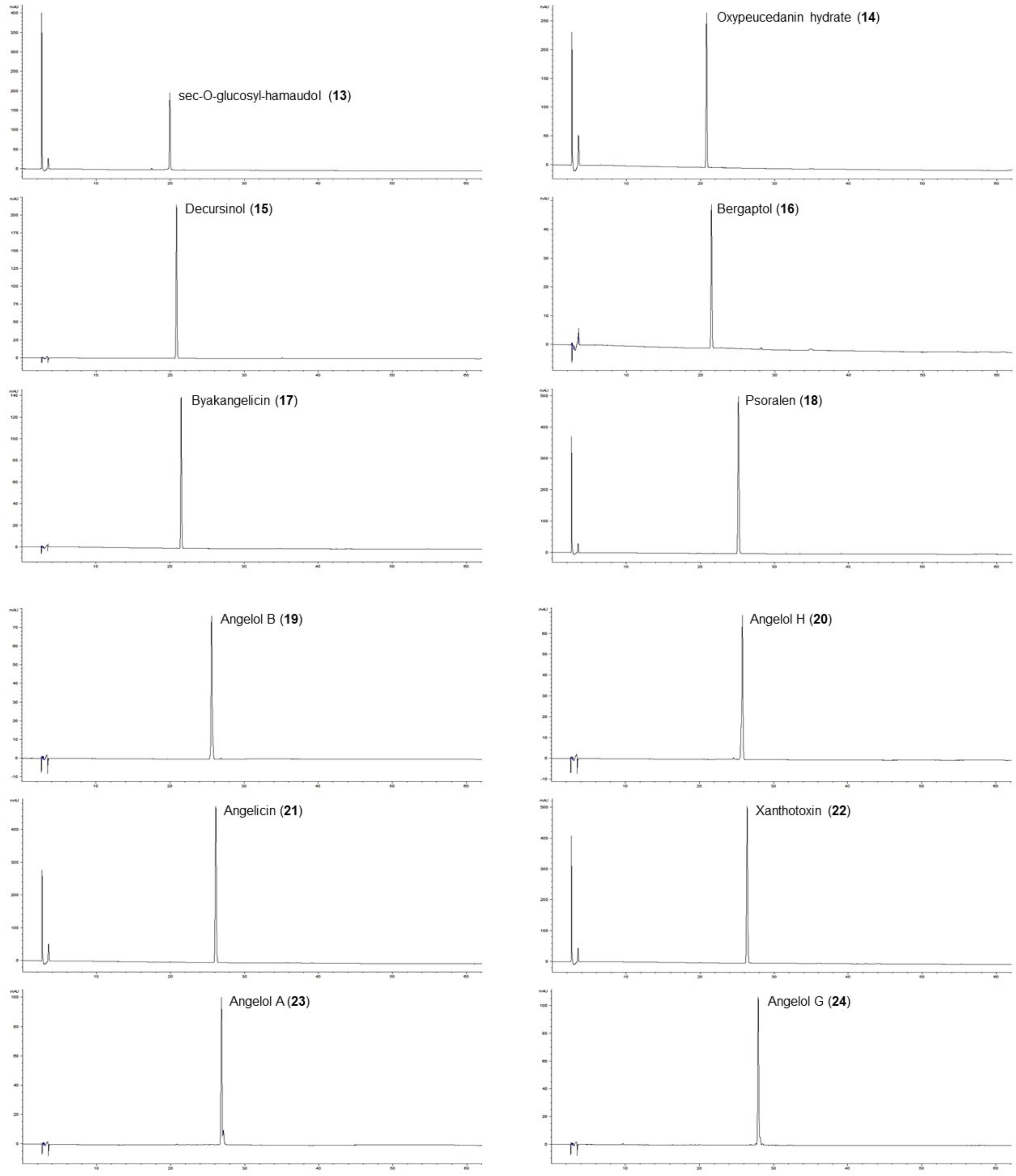
sec-O-glucosyl-hamaudol (13)
Oxypeucedanin hydrate (14)
Decursinol (15)
Bergaptol (16)
Byakangelicin (17)
Psoralen (18)
Angelol B (19)
Angelol H (20)
Angelicin (21)
Xanthotoxin (22)
Angelol A (23)
Angelol G (24)

Continued.

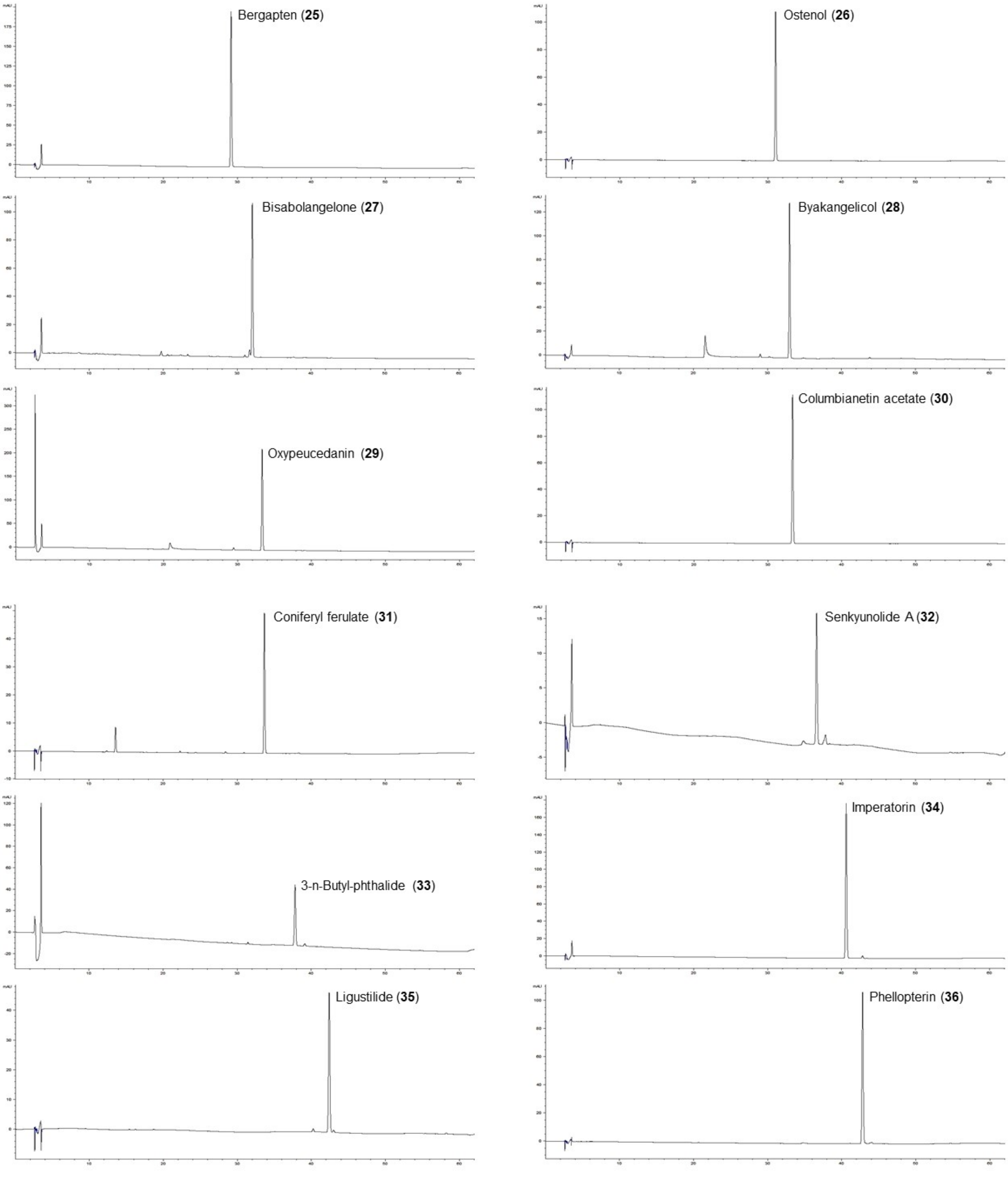
Bergapten (**25**)
Ostenol (**26**)
Bisabolangelone (**27**)
Byakangelicol (**28**)
Oxypeucedanin (**29**)
Columbianetin acetate (**30**)
Coniferyl ferulate (**31**)
Senkyunolide A (**32**)
3-n-Butyl-phthalide (**33**)
Imperatorin (**34**)
Ligustilide (**35**)
Phellopterin (**36**)

Continued.

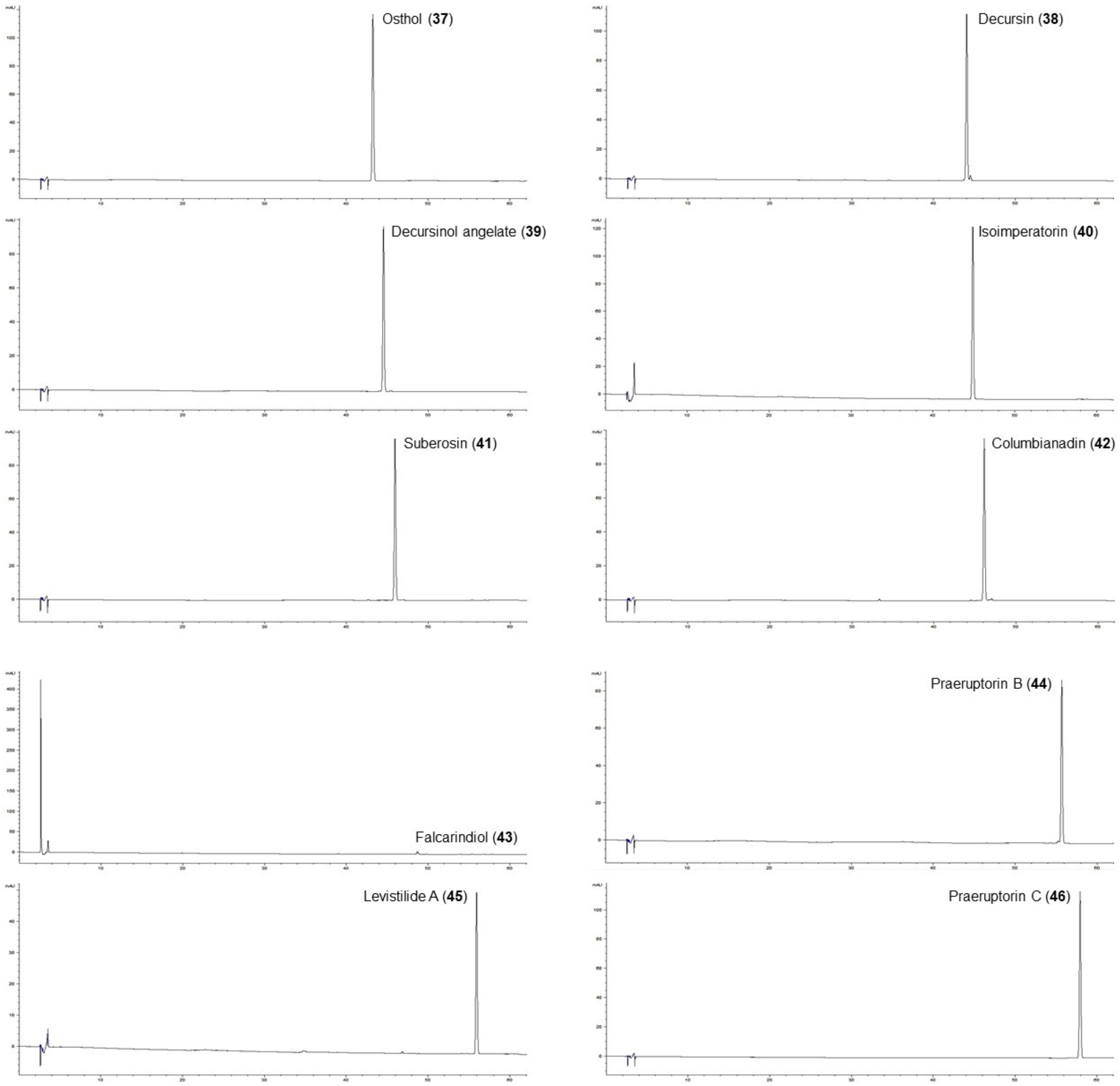
Osthol (37)
Decursin (38)
Decursinol angelate (39)
Isoimperatorin (40)
Suberosin (41)
Columbianadin (42)
Falcarindiol (43)
Praeruptorin B (44)
Levistilide A (45)
Praeruptorin C (46)

Continued.

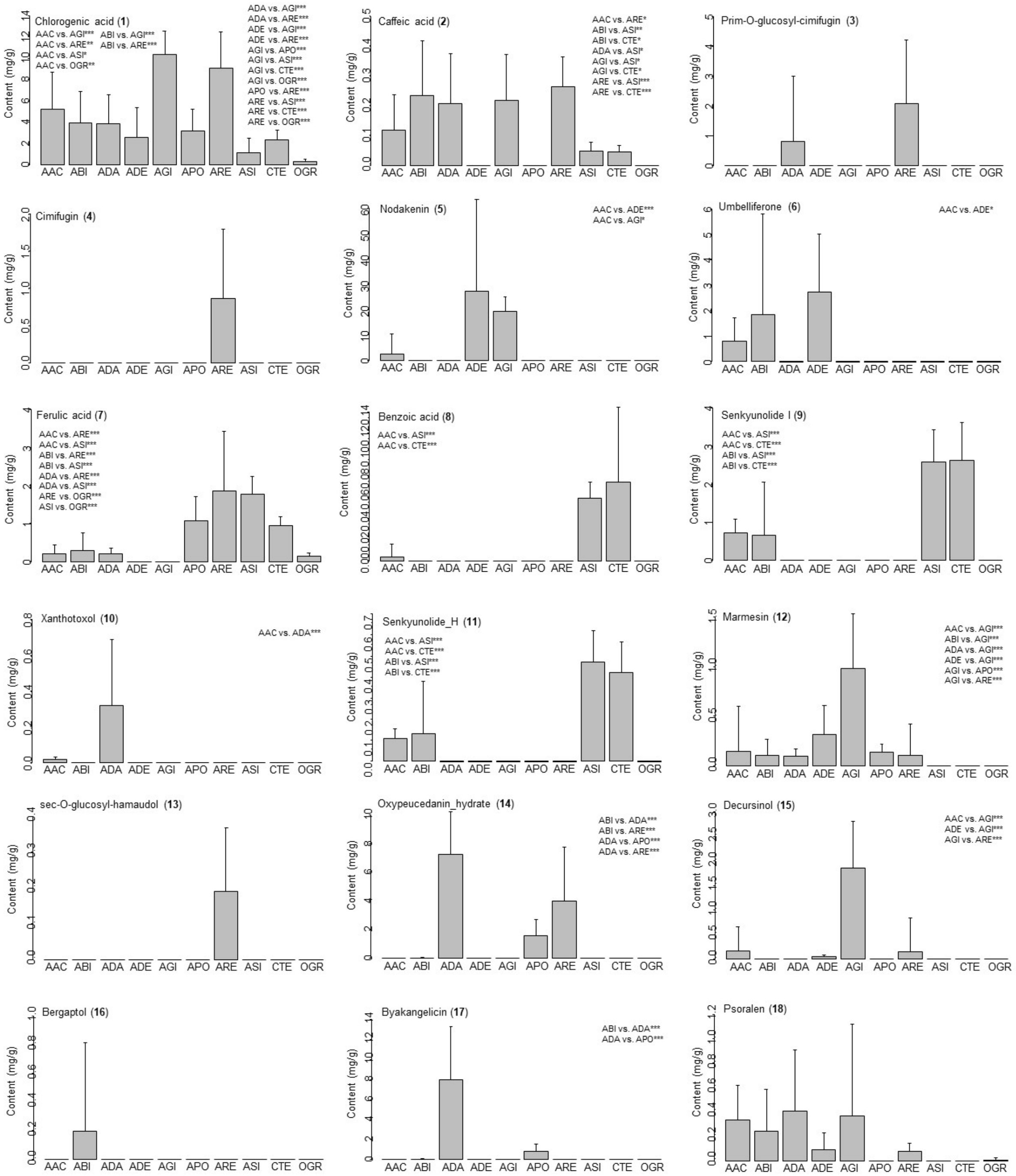

Figure S2. Comparison of the contents of the marker compounds among the samples. *Angelica acutiloba* (AAC), *A. biserrata* (ABI), *A. dahurica* (ADA), *A. decursiva* (ADE), *Angelica gigas* (AGI), *A. polymorpha* (APO), *A. reflexa* (ARE), *A. sinensis* (ASI), *Conioselinum tenuissimum* (CTE), and *Ostericum grosseratum* (OGR).

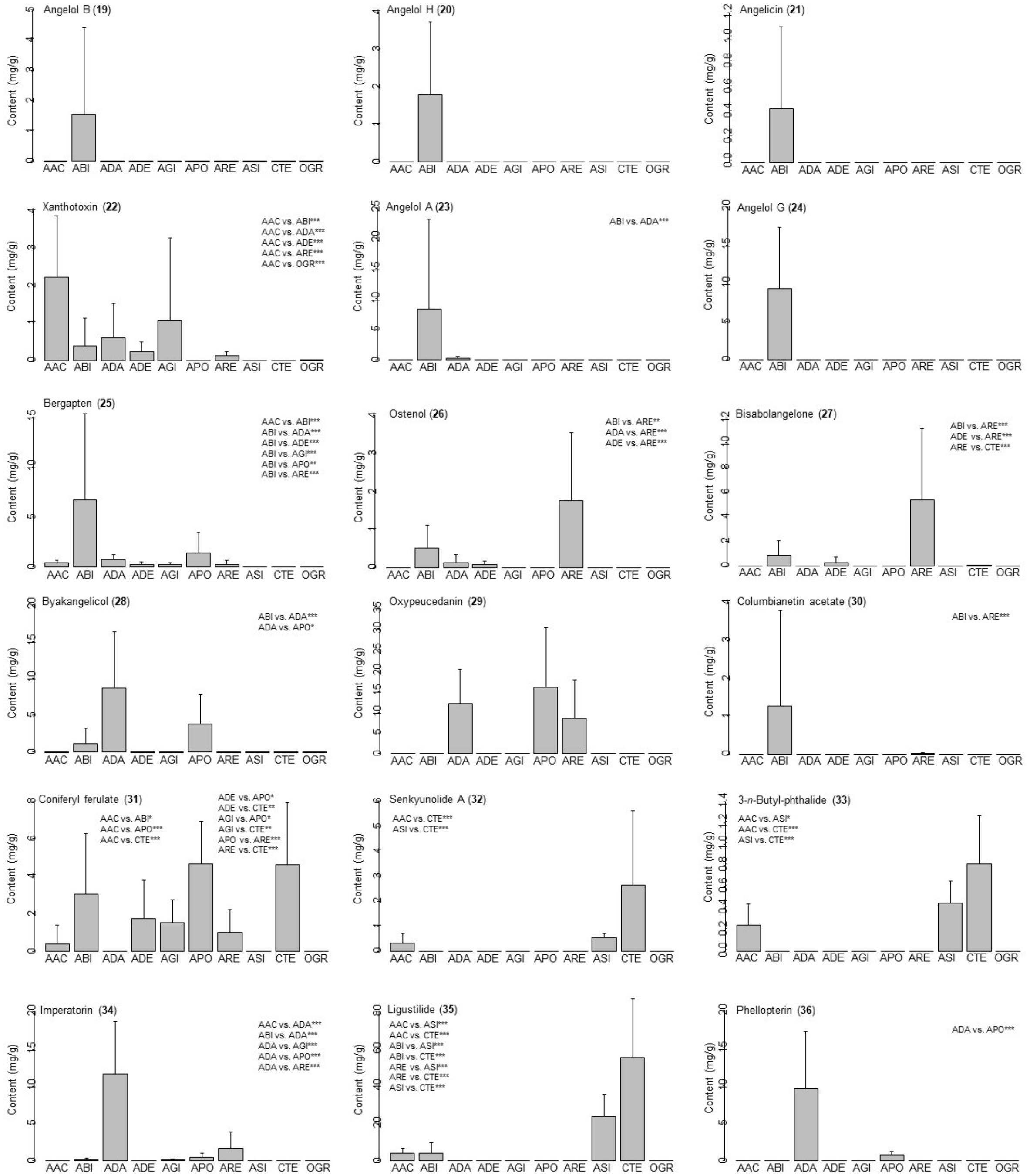

Angelol B (19)
Angelol H (20)
Angelicin (21)
Xanthotoxin (22)
AAC vs. ABI***
AAC vs. ADA***
AAC vs. ADE***
AAC vs. ARE***
AAC vs. OGR***
Angelol A (23)
ABI vs. ADA***
Angelol G (24)
Bergapten (25)
AAC vs. ABI***
ABI vs. ADA***
ABI vs. ADE***
ABI vs. AGI***
ABI vs. APO**
ABI vs. ARE***
Ostenol (26)
ABI vs. ARE**
ADA vs. ARE***
ADE vs. ARE***
Bisabolangelone (27)
ABI vs. ARE***
ADE vs. ARE***
ARE vs. CTE***
Byakangelicol (28)
ABI vs. ADA***
ADA vs. APO*
Oxypeucedanin (29)
Columbianetin acetate (30)
ABI vs. ARE***
Coniferyl ferulate (31)
AAC vs. ABI*
AAC vs. APO***
AAC vs. CTE***
ADE vs. APO*
ADE vs. CTE**
AGI vs. APO*
AGI vs. CTE**
APO vs. ARE***
ARE vs. CTE***
Senkyunolide A (32)
AAC vs. CTE***
ASI vs. CTE***
3-n-Butyl-phthalide (33)
AAC vs. ASI*
AAC vs. CTE***
ASI vs. CTE***
Imperatorin (34)
AAC vs. ADA***
ABI vs. ADA***
ADA vs. AGI***
ADA vs. APO***
ADA vs. ARE***
Ligustilide (35)
AAC vs. ASI***
AAC vs. CTE***
ABI vs. ASI***
ABI vs. CTE***
ARE vs. ASI***
ARE vs. CTE***
ASI vs. CTE***
Phellopterin (36)
ADA vs. APO***
Content (mg/g)
AAC ABI ADA ADE AGI APO ARE ASI CTE OGR

Continued.

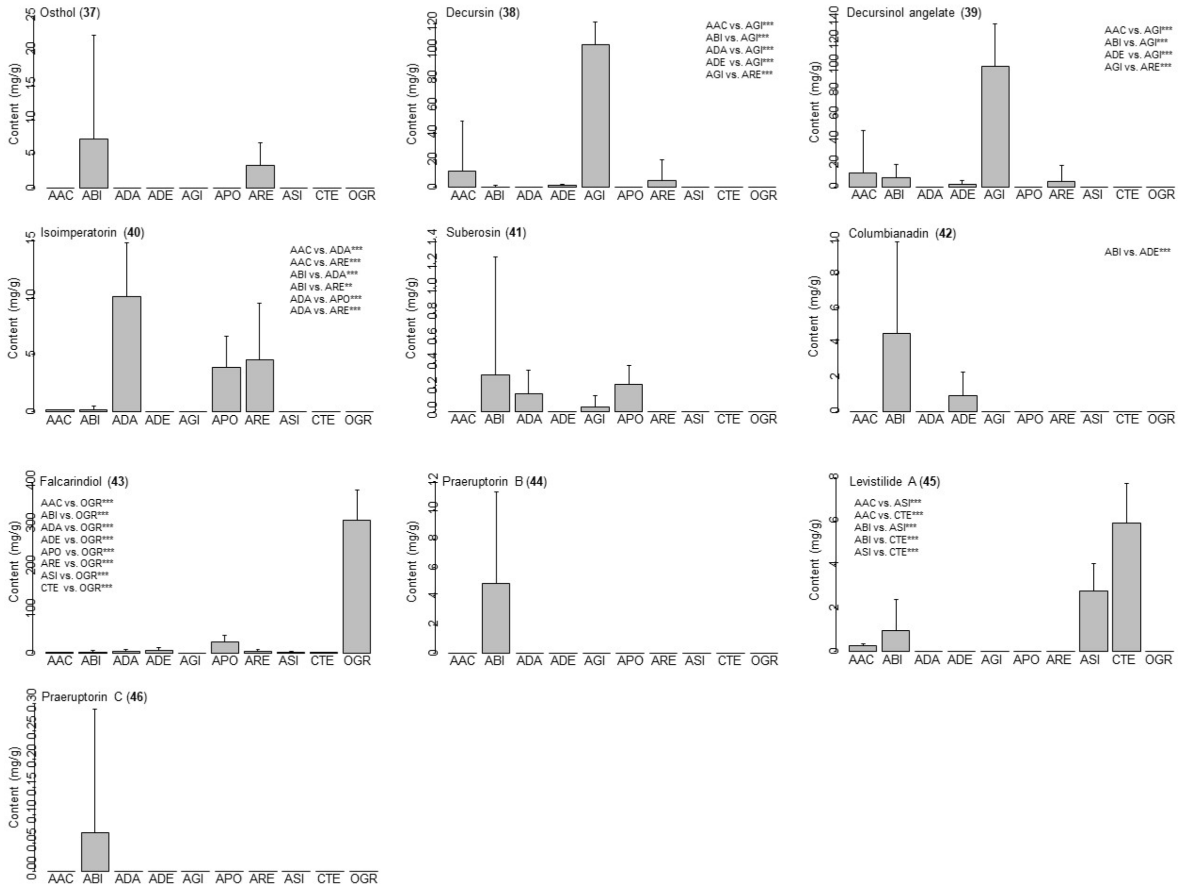
Osthol (37)
Decursin (38)
AAC vs. AGI***
ABI vs. AGI***
ADA vs. AGI***
ADE vs. AGI***
AGI vs. ARE***
Decursinol angelate (39)
AAC vs. AGI***
ABI vs. AGI***
ADE vs. AGI***
AGI vs. ARE***
Isoimperatorin (40)
AAC vs. ADA***
AAC vs. ARE***
ABI vs. ADA***
ABI vs. ARE**
ADA vs. APO***
ADA vs. ARE***
Suberosin (41)
Columbianadin (42)
ABI vs. ADE***
Falcarindiol (43)
AAC vs. OGR***
ABI vs. OGR***
ADA vs. OGR***
ADE vs. OGR***
APO vs. OGR***
ARE vs. OGR***
ASI vs. OGR***
CTE vs. OGR***
Praeruptorin B (44)
Levistilide A (45)
AAC vs. ASI***
AAC vs. CTE***
ABI vs. ASI***
ABI vs. CTE***
ASI vs. CTE***
Praeruptorin C (46)
Content (mg/g)
AAC ABI ADA ADE AGI APO ARE ASI CTE OGR

Continued.

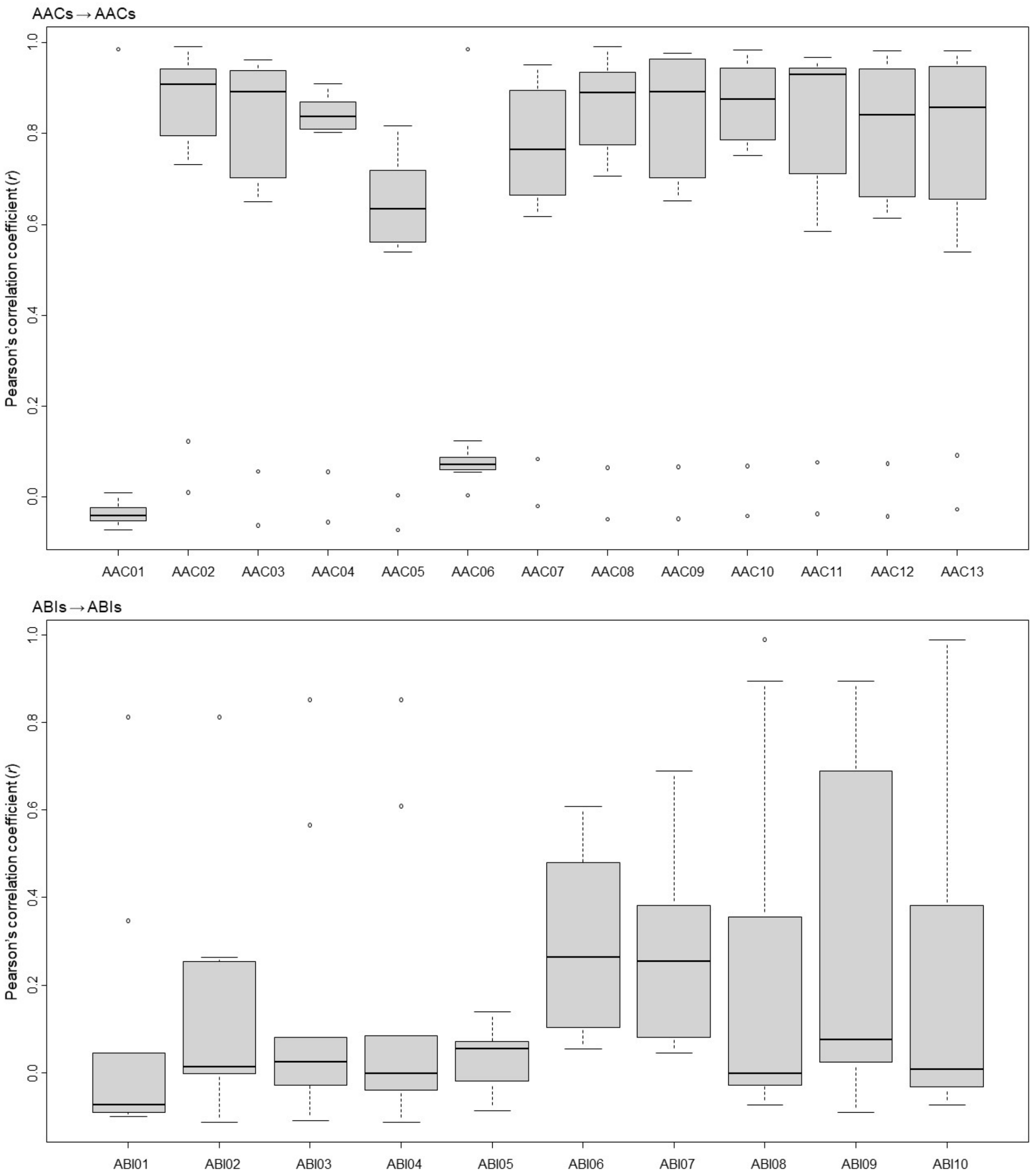

Figure S3. Intraspecies Pearson's correlation coefficients among the samples.

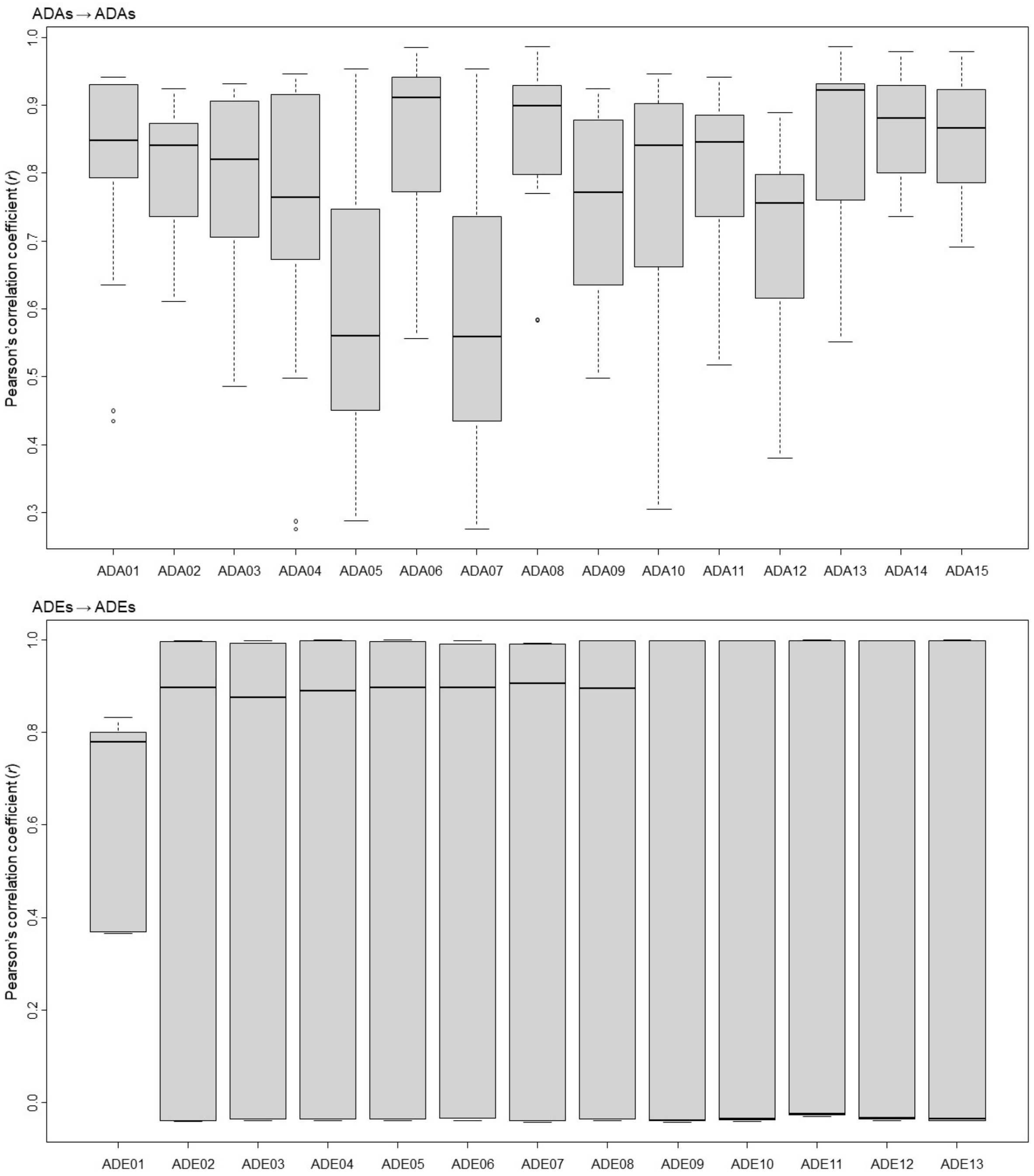

ADAs → ADAs
Pearson's correlation coefficient (r)
0.3
0.4
0.5
0.6
0.7
0.8
0.9
1.0
ADA01
ADA02
ADA03
ADA04
ADA05
ADA06
ADA07
ADA08
ADA09
ADA10
ADA11
ADA12
ADA13
ADA14
ADA15
ADEs → ADEs
Pearson's correlation coefficient (r)
0.0
0.2
0.4
0.6
0.8
1.0
ADE01
ADE02
ADE03
ADE04
ADE05
ADE06
ADE07
ADE08
ADE09
ADE10
ADE11
ADE12
ADE13

Continued.

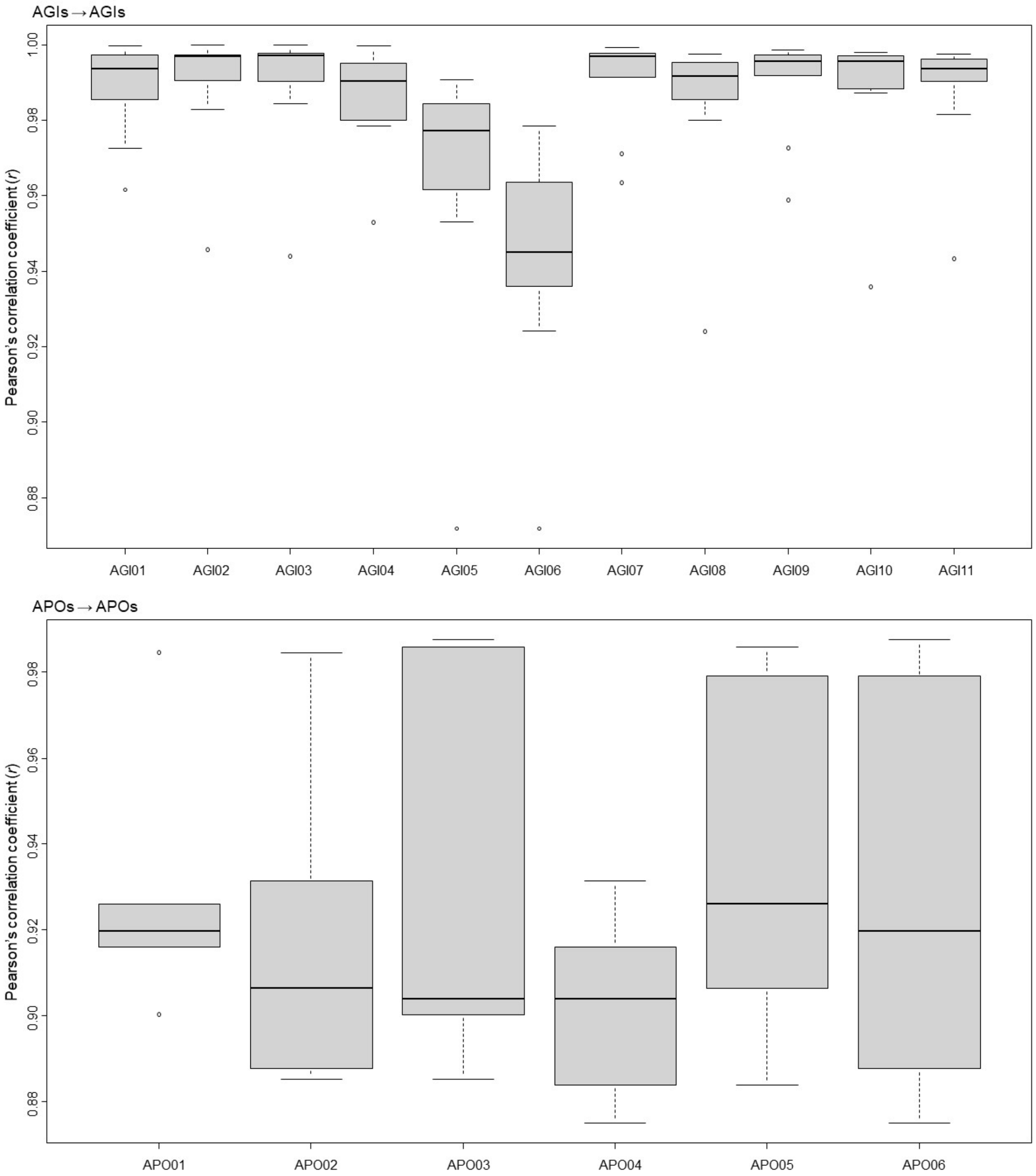
AGIs → AGIs
Pearson's correlation coefficient (r)
1.00
0.98
0.96
0.94
0.92
0.90
0.88
AGI01
AGI02
AGI03
AGI04
AGI05
AGI06
AGI07
AGI08
AGI09
AGI10
AGI11
APOs → APOs
Pearson's correlation coefficient (r)
0.98
0.96
0.94
0.92
0.90
0.88
APO01
APO02
APO03
APO04
APO05
APO06

Continued.

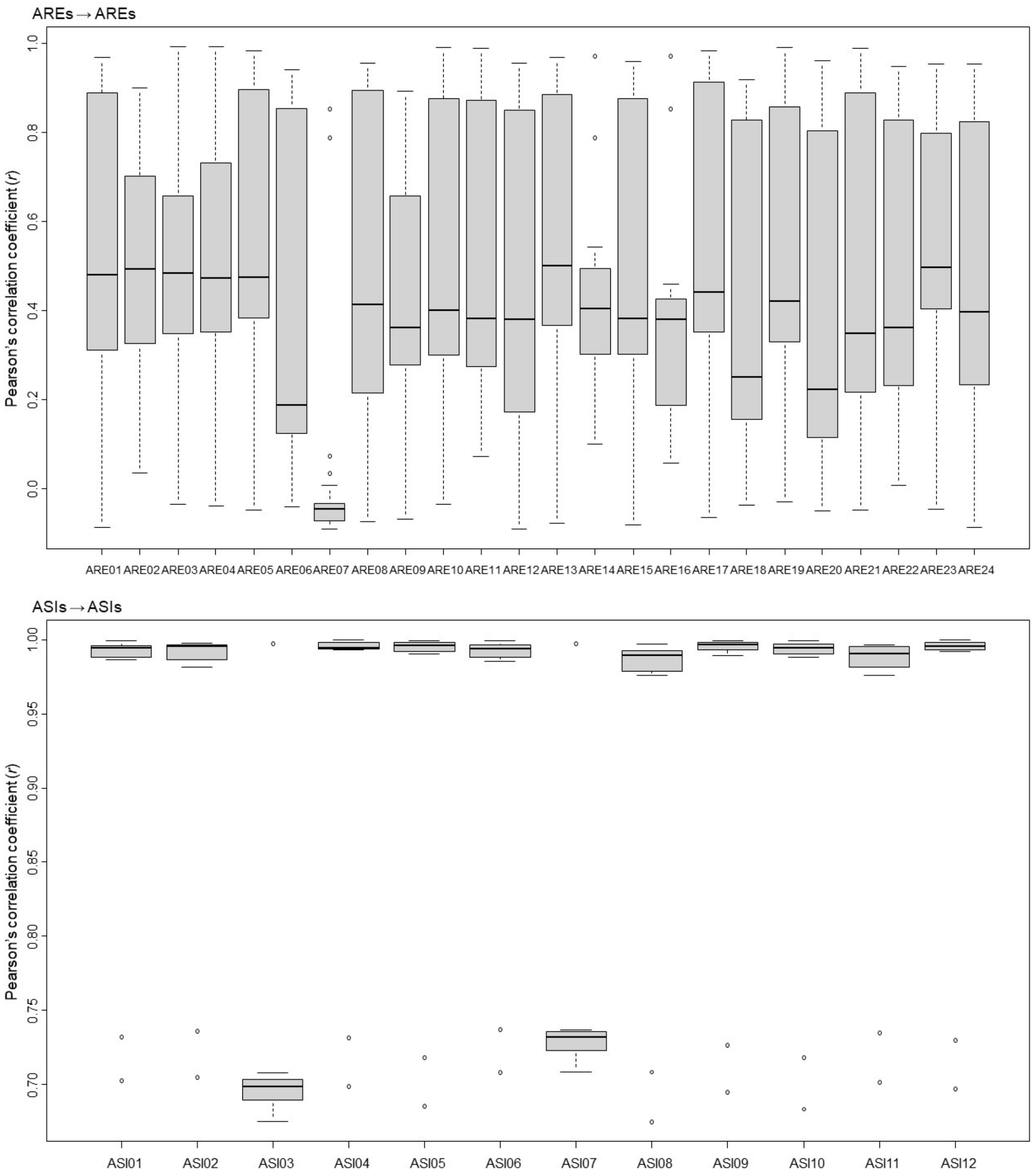
AREs → AREs
Pearson's correlation coefficient (r)
ARE01 ARE02 ARE03 ARE04 ARE05 ARE06 ARE07 ARE08 ARE09 ARE10 ARE11 ARE12 ARE13 ARE14 ARE15 ARE16 ARE17 ARE18 ARE19 ARE20 ARE21 ARE22 ARE23 ARE24
ASIs → ASIs
Pearson's correlation coefficient (r)
ASI01 ASI02 ASI03 ASI04 ASI05 ASI06 ASI07 ASI08 ASI09 ASI10 ASI11 ASI12

Continued.

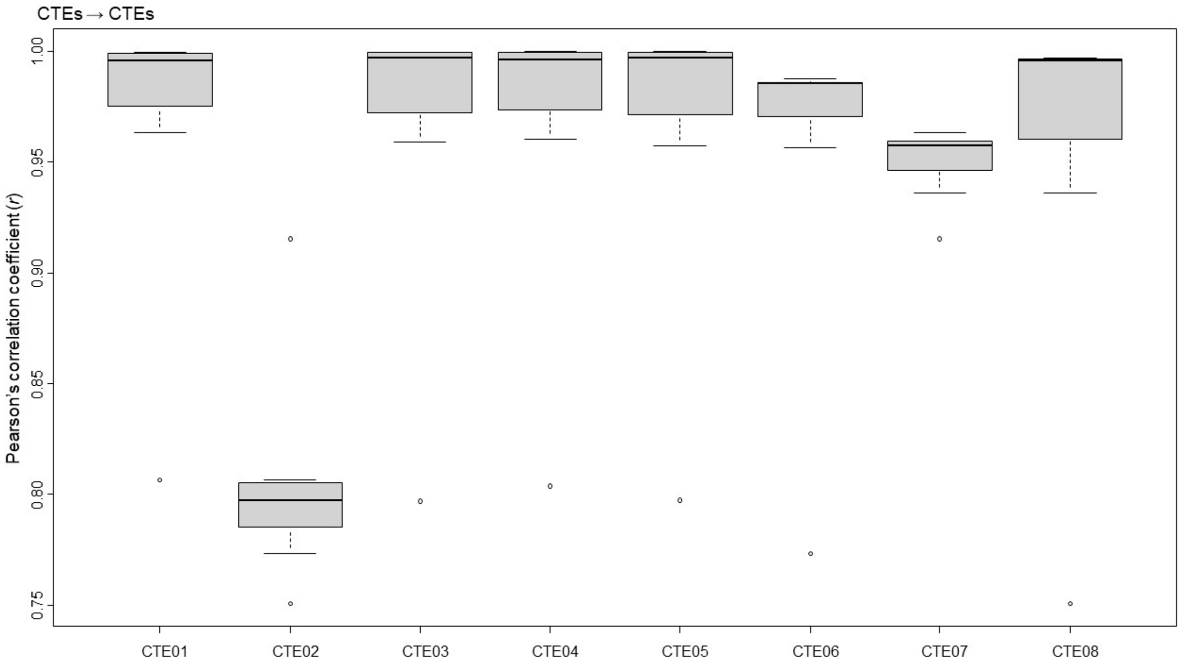
CTEs → CTEs
Pearson's correlation coefficient (r)
1.00
0.95
0.90
0.85
0.80
0.75
CTE01
CTE02
CTE03
CTE04
CTE05
CTE06
CTE07
CTE08

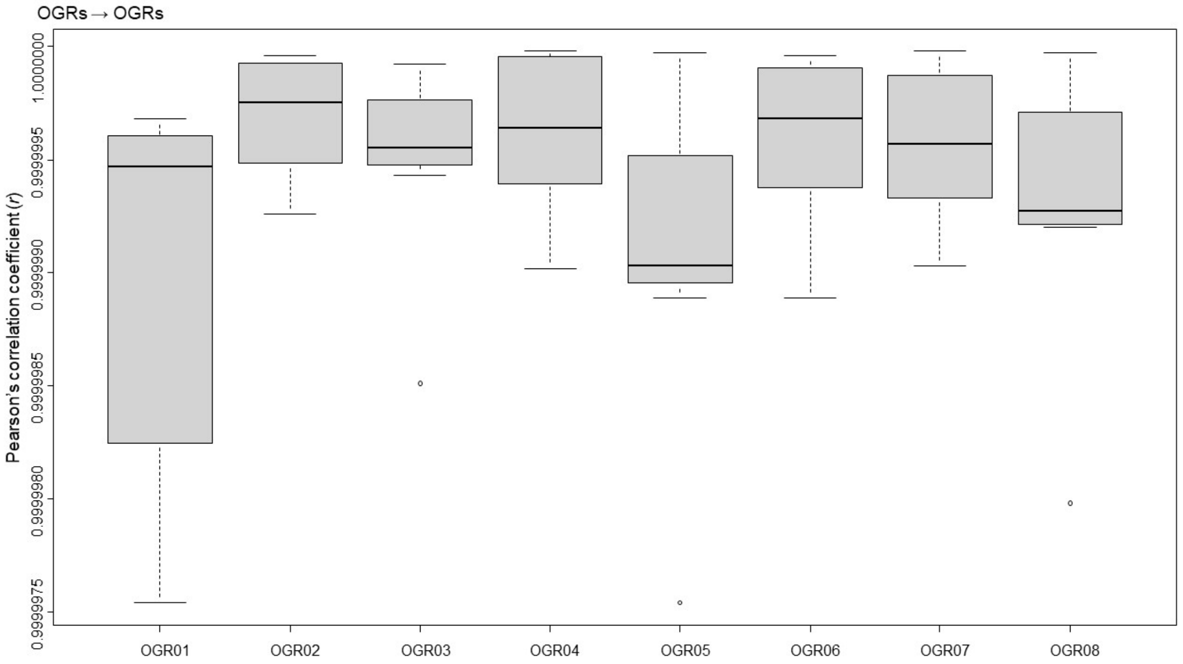
OGRs → OGRs
Pearson's correlation coefficient (r)
1.0000000
0.9999995
0.9999990
0.9999985
0.9999980
0.9999975
OGR01
OGR02
OGR03
OGR04
OGR05
OGR06
OGR07
OGR08

Continued.

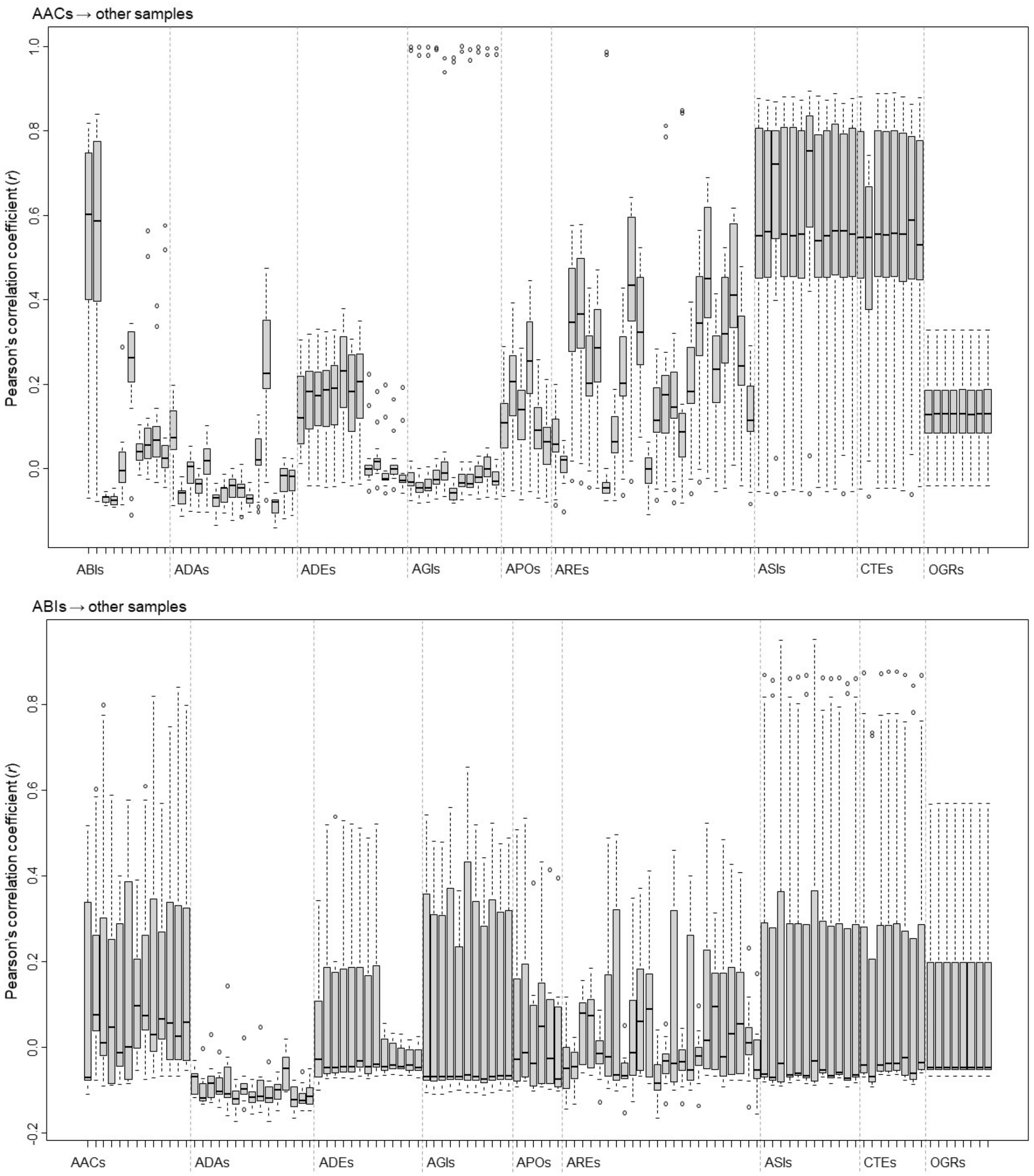

Figure S4. Interspecies Pearson's correlation coefficients among the samples.

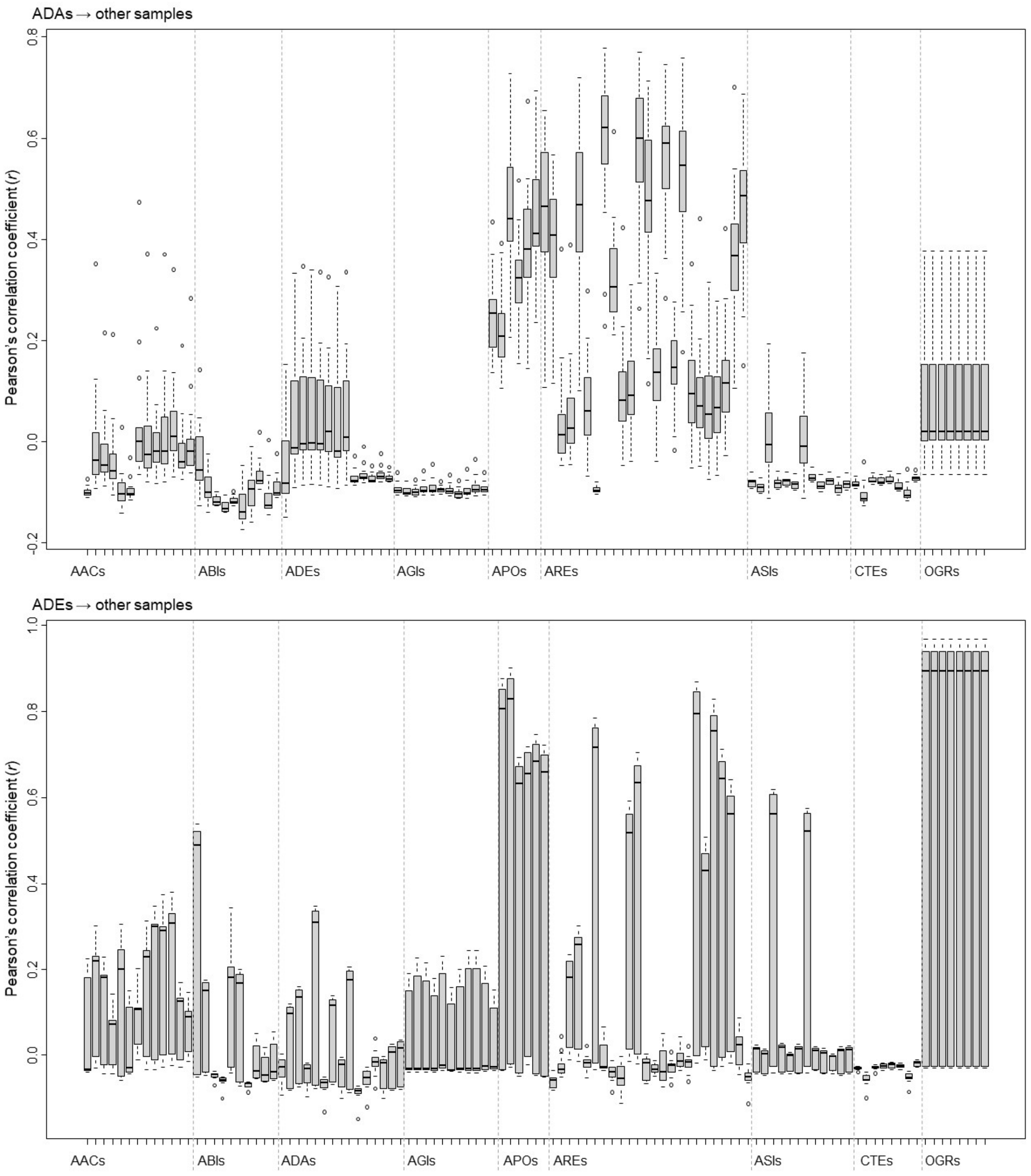

ADAs → other samples
Pearson's correlation coefficient (r)
AACs
ABIs
ADEs
AGIs
APOs
AREs
ASIs
CTEs
OGRs
ADEs → other samples
Pearson's correlation coefficient (r)
AACs
ABIs
ADAs
AGIs
APOs
AREs
ASIs
CTEs
OGRs

Continued.

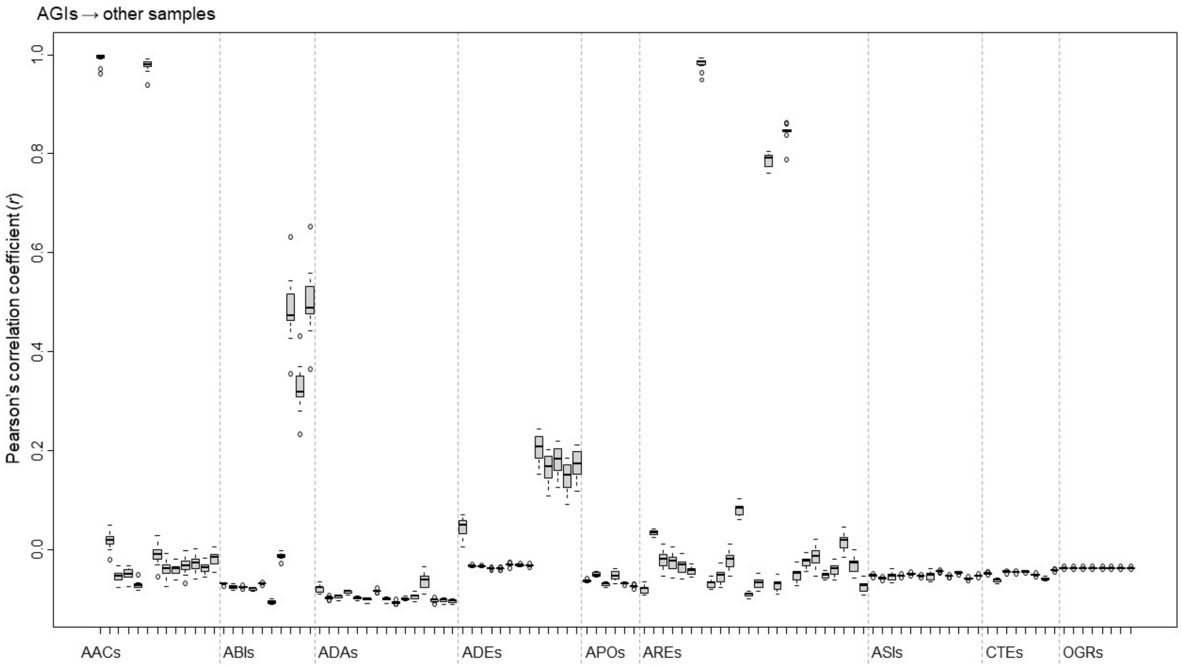

AGIs → other samples
Pearson's correlation coefficient (r)
1.0
0.8
0.6
0.4
0.2
0.0
AACs
ABIs
ADAs
ADEs
APOs
AREs
ASIs
CTEs
OGRs

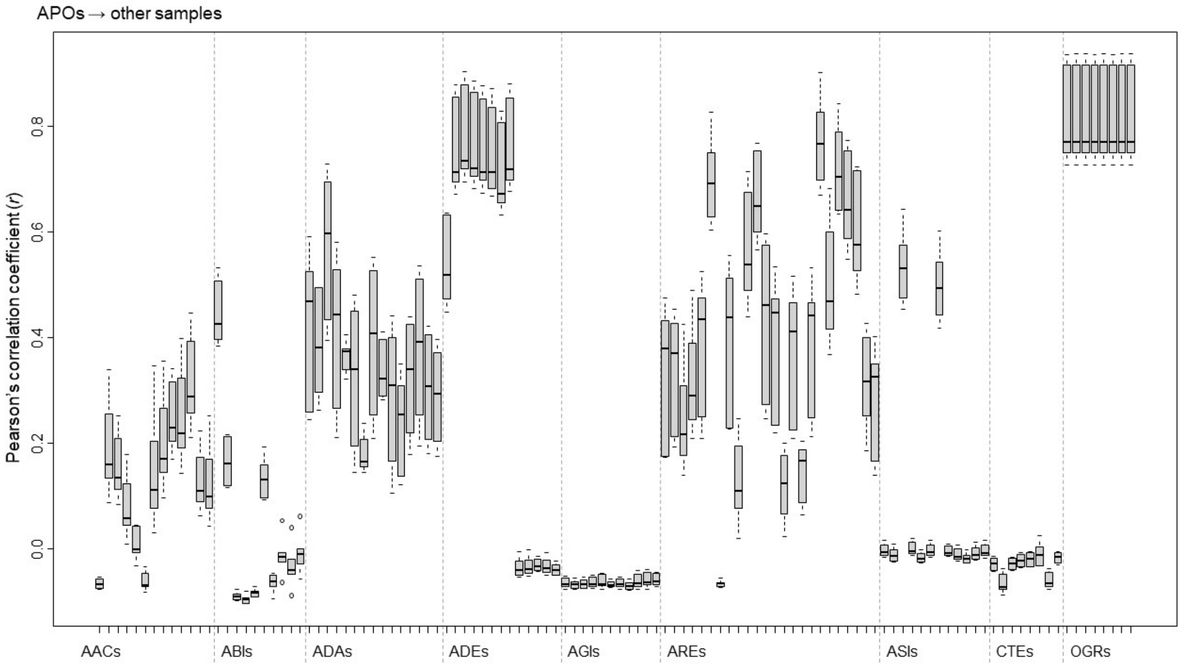

APOs → other samples
Pearson's correlation coefficient (r)
0.8
0.6
0.4
0.2
0.0
AACs
ABIs
ADAs
ADEs
AGIs
AREs
ASIs
CTEs
OGRs

Continued.

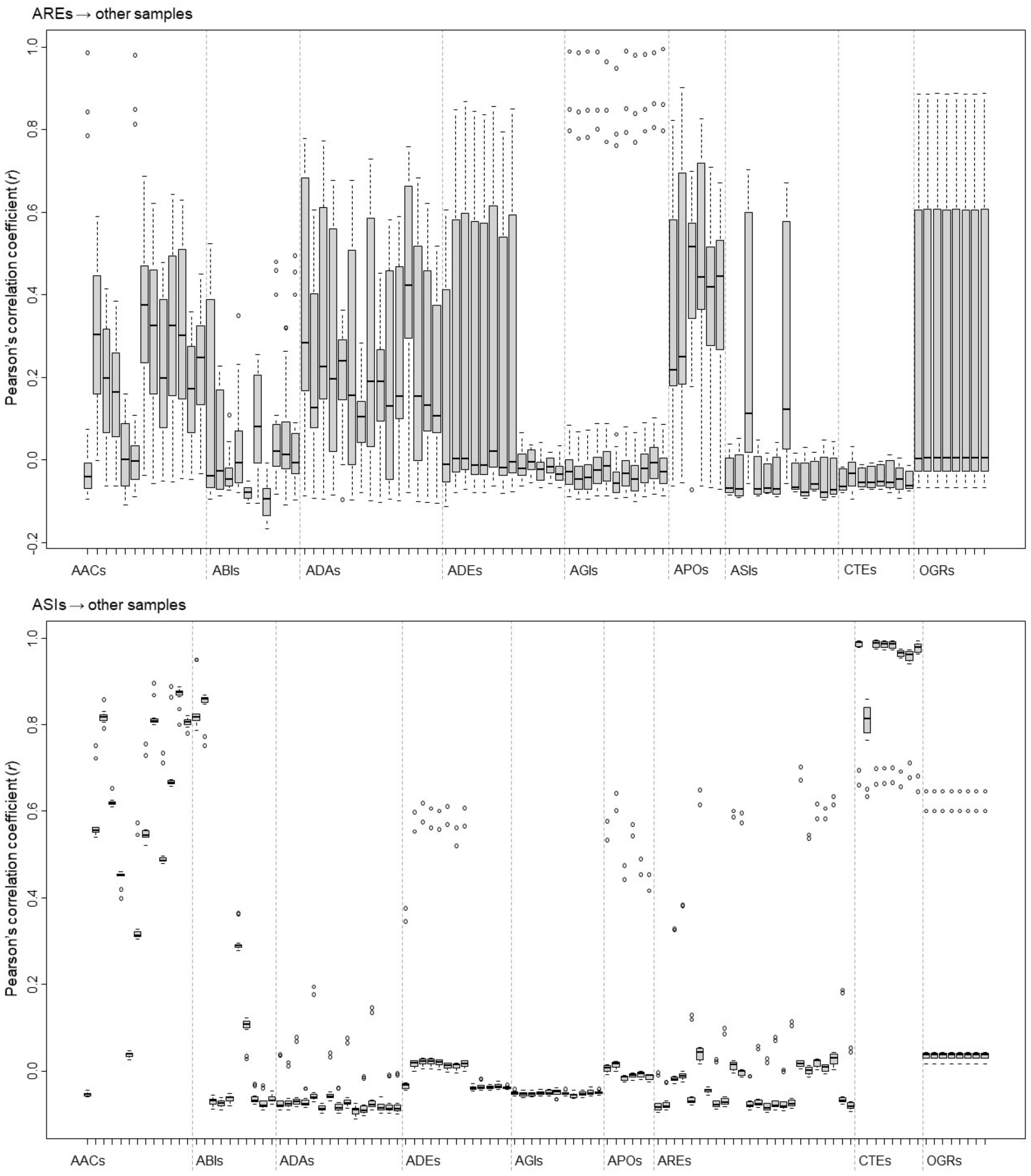
AREs → other samples
Pearson's correlation coefficient (r)
AACs
ABIs
ADAs
ADEs
AGIs
APOs
ASIs
CTEs
OGRs
ASIs → other samples
Pearson's correlation coefficient (r)
AACs
ABIs
ADAs
ADEs
AGIs
APOs
AREs
CTEs
OGRs

Continued.

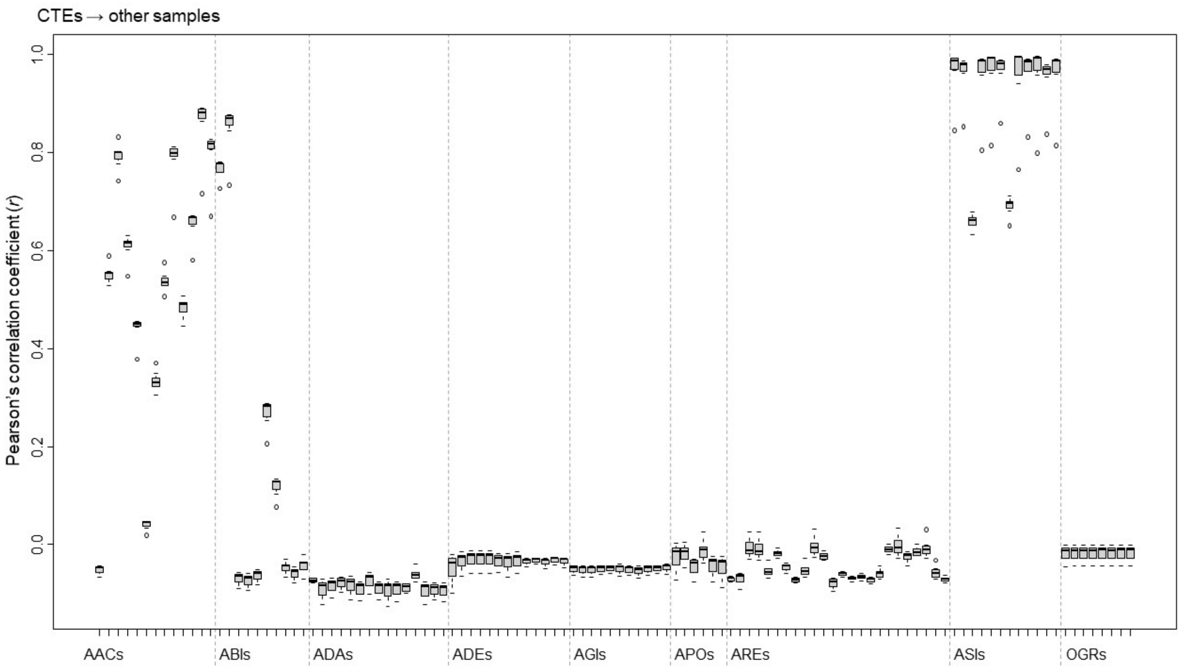

CTEs → other samples
Pearson's correlation coefficient (r)
1.0
0.8
0.6
0.4
0.2
0.0
AACs
ABIs
ADAs
ADEs
AGIs
APOs
AREs
ASIs
OGRs

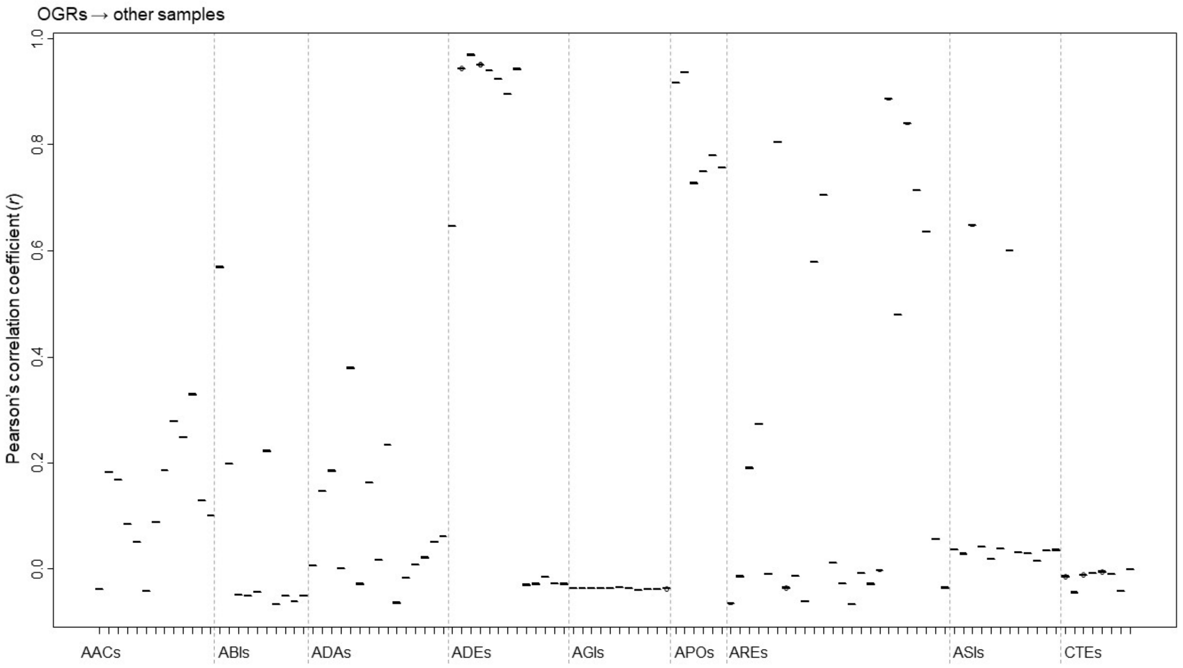

OGRs → other samples
Pearson's correlation coefficient (r)
1.0
0.8
0.6
0.4
0.2
0.0
AACs
ABIs
ADAs
ADEs
AGIs
APOs
AREs
ASIs
CTEs

Continued.

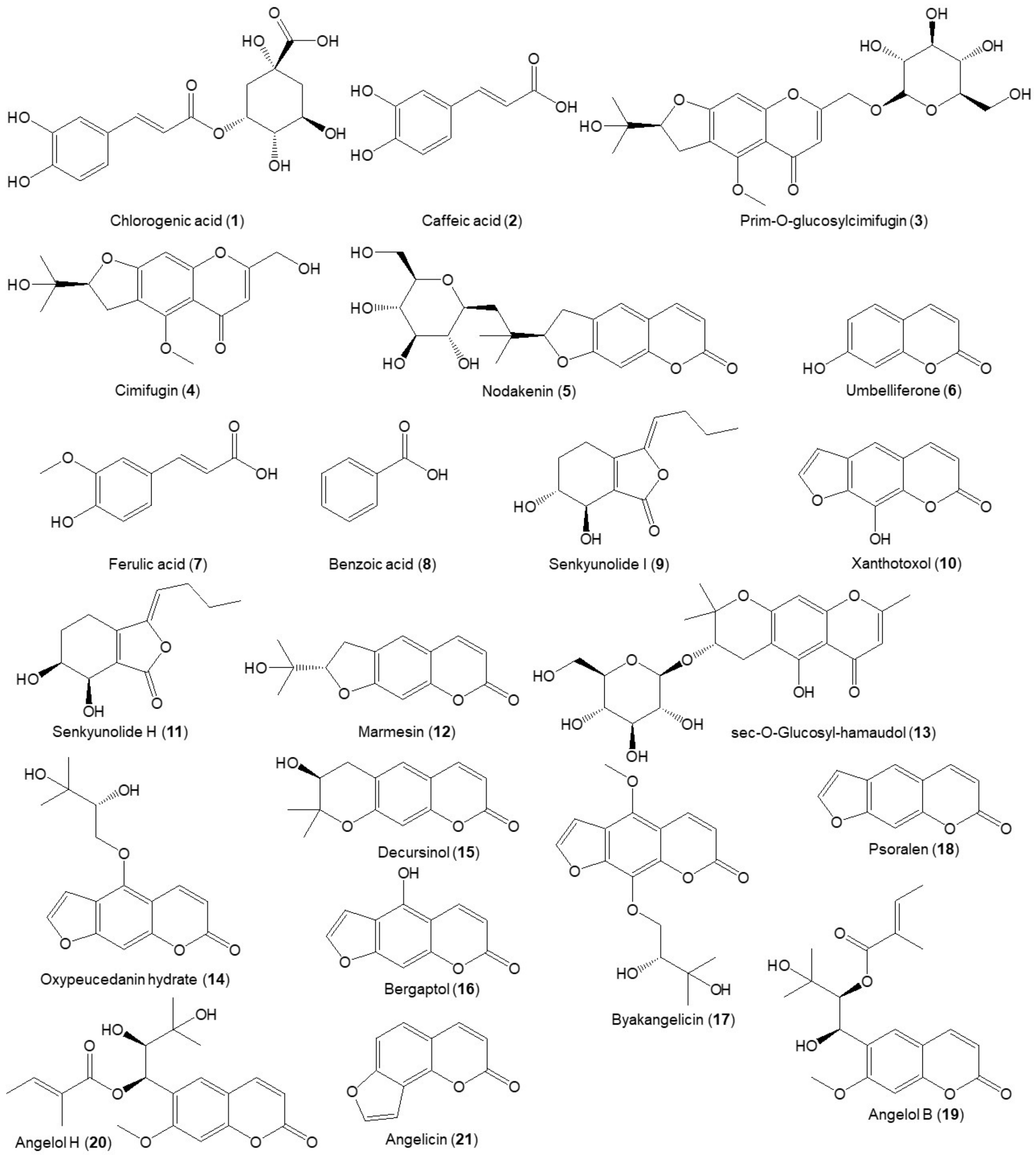

Figure S5. Chemical structures of 46 marker compounds.

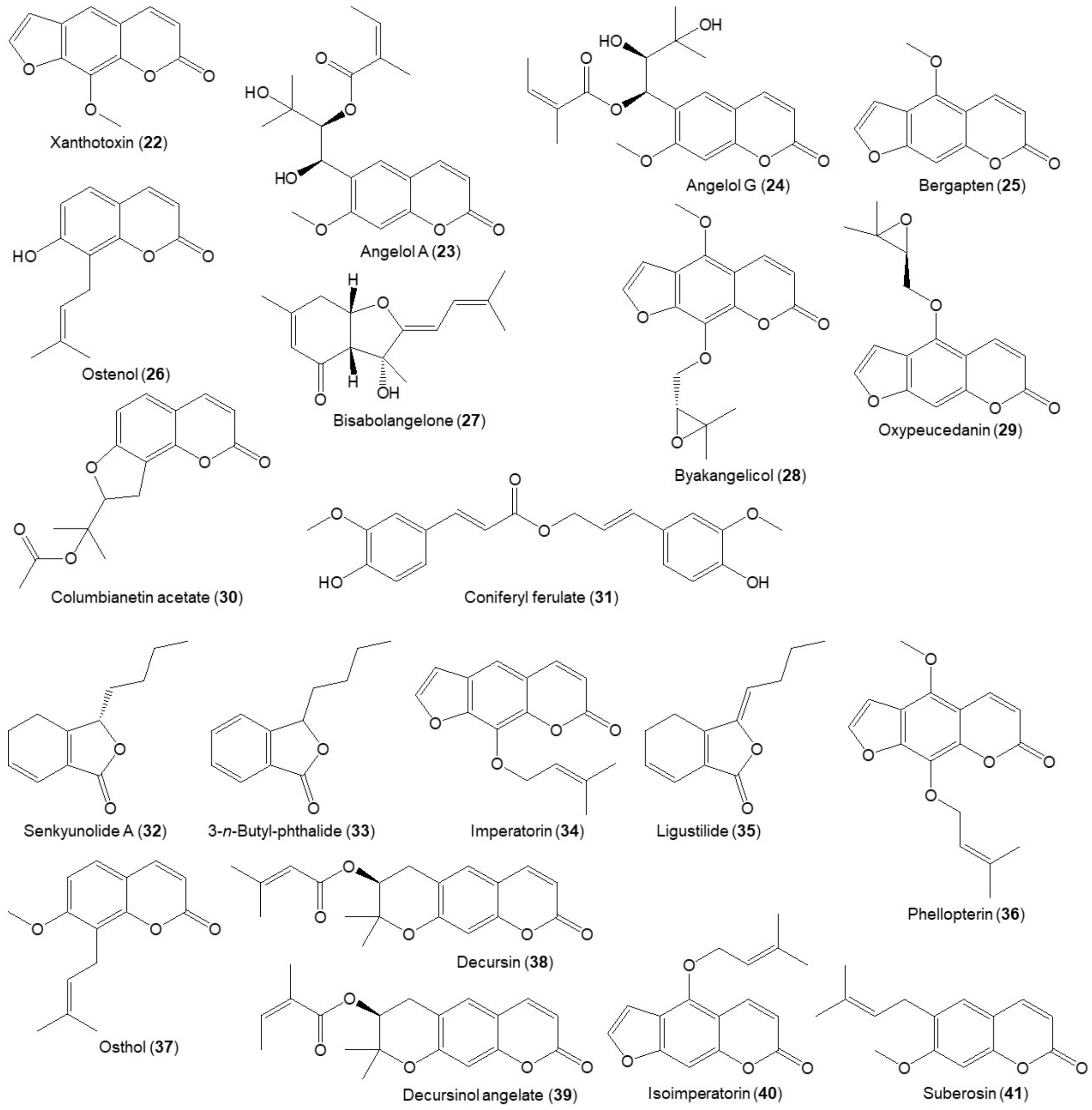
Xanthotoxin (22)
Angelol A (23)
Angelol G (24)
Bergapten (25)
Ostenol (26)
Bisabolangelone (27)
Byakangelicol (28)
Oxypeucedanin (29)
Columbianetin acetate (30)
Coniferyl ferulate (31)
Senkyunolide A (32)
3-n-Butyl-phthalide (33)
Imperatorin (34)
Ligustilide (35)
Phellopterin (36)
Osthol (37)
Decursin (38)
Decursinol angelate (39)
Isoimperatorin (40)
Suberosin (41)

Continued.

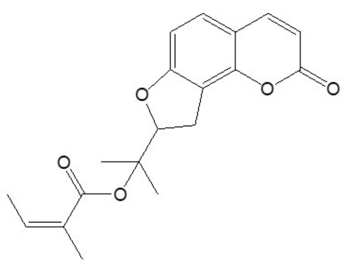

Columbianadin (**42**)

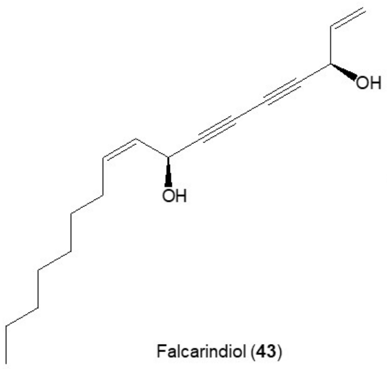

Falcarindiol (**43**)

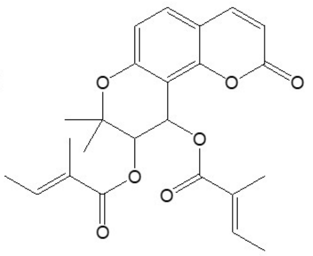

Praeruptorin B (**44**)

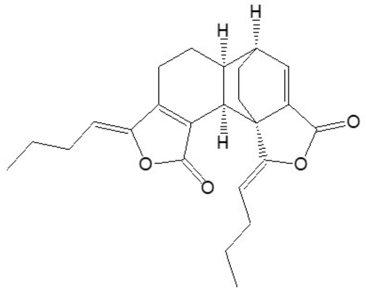

Levistilide A (**45**)

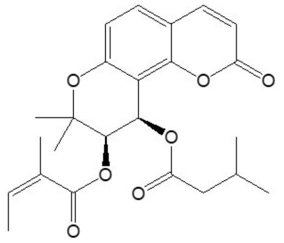

Praeruptorin C (**46**)

Continued.

Table S1. The sequence identity matrix and sequence difference count matrix of ITS nucleotide sequences in Table 1.

Matrix	Seq->	ABI	ADA	ASI	AGI	AAC	ADE	ARE	APO	CTE	OGR
Sequence identity matrix	ABI	ID	0.962	0.895	0.972	0.973	0.965	0.972	0.975	0.914	0.835
	ADA	0.962	ID	0.892	0.963	0.962	0.952	0.96	0.963	0.907	0.833
	ASI	0.895	0.892	ID	0.904	0.91	0.894	0.9	0.898	0.942	0.846
	AGI	0.972	0.963	0.904	ID	0.984	0.982	0.973	0.973	0.921	0.84
	AAC	0.973	0.962	0.91	0.984	ID	0.975	0.978	0.975	0.926	0.84
	ADE	0.965	0.952	0.894	0.982	0.975	ID	0.965	0.968	0.913	0.833
	ARE	0.972	0.96	0.9	0.973	0.978	0.965	ID	0.975	0.918	0.832
	APO	0.975	0.963	0.898	0.973	0.975	0.968	0.975	ID	0.911	0.833
	CTE	0.914	0.907	0.942	0.921	0.926	0.913	0.918	0.911	ID	0.853
	OGR	0.835	0.833	0.846	0.84	0.84	0.833	0.832	0.833	0.853	ID
Matrix	Seq->	ABI	ADA	ASI	AGI	AAC	ADE	ARE	APO	CTE	OGR
Sequence difference count matrix	ABI	ID	26	72	19	18	24	19	17	59	115
	ADA	26	ID	74	25	26	33	27	25	64	116
	ASI	72	74	ID	66	62	73	69	70	40	107
	AGI	19	25	66	ID	11	12	18	18	54	111
	AAC	18	26	62	11	ID	17	15	17	51	111
	ADE	24	33	73	12	17	ID	24	22	60	116
	ARE	19	27	69	18	15	24	ID	17	56	117
	APO	17	25	70	18	17	22	17	ID	61	116
	CTE	59	64	40	54	51	60	56	61	ID	102
	OGR	115	116	107	111	111	116	117	116	102	ID

Table S2. The sequence identity matrix and sequence difference count matrix of *psbA-trnH* nucleotide sequences in Table 1.

Matrix	Seq->	ABI	ARE	APO	ADA	AGI	AAC	ASI	CTE	ADE	OGR
Sequence Identity Matrix	ABI	ID	0.98	0.926	0.917	0.94	0.914	0.851	0.796	0.937	0.741
	ARE	0.98	ID	0.926	0.909	0.931	0.905	0.851	0.791	0.923	0.738
	APO	0.926	0.926	ID	0.961	0.955	0.935	0.882	0.821	0.952	0.761
	ADA	0.917	0.909	0.961	ID	0.975	0.954	0.888	0.832	0.967	0.765
	AGI	0.94	0.931	0.955	0.975	ID	0.972	0.906	0.841	0.99	0.779
	AAC	0.914	0.905	0.935	0.954	0.972	ID	0.92	0.852	0.963	0.789
	ASI	0.851	0.851	0.882	0.888	0.906	0.92	ID	0.917	0.909	0.847
	CTE	0.796	0.791	0.821	0.832	0.841	0.852	0.917	ID	0.843	0.876
	ADE	0.937	0.923	0.952	0.967	0.99	0.963	0.909	0.843	ID	0.784
	OGR	0.741	0.738	0.761	0.765	0.779	0.789	0.847	0.876	0.784	ID
Matrix	Seq->	ABI	ARE	APO	ADA	AGI	AAC	ASI	CTE	ADE	OGR
Sequence difference count Matrix	ABI	ID	7	26	29	21	30	52	73	22	94
	ARE	7	ID	26	32	24	33	52	75	27	95
	APO	26	26	ID	13	15	22	40	62	16	84
	ADA	29	32	13	ID	8	15	37	57	11	81
	AGI	21	24	15	8	ID	9	31	54	3	76
	AAC	30	33	22	15	9	ID	26	49	12	71
	ASI	52	52	40	37	31	26	ID	26	30	49
	CTE	73	75	62	57	54	49	26	ID	53	39
	ADE	22	27	16	11	3	12	30	53	ID	74
	OGR	94	95	84	81	76	71	49	39	74	ID

Table S3. Regression equation, linear range, correlation coefficient, limit of detection, and limit of quantification of the marker compounds

Compound	UV (nm)	t_R (min)	Regression equation	Linear range (μg/mL)	r^2	LOD (μg/mL)	LOQ (μg/mL)
Chlorogenic acid (**1**)	325nm	6.33	y = 18.9882x - 20.1736	4.69–300.00	1.0000	0.04	0.14
Caffeic acid (**2**)	325nm	8.83	y = 35.6640x - 3.0620	0.08–5.00	0.9992	0.02	0.07
Prim-O-glucosyl-cimifugin (**3**)	230nm	9.56	y = 18.3489x - 0.9000	1.56–100.00	1.0000	0.03	0.11
Cimifugin (**4**)	230nm	13.06	y = 26.7282x - 1.3057	0.78–50.00	1.0000	0.02	0.08
Nodakenin (**5**)	325nm	13.27	y = 18.8847x - 0.5929	0.78–50.00	1.0000	0.02	0.07
Umbelliferone (**6**)	325nm	13.44	y = 14.3679x - 2.3615	1.56–100.00	1.0000	0.01	0.03
Ferulic acid (**7**)	325nm	13.55	y = 39.8067x + 5.0408	0.70–45.00	1.0000	0.01	0.04
Benzoic acid (**8**)	230nm	16.64	y = 45.7381x + 0.3989	0.08–5.00	1.0000	0.01	0.03
Senkyunolide I (**9**)	280nm	17.67	y = 37.2178x + 3.1287	0.78–50.00	1.0000	0.02	0.05
Xanthotoxol (**10**)	250nm	18.05	y = 44.2476x + 0.0661	0.16–10.00	1.0000	0.01	0.04
Senkyunolide H (**11**)	280nm	18.74	y = 41.3349x + 0.9230	0.16–10.00	1.0000	0.02	0.05
Marmesin (**12**)	325nm	19.53	y = 35.2619x - 1.3776	0.78–50.00	1.0000	0.01	0.04
sec-O-Glucosyl-hamaudol (**13**)	250nm	19.85	y = 15.3224x - 0.0649	0.16–10.00	1.0000	0.02	0.06
Oxypeucedanin hydrate (**14**)	250nm	20.68	y = 34.9383x - 0.7443	2.34–150.00	1.0000	0.01	0.05
Decursinol (**15**)	325nm	20.77	y = 28.3230x - 0.2477	0.16–10.00	1.0000	0.01	0.05
Bergaptol (**16**)	270nm	21.46	y = 20.9488x + 1.4414	0.78–50.00	1.0000	0.02	0.07
Byakangelicin (**17**)	270nm	21.38	y = 28.2776x + 1.4902	3.13–200.00	1.0000	0.02	0.06
Psoralen (**18**)	250nm	25.07	y = 67.6458x - 0.3091	0.08–5.00	0.9999	0.01	0.03
Angelol B (**19**)	325nm	25.56	y = 15.2751x + 2.9632	1.56–100.00	0.9999	0.04	0.13
Angelol H (**20**)	325nm	25.72	y = 18.7174x + 1.5684	0.78–50.00	0.9999	0.03	0.10
Angelicin (**21**)	250nm	26.04	y = 62.3088x + 1.2799	0.16–10.00	1.0000	0.01	0.03
Xanthotoxin (**22**)	250nm	26.25	y = 55.4055x - 0.7218	0.16–10.00	1.0000	0.01	0.04
Angelol A (**23**)	325nm	26.72	y = 14.9625x + 0.1126	0.16–10.00	1.0000	0.03	0.11
Angelol G (**24**)	325nm	27.88	y = 20.3961x + 7.2678	2.34–150.00	0.9999	0.03	0.09
Bergapten (**25**)	250nm	29.07	y = 46.7556x - 1.1437	0.16–10.00	1.0000	0.01	0.05

Ostenol (**26**)	325nm	30.99	y = 21.7660x + 0.1259	0.16–10.00	1.0000	0.02	0.06
Bisabolangelone (**27**)	250nm	31.99	y = 22.5984x + 0.2770	1.56–100.00	1.0000	0.02	0.08
Byakangelicol (**28**)	270nm	32.84	y = 19.7989x - 0.0385	0.31–20.00	1.0000	0.03	0.10
Oxypeucedanin (**29**)	250nm	33.16	y = 27.5815x + 1.9420	1.56–100.00	1.0000	0.02	0.08
Columbianetin acetate (**30**)	325nm	33.28	y = 25.8919x + 7.4690	1.56–100.00	0.9999	0.02	0.08
Coniferyl ferulate (**31**)	325nm	33.58	y = 5.6940x - 2.5937	0.78–50.00	1.0000	0.11	0.37
Senkyunolide A (**32**)	280nm	36.53	y = 5.4381x + 0.7766	0.47–30.00	0.9999	0.08	0.26
3-*n*-Butyl-phthalide (**33**)	230nm	37.72	y = 22.6709x - 0.4780	0.16–10.00	1.0000	0.03	0.09
Imperatorin (**34**)	250nm	40.49	y = 49.3342x + 4.3161	0.08–5.00	1.0000	0.01	0.03
Ligustilide (**35**)	325nm	42.41	y = 12.6081x + 7.9914	4.69–300.00	1.0000	0.06	0.20
Phellopterin (**36**)	270nm	42.68	y = 31.7581x + 1.6885	3.13–200.00	1.0000	0.02	0.07
Osthol (**37**)	325nm	43.19	y = 31.7406x + 24.8006	3.91–250.00	0.9999	0.02	0.08
Decursin (**38**)	325nm	43.94	y = 30.1186x - 3.7925	6.25–400.00	1.0000	0.02	0.06
Decursinol angelate (**39**)	325nm	44.40	y = 19.4719x - 3.6483	6.25–400.00	1.0000	0.03	0.09
Isoimperatorin (**40**)	250nm	44.73	y = 33.7227x + 2.9736	2.34–150.00	1.0000	0.02	0.07
Suberosin (**41**)	325nm	45.75	y = 26.4464x - 0.2940	0.08–5.00	1.0000	0.02	0.07
Columbianadin (**42**)	325nm	46.02	y = 25.1184x + 6.8247	1.56–100.00	0.9999	0.03	0.09
Falcarindiol (**43**)	250nm	48.58	y = 0.9440x + 0.0718	2.34–150.00	1.0000	0.62	2.07
Praeruptorin B (**44**)	325nm	55.60	y = 17.2871x + 2.1391	1.25–80.00	1.0000	0.04	0.14
Levistilide A (**45**)	280nm	55.79	y = 13.5757x + 1.0816	1.56–100.00	1.0000	0.06	0.21
Praeruptorin C (**46**)	325nm	57.84	y = 17.1114x + 0.4494	0.16–10.00	0.9999	0.04	0.14

*t*R, retention time; UV, detection wavelength; r^2, correlation coefficient; LOD, limit of detection; LOQ, limit of quantification.

Table S4. Intra- and interday precisions of the marker compounds

Compound	Initial conc. (μg/mL)	Intraday (n = 3)			Interday (n = 3)		
		Detected conc. (μg/mL)	RSD (%)	Accuracy (%)	Detected conc. (μg/mL)	RSD (%)	Accuracy (%)
Chlorogenic acid (**1**)	37.50	36.76	0.20	98.04	36.98	0.95	98.60
	150.00	145.73	0.07	97.15	146.87	1.38	97.91
Caffeic acid (**2**)	0.63	0.58	0.84	92.25	0.58	1.11	93.29
	2.50	2.42	0.48	96.60	2.43	0.87	97.35
Prim-O-glucosyl-cimifugin (**3**)	12.50	12.53	0.40	100.21	12.53	0.31	100.26
	50.00	50.57	1.60	101.14	50.56	1.62	101.13
Cimifugin (**4**)	6.25	6.29	0.30	100.57	6.28	0.31	100.53
	25.00	25.28	1.43	101.13	25.26	1.57	101.05
Nodakenin (**5**)	6.25	6.23	0.54	99.66	6.26	0.39	100.20
	25.00	24.42	0.09	97.66	24.57	1.01	98.28
Umbelliferone (**6**)	12.50	12.61	0.25	100.89	12.59	0.58	100.68
	50.00	50.48	1.36	100.95	50.43	1.53	100.85
Ferulic acid (**7**)	5.63	5.66	0.09	100.65	5.67	0.21	100.72
	22.50	22.06	0.22	98.05	22.21	1.40	98.73
Benzoic acid (**8**)	0.63	0.63	0.72	101.22	0.63	1.03	101.45
	2.50	2.48	0.84	99.03	2.49	0.46	99.52
Senkyunolide I (**9**)	6.25	6.29	0.07	100.66	6.29	0.09	100.68
	25.00	24.51	0.20	98.03	24.64	1.16	98.57
Xanthotoxol (**10**)	1.25	1.27	0.78	101.49	1.26	1.17	101.13
	5.00	5.03	0.82	100.68	5.03	0.84	100.66
Senkyunolide H (**11**)	1.25	1.26	0.22	100.66	1.26	0.22	100.66
	5.00	4.92	0.16	98.32	4.94	1.14	98.89
Marmesin (**12**)	6.25	6.30	0.12	100.72	6.29	0.20	100.60
	25.00	24.49	0.01	97.95	24.60	0.84	98.42

sec-O-Glucosyl-hamaudol (**13**)	1.25	1.24	0.30	98.84	1.24	1.09	99.37
	5.00	4.97	1.25	99.42	4.99	0.69	99.81
Oxypeucedanin hydrate (**14**)	18.75	18.95	0.55	101.07	18.85	1.22	100.54
	75.00	75.57	0.81	100.77	75.62	0.71	100.83
Decursinol (**15**)	1.25	1.26	0.32	100.50	1.26	0.32	100.50
	5.00	4.90	0.04	98.00	4.92	0.83	98.45
Bergaptol (**16**)	6.25	6.22	0.12	99.51	6.24	0.69	99.84
	25.00	24.85	0.12	99.42	24.92	0.58	99.69
Byakangelicin (**17**)	25.00	25.31	0.52	101.26	25.18	1.18	100.73
	100.00	100.69	0.80	100.69	100.75	0.68	100.75
Psoralen (**18**)	0.63	0.63	0.36	100.39	0.63	0.36	100.39
	2.50	2.45	0.14	98.12	2.46	0.80	98.50
Angelol B (**19**)	12.50	12.66	0.06	101.27	12.66	0.09	101.26
	50.00	50.24	0.08	100.49	50.30	0.26	100.60
Angelol H (**20**)	6.25	6.36	0.13	101.72	6.36	0.21	101.81
	25.00	25.12	0.26	100.48	25.14	0.15	100.58
Angelicin (**21**)	1.25	1.26	0.07	100.77	1.26	0.22	100.69
	5.00	5.05	0.05	100.91	5.04	0.16	100.85
Xanthotoxin (**22**)	1.25	1.26	0.08	100.77	1.26	0.14	100.67
	5.00	4.90	0.11	98.10	4.93	0.70	98.55
Angelol A (**23**)	1.25	1.27	1.05	101.52	1.26	1.40	100.99
	5.00	5.04	0.69	100.77	5.04	0.61	100.81
Angelol G (**24**)	18.75	18.93	0.26	100.94	18.96	0.15	101.12
	75.00	75.30	0.06	100.40	75.40	0.27	100.54
Bergapten (**25**)	1.25	1.25	0.26	100.40	1.25	0.17	100.17
	5.00	4.90	0.04	98.02	4.92	0.72	98.45
Ostenol (**26**)	1.25	1.27	0.63	101.71	1.27	1.28	101.22

	5.00	5.04	0.85	100.84	5.04	0.85	100.84
Bisabolangelone (**27**)	12.50	12.54	0.07	100.35	12.55	0.16	100.40
	50.00	50.12	0.04	100.23	50.17	0.23	100.35
Byakangelicol (**28**)	2.50	2.54	0.57	101.43	2.52	1.21	100.96
	10.00	10.06	0.70	100.63	10.06	0.76	100.60
Oxypeucedanin (**29**)	12.50	12.72	0.48	101.73	12.65	1.17	101.21
	50.00	50.46	0.73	100.92	50.45	0.78	100.90
Columbianetin acetate (**30**)	12.50	12.61	0.02	100.91	12.61	0.02	100.91
	50.00	50.32	0.03	100.64	50.34	0.09	100.69
Coniferyl ferulate (**31**)	6.25	5.76	0.91	92.15	5.92	3.88	94.68
	25.00	22.48	0.90	89.91	23.35	5.73	93.38
Senkyunolide A (**32**)	3.75	3.66	0.29	97.53	3.66	0.50	97.70
	15.00	14.73	0.31	98.18	14.81	1.20	98.72
3-*n*-Butyl-phthalide (**33**)	1.25	1.26	1.23	100.73	1.25	0.70	100.14
	5.00	4.94	0.40	98.76	4.96	1.13	99.20
Imperatorin (**34**)	0.63	0.63	0.67	101.35	0.63	0.85	101.14
	2.50	2.45	0.13	98.07	2.46	0.65	98.42
Ligustilide (**35**)	37.50	37.71	0.04	100.55	37.70	0.02	100.54
	150.00	147.98	0.11	98.66	148.72	0.96	99.14
Phellopterin (**36**)	25.00	25.38	0.50	101.54	25.26	1.13	101.05
	100.00	100.90	0.79	100.90	100.86	0.85	100.86
Osthol (**37**)	31.25	31.56	0.04	101.00	31.56	0.06	100.98
	125.00	125.98	0.03	100.78	126.02	0.03	100.82
Decursin (**38**)	50.00	50.25	0.06	100.51	50.20	0.14	100.41
	200.00	196.41	0.01	98.21	197.38	0.85	98.69
Decursinol angelate (**39**)	50.00	50.26	0.06	100.53	50.21	0.15	100.43
	200.00	196.29	0.02	98.15	197.24	0.84	98.62

Isoimperatorin (**40**)	18.75	19.05	0.50	101.60	18.96	1.13	101.12
	75.00	75.67	0.81	100.90	75.68	0.79	100.91
Suberosin (**41**)	0.63	0.61	0.36	97.57	0.61	0.95	97.17
	2.50	2.47	0.15	98.61	2.48	0.75	99.11
Columbianadin (**42**)	12.50	12.60	0.00	100.83	12.60	0.02	100.82
	50.00	50.29	0.14	100.58	50.32	0.21	100.64
Falcarindiol (**43**)	18.75	18.85	0.32	100.54	18.85	0.32	100.54
	75.00	73.69	0.08	98.25	74.08	0.99	98.77
Praeruptorin B (**44**)	10.00	10.10	0.03	101.00	10.10	0.03	101.02
	40.00	39.85	0.04	99.64	39.90	0.19	99.74
Levistilide A (**45**)	12.50	12.56	0.44	100.50	12.59	0.43	100.68
	50.00	49.11	0.18	98.22	49.39	1.16	98.78
Praeruptorin C (**46**)	1.25	1.27	0.46	101.22	1.27	0.27	101.53
	5.00	5.03	0.07	100.54	5.03	0.13	100.65

Conc., concentration; RSD, relative standard deviation.

Table S5. Recoveries of the marker compounds (*n* = 3)

Compound	Initial conc. (μg/mL)	Spiked conc. (μg/mL)	Detected conc. (μg/mL)	Recovery (%)	RSD(%)
Chlorogenic acid (**1**)	32.17	37.50	73.32	109.72	1.57
		150.00	196.10	109.28	2.58
Caffeic acid (**2**)	0.52	1.00	1.45	92.72	1.40
		4.00	4.46	98.63	0.93
Prim-O-glucosyl-cimifugin (**3**)	20.19	6.25	27.06	109.93	8.22
		25.00	47.73	110.15	6.06
Cimifugin (**4**)	7.46	3.13	10.82	107.63	8.01
		12.50	21.35	111.13	3.34
Nodakenin (**5**)	147.18	20.00	168.91	108.68	5.51
		80.00	232.10	106.16	4.52
Umbelliferone (**6**)	9.33	4.40	13.70	99.34	4.15
		17.50	27.44	103.48	0.34
Ferulic acid (**7**)	6.93	5.63	13.10	109.77	1.51
		22.50	31.58	109.55	1.31
Benzoic acid (**8**)	0.47	0.63	1.14	107.86	1.14
		2.50	3.11	105.56	2.30
Senkyunolide I (**9**)	9.92	6.25	16.78	109.77	2.34
		25.00	37.16	108.96	1.07
Xanthotoxol (**10**)	0.79	0.63	1.45	105.59	2.47
		2.50	3.42	105.35	1.15
Senkyunolide H (**11**)	1.99	1.25	3.35	109.03	3.87
		5.00	7.40	108.30	0.75
Marmesin (**12**)	5.72	6.25	12.10	102.08	2.16
		25.00	31.47	103.02	0.92
sec-O-Glucosyl-hamaudol (**13**)	1.57	0.63	2.26	109.64	6.67
		2.50	4.34	110.69	1.31
Oxypeucedanin hydrate (**14**)	15.14	9.38	24.91	104.13	2.28
		37.50	55.56	107.79	1.05
Decursinol (**15**)	5.46	1.25	6.71	100.18	3.21
		5.00	10.55	101.73	1.71
Bergaptol (**16**)	3.68	3.13	6.95	104.48	3.55
		12.50	17.05	106.93	0.78
Byakangelicin (**17**)	13.69	12.50	26.90	105.73	1.85
		50.00	67.77	108.16	0.94
Psoralen (**18**)	0.61	0.63	1.25	102.10	1.51
		2.50	3.17	102.49	0.81
Angelol B (**19**)	39.38	6.25	45.63	99.86	5.71
		25.00	66.52	108.53	4.37
Angelol H (**20**)	14.66	3.13	17.62	94.88	7.23

		12.50	26.99	98.63	3.60
Angelicin (**21**)	0.60	0.63	1.29	110.25	0.94
		2.50	3.30	107.81	3.93
Xanthotoxin (**22**)	3.03	1.25	4.29	100.88	3.31
		5.00	8.14	102.14	1.39
Angelol A (**23**)	0.36	0.63	1.01	104.44	1.56
		2.50	3.04	107.38	0.80
Angelol G (**24**)	54.40	9.38	63.42	96.24	8.54
		37.50	93.71	104.84	5.87
Bergapten (**25**)	1.68	1.25	2.94	101.18	2.79
		5.00	6.80	102.35	0.98
Ostenol (**26**)	0.06	0.63	0.72	105.12	2.10
		2.50	2.74	107.32	0.91
Bisabolangelone (**27**)	6.21	6.25	12.98	108.33	1.22
		25.00	33.25	108.15	1.01
Byakangelicol (**28**)	2.02	1.25	3.30	102.36	3.01
		5.00	7.33	106.13	0.55
Oxypeucedanin (**29**)	10.14	6.25	16.70	104.86	3.31
		25.00	37.35	108.81	0.64
Columbianetin acetate (**30**)	10.43	6.25	17.17	107.87	1.42
		25.00	37.61	108.74	1.50
Coniferyl ferulate (**31**)	1.80	1.00	2.84	104.50	3.03
		4.00	6.12	107.94	2.16
Senkyunolide A (**32**)	3.76	3.75	7.68	104.45	1.63
		15.00	19.85	107.23	2.97
3-*n*-Butyl-phthalide (**33**)	0.93	1.25	2.19	100.80	4.77
		5.00	6.12	103.72	1.26
Imperatorin (**34**)	0.14	0.63	0.76	99.13	0.68
		2.50	2.70	102.35	0.48
Ligustilide (**35**)	67.32	37.50	108.48	109.75	2.64
		150.00	229.76	108.29	1.01
Phellopterin (**36**)	16.36	12.50	29.58	105.81	2.98
		50.00	70.72	108.72	0.59
Osthol (**37**)	80.79	15.63	96.75	102.13	1.62
		62.50	148.47	108.28	2.71
Decursin (**38**)	495.60	50.00	548.89	106.58	6.86
		200.00	701.51	102.95	6.29
Decursinol angelate (**39**)	471.55	50.00	523.77	104.43	4.61
		200.00	678.01	103.23	6.44
Isoimperatorin (**40**)	10.41	9.38	20.20	104.49	1.89
		37.50	50.91	108.00	0.69

Suberosin (**41**)	0.57	0.63	1.19	99.32	2.44
		2.50	3.18	104.39	2.55
Columbianadin (**42**)	38.81	6.25	45.13	101.13	3.23
		25.00	65.66	107.42	3.17
Falcarindiol (**43**)	50.42	18.75	69.77	103.20	4.15
		75.00	131.14	107.63	0.99
Praeruptorin B (**44**)	19.47	5.00	24.53	101.08	2.92
		20.00	41.51	110.17	4.26
Levistilide A (**45**)	12.87	12.50	26.55	109.49	1.15
		50.00	66.86	107.99	1.02
Praeruptorin C (**46**)	3.79	0.63	4.39	96.12	6.94
		2.50	6.55	110.34	2.29

Conc., concentration; RSD, relative standard deviation.

Table S6. Mean amounts of the marker compounds in the methanol extracts of *Angelica acutiloba* samples (mg/g)

Compound	AAC-01	AAC-02	AAC-03	AAC-04	AAC-05	AAC-06	AAC-07	AAC-08	AAC-09	AAC-10	AAC-11	AAC-12	AAC-13
Chlorogenic acid (**1**)	11.779 ± 0.382	4.795 ± 0.014	1.474 ± 0.046	2.892 ± 0.101	1.644 ± 0.059	4.170 ± 0.091	13.170 ± 0.321	3.284 ± 0.071	3.523 ± 0.044	6.531 ± 0.093	4.408 ± 0.188	5.059 ± 0.076	4.689 ± 0.241
Caffeic acid (**2**)	0.463 ± 0.016	0.074 ± 0.004	0.047 ± 0.003	0.057 ± 0.002	0.101 ± 0.005	0.220 ± 0.008	0.137 ± 0.009	0.099 ± 0.001	0.176 ± 0.007	0.087 ± 0.004	0.038 ± 0.003	0.059 ± 0.002	-
Nodakenin (**5**)	28.338 ± 0.535	-	-	-	-	2.257 ± 0.022	-	-	-	-	-	-	-
Umbelliferone (**6**)	-	1.068 ± 0.033	0.316 ± 0.010	-	3.379 ± 0.056	-	1.067 ± 0.016	0.821 ± 0.029	1.005 ± 0.022	1.613 ± 0.031	0.322 ± 0.007	0.567 ± 0.002	0.213 ± 0.004
Ferulic acid (**7**)	0.881 ± 0.022	-	0.127 ± 0.004	0.138 ± 0.002	-	-	0.399 ± 0.014	0.222 ± 0.007	0.250 ± 0.012	0.220 ± 0.006	0.112 ± 0.005	0.146 ± 0.003	0.057 ± 0.003
Benzoic acid (**8**)	-	-	-	-	0.044 ± 0.002	-	-	-	-	-	-	-	-
Senkyunolide I (**9**)	-	1.320 ± 0.016	0.852 ± 0.012	0.998 ± 0.024	0.900 ± 0.013	0.585 ± 0.007	0.922 ± 0.022	1.059 ± 0.020	0.529 ± 0.021	0.871 ± 0.014	0.468 ± 0.009	0.642 ± 0.008	0.287 ± 0.006
Xanthotoxol (**10**)	-	0.039 ± 0.001	-	-	0.030 ± 0.001	0.025 ± 0.001	0.040 ± 0.000	-	-	0.025 ± 0.000	0.028 ± 0.001	0.026 ± 0.001	-
Senkyunolide H (**11**)	-	0.191 ± 0.004	0.115 ± 0.002	0.146 ± 0.004	0.127 ± 0.003	0.088 ± 0.002	0.128 ± 0.002	0.147 ± 0.006	0.086 ± 0.002	0.138 ± 0.002	0.076 ± 0.002	0.092 ± 0.002	0.045 ± 0.001
Marmesin (**12**)	1.640 ± 0.036	-	-	-	-	0.219 ± 0.002	-	-	-	-	-	-	-
Decursinol (**15**)	1.760 ± 0.008	-	-	0.037 ± 0.000	-	0.334 ± 0.002	-	-	-	-	-	0.045 ± 0.002	-
Psoralen (**18**)	0.231 ± 0.005	0.358 ± 0.005	0.086 ± 0.001	0.801 ± 0.020	0.910 ± 0.013	0.223 ± 0.002	0.696 ± 0.009	0.234 ± 0.004	0.091 ± 0.001	0.285 ± 0.004	0.153 ± 0.003	0.268 ± 0.003	0.053 ± 0.001
Xanthotoxin (**22**)	2.292 ± 0.049	2.267 ± 0.029	0.467 ± 0.006	3.473 ± 0.085	4.683 ± 0.071	1.004 ± 0.011	5.612 ± 0.074	1.295 ± 0.025	1.066 ± 0.019	3.640 ± 0.054	1.037 ± 0.021	1.549 ± 0.017	0.416 ± 0.009
Bergapten (**25**)	0.412 ± 0.009	0.522 ± 0.004	0.141 ± 0.006	0.657 ± 0.008	0.619 ± 0.011	0.186 ± 0.005	0.885 ± 0.011	0.444 ± 0.002	0.199 ± 0.002	0.480 ± 0.005	0.214 ± 0.006	0.388 ± 0.006	0.115 ± 0.004
Coniferyl ferulate (**31**)	3.663 ± 0.099	-	-	-	-	0.564 ± 0.016	0.298 ± 0.007	-	-	-	-	0.382 ± 0.006	0.189 ± 0.005
Senkyunolide A (**32**)	-	0.343 ± 0.020	0.227 ± 0.006	0.305 ± 0.013	0.294 ± 0.004	-	1.554 ± 0.016	-	-	0.504 ± 0.006	0.246 ± 0.006	0.378 ± 0.004	0.225 ± 0.005
3-*n*-Butyl-phthalide (**33**)	-	0.419 ± 0.008	0.171 ± 0.003	0.295 ± 0.008	0.363 ± 0.006	0.163 ± 0.002	0.709 ± 0.010	0.165 ± 0.004	0.083 ± 0.001	0.408 ± 0.007	0.163 ± 0.005	0.236 ± 0.004	-
Imperatorin (**34**)	-	-	-	-	-	-	0.031 ± 0.000	0.011 ± 0.001	-	0.017 ± 0.001	0.018 ± 0.000	-	-

Ligustilide (**35**)	-	3.709 ± 0.045	2.320 ± 0.029	3.693 ± 0.090	3.302 ± 0.053	2.701 ± 0.029	5.030 ± 0.063	2.385 ± 0.047	5.735 ± 0.101	4.474 ± 0.068	4.407 ± 0.094	10.137 ± 0.121	6.647 ± 0.148
Decursin (**38**)	132.507 ± 1.693	0.310 ± 0.004	-	-	-	19.587 ± 0.236	-	-	-	-	-	-	-
Decursinol angelate (**39**)	127.857 ± 2.712	0.214 ± 0.003	-	-	-	22.420 ± 0.271	-	-	-	-	-	-	-
Isoimperatorin (**40**)	-	0.185 ± 0.003	0.188 ± 0.002	0.152 ± 0.004	-	-	0.177 ± 0.002	0.217 ± 0.004	0.111 ± 0.002	0.143 ± 0.002	0.073 ± 0.001	0.232 ± 0.002	--
Falcarindiol (**43**)	-	1.543 ± 0.012	0.632 ± 0.032	0.790 ± 0.034	0.699 ± 0.022	-	1.998 ± 0.034	1.048 ± 0.027	2.231 ± 0.034	2.623 ± 0.053	2.411 ± 0.050	1.910 ± 0.021	1.087 ± 0.031
Levistilide A (**45**)	-	0.419 ± 0.009	0.270 ± 0.006	0.290 ± 0.010	0.292 ± 0.006	0.148 ± 0.007	0.273 ± 0.015	0.384 ± 0.012	0.296 ± 0.008	0.281 ± 0.011	0.299 ± 0.010	0.331 ± 0.007	0.084 ± 0.003

AAC, *A.acutiloba*. The amounts of the marker compounds are expressed as 'Mean ± standard deviation'.

Table S7. Mean amounts of the marker compounds in the methanol extracts of *A. biserrata* samples (mg/g)

Compound	ABI-01	ABI -02	ABI -03	ABI-04	ABI-05	ABI-06	ABI-07	ABI-08	ABI-09	ABI-10
Chlorogenic acid (**1**)	1.763 ± 0.110	2.110 ± 0.058	1.800 ± 0.031	4.049 ± 0.121	0.703 ± 0.033	4.725 ± 0.135	2.284 ± 0.023	7.309 ± 0.202	10.277 ± 0.071	4.353 ± 0.144
Caffeic acid (**2**)	0.134 ± 0.009	0.056 ± 0.003	0.356 ± 0.016	0.162 ± 0.006	0.692 ± 0.029	0.260 ± 0.012	0.063 ± 0.004	0.214 ± 0.010	0.225 ± 0.012	0.190 ± 0.011
Umbelliferone (**6**)	-	-	2.654 ± 0.068	1.896 ± 0.061	12.763 ± 0.089	-	0.609 ± 0.021	-	0.452 ± 0.009	-
Ferulic acid (**7**)	1.312 ± 0.047	-	-	-	-	1.022 ± 0.028	-	0.202 ± 0.001	0.233 ± 0.002	0.080 ± 0.003
Senkyunolide I (**9**)	4.440 ± 0.143	0.813 ± 0.015	-	-	-	1.252 ± 0.027	-	-	-	-
Senkyunolide H (**11**)	0.798 ± 0.025	0.155 ± 0.003	-	-	-	0.238 ± 0.008	0.108 ± 0.002	-	-	-
Marmesin (**12**)	-	0.147 ± 0.003	0.281 ± 0.003	-	0.219 ± 0.004	0.437 ± 0.011	-	-	-	-
Oxypeucedanin hydrate (**14**)	-	0.107 ± 0.002	-	-	-	-	-	0.112 ± 0.005	-	-
Bergaptol (**16**)	-	-	-	-	-	-	1.951 ± 0.012	-	-	-
Byakangelicin (**17**)	-	0.049 ± 0.001	-	-	0.144 ± 0.001	-	-	0.050 ± 0.000	-	-
Psoralen (**18**)	-	0.031 ± 0.001	0.393 ± 0.003	0.302 ± 0.006	0.041 ± 0.001	0.812 ± 0.014	0.860 ± 0.009	-	-	-
Angelol B (**19**)	-	-	5.938 ± 0.048	7.756 ± 0.150	-	1.279 ± 0.026	0.120 ± 0.001	0.096 ± 0.001	0.209 ± 0.003	-
Angelol H (**20**)	-	-	2.321 ± 0.053	2.764 ± 0.054	-	0.388 ± 0.010	4.341 ± 0.116	1.461 ± 0.015	5.533 ± 0.063	1.154 ± 0.014
Angelicin (**21**)	-	0.109 ± 0.001	-	-	0.265 ± 0.002	-	1.527 ± 0.077	0.329 ± 0.006	1.755 ± 0.052	0.348 ± 0.013
Xanthotoxin (**22**)	-	0.030 ± 0.000	0.401 ± 0.006	1.156 ± 0.020	0.064 ± 0.001	2.250 ± 0.044	-	-	-	-
Angelol A (**23**)	-	-	34.523 ± 0.393	37.441 ± 0.671	-	8.977 ± 0.180	0.527 ± 0.006	0.361 ± 0.003	0.733 ± 0.010	0.059 ± 0.002
Angelol G (**24**)	-	-	8.995 ± 0.078	12.505 ± 0.228	-	19.749 ± 0.408	20.928 ± 0.207	6.367 ± 0.027	16.795 ± 0.172	6.777 ± 0.070
Bergapten (**25**)	-	0.103 ± 0.002	0.328 ± 0.004	0.649 ± 0.013	0.206 ± 0.002	0.811 ± 0.034	11.368 ± 0.146	18.844 ± 0.086	21.964 ± 0.239	12.405 ± 0.127

Ostenol (**26**)	-	0.448 ± 0.009	0.128 ± 0.001	0.521 ± 0.010	2.138 ± 0.016	0.229 ± 0.004	0.710 ± 0.006	0.521 ± 0.003	0.362 ± 0.004	0.055 ± 0.002
Bisabolangelone (**27**)	-	-	1.280 ± 0.014	2.419 ± 0.037	-	3.444 ± 0.068	0.247 ± 0.004	0.218 ± 0.001	0.439 ± 0.004	0.298 ± 0.003
Byakangelicol (**28**)	-	-	-	-	-	-	6.933 ± 0.058	1.847 ± 0.016	0.873 ± 0.006	1.310 ± 0.014
Columbianetin acetate (**30**)	-	-	1.800 ± 0.022	8.089 ± 0.144	-	2.176 ± 0.045	0.574 ± 0.004	-	-	-
Coniferyl ferulate (**31**)	0.915 ± 0.032	2.409 ± 0.011	-	-	-	0.954 ± 0.020	4.054 ± 0.034	7.133 ± 0.032	7.494 ± 0.087	7.478 ± 0.073
Imperatorin (**34**)	-	-	-	-	-	0.068 ± 0.002	-	0.897 ± 0.022	-	-
Ligustilide (**35**)	14.490 ± 0.456	10.532 ± 0.182	-	-	-	9.090 ± 0.176	5.639 ± 0.058	0.023 ± 0.000	0.050 ± 0.001	0.030 ± 0.001
Osthol (**37**)	-	-	13.162 ± 0.168	47.805 ± 0.840	-	9.791 ± 0.197	-	-	-	-
Decursin (**38**)	-	-	-	-	-	-	3.047 ± 0.036	-	-	-
Decursinol angelate (**39**)	-	-	-	-	-	-	0.466 ± 0.008	26.883 ± 0.118	22.876 ± 0.241	21.571 ± 0.205
Isoimperatorin (**40**)	-	-	0.656 ± 0.008	0.765 ± 0.014	-	0.369 ± 0.007	-	-	-	-
Suberosin (**41**)	-	3.014 ± 0.065	-	-	-	-	-	-	-	-
Columbianadin (**42**)	-	-	6.457 ± 0.077	15.515 ± 0.276	10.574 ± 0.086	7.637 ± 0.155	2.106 ± 0.031	1.342 ± 0.008	0.854 ± 0.001	0.547 ± 0.006
Falcarindiol (**43**)	11.317 ± 0.388	2.955 ± 0.174	-	-	-	7.475 ± 0.080	-	-	-	-
Praeruptorin B (**44**)	-	3.727 ± 0.071	-	-	5.690 ± 0.047	-	16.830 ± 0.169	3.619 ± 0.017	16.020 ± 0.171	2.996 ± 0.029
Levistilide A (**45**)	4.038 ± 0.136	2.040 ± 0.035	-	-	-	1.434 ± 0.054	2.206 ± 0.011	-	-	-
Praeruptorin C (**46**)	-	0.694 ± 0.009	-	-	-	-	-	-	-	-

ABI, ***A.*** *biserrata*. The amounts of the marker compounds are expressed as 'Mean ± standard deviation'.

Table S8. Mean amounts of the marker compounds in the methanol extracts of *A. dahurica* samples (mg/g)

Compound	ADA-01	ADA-02	ADA-03	ADA-04	ADA-05	ADA-06	ADA-07	ADA-08	ADA-09	ADA-10	ADA-11	ADA-12	ADA-13	ADA-14	ADA-15
Chlorogenic acid (**1**)	5.321 ± 0.191	0.824 ± 0.024	2.882 ± 0.094	3.780 ± 0.127	3.408 ± 0.035	3.501 ± 0.056	1.119 ± 0.019	4.975 ± 0.111	0.844 ± 0.012	3.580 ± 0.094	4.567 ± 0.138	12.251 ± 0.209	1.956 ± 0.060	2.620 ± 0.024	5.612 ± 0.142
Caffeic acid (**2**)	0.317 ± 0.008	0.093 ± 0.007	0.122 ± 0.002	0.175 ± 0.005	0.263 ± 0.005	0.763 ± 0.014	0.163 ± 0.004	0.174 ± 0.005	0.067 ± 0.002	0.115 ± 0.001	0.246 ± 0.005	0.196 ± 0.006	0.059 ± 0.002	0.156 ± 0.004	0.194 ± 0.007
Prim-O-glucosyl-cimifugin (**3**)	-	-	-	-	7.348 ± 0.046	-	4.871 ± 0.120	-	-	-	-	-	-	-	-
Ferulic acid (**7**)	0.210 ± 0.004	-	0.088 ± 0.003	0.333 ± 0.005	0.119 ± 0.003	0.486 ± 0.008	0.047 ± 0.002	0.298 ± 0.003	0.056 ± 0.003	0.292 ± 0.003	0.185 ± 0.008	0.382 ± 0.003	0.177 ± 0.004	0.194 ± 0.003	0.305 ± 0.003
Xanthotoxol (**10**)	0.069 ± 0.001	0.095 ± 0.001	0.099 ± 0.001	0.086 ± 0.001	1.206 ± 0.005	0.109 ± 0.001	1.196 ± 0.016	0.321 ± 0.003	0.310 ± 0.004	0.275 ± 0.007	0.135 ± 0.002	0.173 ± 0.002	0.190 ± 0.006	0.174 ± 0.002	0.345 ± 0.003
Marmesin (**12**)	-	0.037 ± 0.001	0.064 ± 0.004	0.069 ± 0.001	0.104 ± 0.001	0.151 ± 0.001	-	0.091 ± 0.003	0.099 ± 0.002	0.213 ± 0.006	0.157 ± 0.000	0.061 ± 0.001	0.077 ± 0.000	0.091 ± 0.002	0.269 ± 0.004
Oxypeucedanin hydrate (**14**)	2.621 ± 0.047	3.734 ± 0.068	4.231 ± 0.053	4.925 ± 0.047	8.477 ± 0.072	8.519 ± 0.082	5.930 ± 0.059	9.788 ± 0.200	6.628 ± 0.090	14.484 ± 0.376	7.710 ± 0.086	7.406 ± 0.077	8.077 ± 0.195	5.843 ± 0.054	10.514 ± 0.079
Byakangelicin (**17**)	3.148 ± 0.013	2.767 ± 0.049	3.582 ± 0.046	7.228 ± 0.127	3.446 ± 0.033	10.603 ± 0.088	2.597 ± 0.026	12.003 ± 0.043	6.229 ± 0.098	21.371 ± 0.523	8.988 ± 0.099	6.986 ± 0.070	9.565 ± 0.107	5.946 ± 0.059	15.621 ± 0.179
Psoralen (**18**)	0.223 ± 0.005	0.150 ± 0.003	0.354 ± 0.005	0.357 ± 0.006	0.075 ± 0.001	0.299 ± 0.005	-	0.111 ± 0.002	0.406 ± 0.006	2.008 ± 0.049	0.489 ± 0.006	0.804 ± 0.008	0.108 ± 0.005	0.125 ± 0.001	0.649 ± 0.032
Xanthotoxin (**22**)	0.104 ± 0.003	0.296 ± 0.006	0.322 ± 0.005	0.305 ± 0.004	1.296 ± 0.010	0.215 ± 0.002	0.032 ± 0.000	0.334 ± 0.003	0.461 ± 0.007	3.715 ± 0.091	0.495 ± 0.006	0.717 ± 0.008	0.104 ± 0.002	0.100 ± 0.001	0.643 ± 0.045
Angelol A (**23**)	0.106 ± 0.003	0.171 ± 0.005	0.213 ± 0.005	0.094 ± 0.002	0.286 ± 0.006	0.161 ± 0.002	0.257 ± 0.003	0.313 ± 0.006	0.178 ± 0.003	1.032 ± 0.024	0.166 ± 0.003	0.186 ± 0.003	0.151 ± 0.004	0.155 ± 0.003	0.429 ± 0.003
Bergapten (**25**)	0.109 ± 0.003	1.030 ± 0.021	1.417 ± 0.022	0.268 ± 0.003	1.949 ± 0.016	0.366 ± 0.003	0.802 ± 0.009	0.538 ± 0.008	1.283 ± 0.019	0.859 ± 0.017	0.318 ± 0.003	0.317 ± 0.003	0.346 ± 0.007	0.248 ± 0.002	0.409 ± 0.003

Ostenol (**26**)	0.056 ± 0.001	0.157 ± 0.005	0.131 ± 0.002	-	0.620 ± 0.006	-	0.708 ± 0.002	-	-	0.141 ± 0.001	-	-	-	-	-
Byakangelicol (**28**)	6.054 ± 0.184	2.568 ± 0.069	6.890 ± 0.136	18.701 ± 0.385	0.256 ± 0.002	14.560 ± 0.261	-	11.341 ± 0.059	0.956 ± 0.025	27.402 ± 0.846	5.383 ± 0.067	5.044 ± 0.109	12.510 ± 0.324	3.830 ± 0.058	13.913 ± 0.172
Oxypeucedanin (**29**)	10.410 ± 0.298	8.204 ± 0.206	17.018 ± 0.317	21.605 ± 0.408	1.241 ± 0.015	19.268 ± 0.309	1.457 ± 0.023	23.185 ± 0.128	4.192 ± 0.077	26.353 ± 0.773	5.389 ± 0.062	7.629 ± 0.106	17.734 ± 0.431	6.153 ± 0.085	11.651 ± 0.079
Imperatorin (**34**)	5.570 ± 0.137	11.129 ± 0.236	7.171 ± 0.112	5.667 ± 0.083	21.347 ± 0.186	15.830 ± 0.179	29.245 ± 0.330	11.613 ± 0.093	11.269 ± 0.178	8.968 ± 0.225	5.891 ± 0.069	4.100 ± 0.045	10.037 ± 0.222	8.647 ± 0.098	19.399 ± 0.187
Phellopterin (**36**)	2.643 ± 0.064	7.605 ± 0.160	6.280 ± 0.099	4.239 ± 0.061	20.727 ± 0.093	8.172 ± 0.092	31.988 ± 0.355	12.909 ± 0.111	7.879 ± 0.125	7.617 ± 0.191	3.849 ± 0.043	3.893 ± 0.042	9.108 ± 0.232	5.466 ± 0.055	11.286 ± 0.111
Decursin (**38**)	-	0.119 ± 0.002	-	-	-	-	-	-	-	-	-	-	-	-	-
Isoimperatorin (**40**)	5.194 ± 0.124	4.596 ± 0.093	4.839 ± 0.072	9.264 ± 0.132	14.940 ± 0.155	12.637 ± 0.129	17.641 ± 0.206	16.698 ± 0.145	5.017 ± 0.072	15.057 ± 0.372	6.370 ± 0.081	6.312 ± 0.062	11.631 ± 0.251	6.752 ± 0.071	13.421 ± 0.120
Suberosin (**41**)	-	0.521 ± 0.012	0.167 ± 0.006	-	0.542 ± 0.002	-	0.140 ± 0.003	0.268 ± 0.006	0.365 ± 0.009	-	0.189 ± 0.003	-	-	-	-
Falcarindiol (**43**)	1.023 ± 0.016	3.379 ± 0.089	5.151 ± 0.092	1.747 ± 0.006	15.614 ± 0.154	1.196 ± 0.022	9.696 ± 0.104	2.969 ± 0.046	5.031 ± 0.180	-	0.850 ± 0.037	1.391 ± 0.038	2.439 ± 0.074	1.811 ± 0.044	4.456 ± 0.112

ADA, *A.dahurica*. The amounts of the marker compounds are expressed as 'Mean ± standard deviation'.

Table S9. Mean amounts of the marker compounds in the methanol extracts of *A. decursiva* samples (mg/g)

Compound	ADE-01	ADE-02	ADE-03	ADE-04	ADE-05	ADE-06	ADE-07	ADE-08	ADE-09	ADE-10	ADE-11	ADE-12	ADE-13
Chlorogenic acid (**1**)	0.518 ± 0.012	0.192 ± 0.005	0.274 ± 0.008	0.266 ± 0.008	0.405 ± 0.024	1.280 ± 0.030	0.165 ± 0.008	0.723 ± 0.040	6.826 ± 0.233	6.944 ± 0.052	5.476 ± 0.142	6.003 ± 0.161	4.052 ± 0.178
Nodakenin (**5**)	3.483 ± 0.265	-	-	-	-	-	-	-	67.853 ± 0.461	56.985 ± 0.698	89.051 ± 0.905	63.972 ± 0.410	74.029 ± 2.153
Umbelliferone (**6**)	5.016 ± 0.106	3.971 ± 0.033	3.356 ± 0.064	4.677 ± 0.046	5.164 ± 0.041	4.244 ± 0.088	4.632 ± 0.066	4.354 ± 0.090	-	-	-	-	-
Marmesin (**12**)	0.930 ± 0.026	0.062 ± 0.001	0.064 ± 0.001	0.031 ± 0.000	0.045 ± 0.001	0.171 ± 0.004	0.063 ± 0.001	0.152 ± 0.006	0.557 ± 0.004	0.624 ± 0.007	0.539 ± 0.006	0.489 ± 0.001	0.369 ± 0.011
Decursinol (**15**)	0.083 ± 0.003	0.041 ± 0.000	0.030 ± 0.000	-	-	0.050 ± 0.001	0.042 ± 0.001	0.038 ± 0.003	0.092 ± 0.005	0.078 ± 0.003	0.111 ± 0.001	0.046 ± 0.001	0.058 ± 0.002
Psoralen (**18**)	0.180 ± 0.000	0.059 ± 0.005	0.081 ± 0.003	0.052 ± 0.001	0.051 ± 0.001	0.512 ± 0.014	0.039 ± 0.004	0.189 ± 0.004	-	-	-	-	-
Xanthotoxin (**22**)	0.643 ± 0.016	0.245 ± 0.002	0.192 ± 0.002	0.115 ± 0.001	0.209 ± 0.001	0.636 ± 0.024	0.308 ± 0.005	0.674 ± 0.014	0.022 ± 0.000	-	-	-	-
Bergapten (**25**)	0.240 ± 0.008	0.390 ± 0.003	0.351 ± 0.004	0.306 ± 0.003	0.313 ± 0.003	0.584 ± 0.011	0.361 ± 0.006	0.597 ± 0.012	0.030 ± 0.000	0.035 ± 0.000	-	-	-
Ostenol (**26**)	0.182 ± 0.020	0.109 ± 0.001	0.083 ± 0.001	0.104 ± 0.004	0.094 ± 0.003	0.272 ± 0.009	0.104 ± 0.001	0.116 ± 0.003	-	-	-	-	-
Bisabolangelone (**27**)	1.288 ± 0.018	0.867 ± 0.003	0.709 ± 0.007	0.023 ± 0.002	-	-	0.357 ± 0.006	-	-	-	-	-	-
Coniferyl ferulate (**31**)	2.471 ± 0.121	-	-	-	-	-	-	-	5.085 ± 0.125	2.873 ± 0.066	4.340 ± 0.045	3.703 ± 0.047	4.087 ± 0.057
Decursin (**38**)	0.842 ± 0.008	0.317 ± 0.002	0.280 ± 0.005	0.198 ± 0.003	0.208 ± 0.004	0.314 ± 0.007	0.316 ± 0.012	0.309 ± 0.007	3.586 ± 0.026	0.926 ± 0.012	2.991 ± 0.035	0.609 ± 0.002	2.229 ± 0.065
Decursinol angelate (**39**)	-	-	-	-	-	-	-	-	6.797 ± 0.058	4.250 ± 0.047	7.481 ± 0.085	3.514 ± 0.014	5.539 ± 0.167
Columbianadin (**42**)	-	-	-	-	-	-	-	-	2.708 ± 0.074	2.118 ± 0.102	4.333 ± 0.064	0.966 ± 0.004	1.708 ± 0.092
Falcarindiol (**43**)	5.886 ± 0.204	11.645 ± 0.042	13.255 ± 0.196	14.360 ± 0.157	14.219 ± 0.108	10.850 ± 0.206	9.389 ± 0.143	12.485 ± 0.259	-	-	1.189 ± 0.021	-	-

ADE, *A.decursiva*. The amounts of the marker compounds are expressed as 'Mean ± standard deviation'.

Table S10. Mean amounts of the marker compounds in the methanol extracts of *A. gigas* samples (mg/g)

Compound	AGI-01	AGI-02	AGI-03	AGI-04	AGI-05	AGI-06	AGI-07	AGI-08	AGI-09	AGI-10	AGI-11
Chlorogenic acid (**1**)	10.024 ± 0.307	9.205 ± 0.202	9.491 ± 0.349	12.128 ± 0.287	10.910 ± 0.031	5.122 ± 0.098	11.007 ± 0.203	10.269 ± 0.147	12.684 ± 0.089	13.471 ± 0.431	9.456 ± 0.247
Caffeic acid (**2**)	0.144 ± 0.008	0.279 ± 0.009	0.565 ± 0.024	0.103 ± 0.002	0.101 ± 0.003	0.077 ± 0.003	0.226 ± 0.003	0.296 ± 0.003	0.376 ± 0.009	0.196 ± 0.005	0.043 ± 0.001
Nodakenin (**5**)	15.025 ± 0.293	26.372 ± 0.345	23.284 ± 0.534	15.187 ± 0.231	17.472 ± 0.113	18.660 ± 0.323	19.279 ± 0.266	30.228 ± 0.379	21.818 ± 0.211	15.088 ±0.235	9.805 ± 0.089
Marmesin (**12**)	0.746 ± 0.019	1.504 ± 0.018	1.449 ± 0.036	0.737 ± 0.031	0.726 ± 0.006	0.398 ± 0.017	0.874 ± 0.013	2.211 ± 0.022	1.144 ± 0.009	0.566 ± 0.013	0.416 ± 0.007
Decursinol (**15**)	2.061 ± 0.022	1.536 ± 0.020	1.776 ± 0.018	2.706 ± 0.019	1.574 ± 0.030	3.651 ± 0.048	1.993 ± 0.025	2.870 ± 0.027	0.891 ± 0.004	0.924 ± 0.003	0.538 ± 0.008
Psoralen (**18**)	0.047 ± 0.001	0.065 ± 0.001	0.250 ± 0.006	0.332 ± 0.007	0.150 ± 0.001	0.041 ± 0.001	0.278 ± 0.003	2.623 ± 0.030	0.097 ± 0.001	0.032 ± 0.001	0.122 ± 0.002
Xanthotoxin (**22**)	0.048 ± 0.002	0.186 ± 0.003	0.795 ± 0.018	0.477 ± 0.010	0.602 ± 0.005	0.012 ± 0.000	0.804 ± 0.012	7.707 ± 0.087	0.555 ± 0.007	0.058 ± 0.001	0.437 ± 0.007
Bergapten (**25**)	0.100 ± 0.002	0.154 ± 0.002	0.336 ± 0.008	0.185 ± 0.003	0.147 ± 0.001	0.067 ± 0.001	0.213 ± 0.003	0.688 ± 0.006	0.279 ± 0.004	0.152 ± 0.005	0.189 ± 0.003
Coniferyl ferulate (**31**)	0.330 ± 0.014	1.618 ± 0.060	2.291 ± 0.078	0.445 ± 0.016	0.434 ± 0.009	0.698 ± 0.010	0.832 ± 0.023	2.371 ± 0.020	3.673 ± 0.047	0.847 ± 0.025	3.300 ± 0.105
Imperatorin (**34**)	0.044 ± 0.001	0.330 ± 0.004	0.064 ± 0.002	-	0.032 ± 0.001	0.069 ± 0.002	0.040 ± 0.000	0.258 ± 0.004	0.025 ± 0.000	0.044 ± 0.001	0.019 ± 0.001
Decursin (**38**)	88.211 ± 1.916	125.015 ± 1.189	120.429 ± 2.111	97.499 ± 1.953	88.387 ± 0.686	116.643 ± 1.575	107.347 ± 1.255	132.719 ± 0.946	92.042 ± 1.029	84.618 ± 1.739	93.120 ± 1.460
Decursinol angelate (**39**)	91.762 ± 2.151	108.000 ± 1.333	102.472 ± 2.426	107.230 ± 2.295	52.677 ± 0.462	192.851 ± 2.336	103.926 ± 1.431	102.543 ± 1.246	88.822 ± 1.092	70.031 ± 1.531	78.624 ± 1.327
Suberosin (**41**)	-	0.195 ± 0.013	-	-	-	-	-	-	-	-	0.247 ± 0.004

AGI, *Angelica gigas*. The amounts of the marker compounds are expressed as ‘Mean ± standard deviation’.

Table S11. Mean amounts of the marker compounds in the methanol extracts of *A. polymorpha* samples (mg/g)

Compound	APO-01	APO-02	APO-03	APO-04	APO-05	APO-06
Chlorogenic acid (**1**)	1.212 ± 0.041	2.970 ± 0.105	5.994 ± 0.291	3.316 ± 0.063	4.810 ± 0.153	0.462 ± 0.007
Ferulic acid (**7**)	1.296 ± 0.024	0.994 ± 0.014	1.204 ± 0.009	0.562 ± 0.032	2.108 ± 0.043	0.321 ± 0.012
Marmesin (**12**)	0.177 ± 0.006	0.036 ± 0.001	0.223 ± 0.003	0.035 ± 0.002	0.151 ± 0.001	0.189 ± 0.001
Oxypeucedanin hydrate (**14**)	1.338 ± 0.015	0.437 ± 0.008	2.518 ± 0.066	0.335 ± 0.003	3.118 ± 0.058	1.644 ± 0.008
Byakangelicin (**17**)	0.787 ± 0.020	0.164 ± 0.005	1.397 ± 0.025	0.141 ± 0.002	1.957 ± 0.053	0.459 ± 0.012
Bergapten (**25**)	0.614 ± 0.014	0.151 ± 0.004	1.769 ± 0.044	0.071 ± 0.000	5.311 ± 0.132	0.252 ± 0.002
Byakangelicol (**28**)	2.816 ± 0.059	0.751 ± 0.012	5.657 ± 0.146	0.746 ± 0.005	11.002 ± 0.219	1.932 ± 0.013
Oxypeucedanin (**29**)	9.966 ± 0.213	4.326 ± 0.066	24.752 ± 0.613	3.556 ± 0.019	40.843 ± 0.824	12.899 ± 0.085
Coniferyl ferulate (**31**)	8.533 ± 0.382	3.194 ± 0.118	4.135 ± 0.233	3.266 ± 0.151	6.177 ± 0.463	2.635 ± 0.109
Imperatorin (**34**)	0.058 ± 0.003	0.073 ± 0.001	1.401 ± 0.032	0.055 ± 0.000	0.240 ± 0.007	0.892 ± 0.005
Phellopterin (**36**)	0.541 ± 0.012	0.189 ± 0.002	1.336 ± 0.034	0.297 ± 0.002	1.340 ± 0.033	0.821 ± 0.007
Isoimperatorin (**40**)	2.997 ± 0.074	0.977 ± 0.012	7.907 ± 0.211	1.171 ± 0.005	5.341 ± 0.108	4.991 ± 0.026
Suberosin (**41**)	0.400 ± 0.008	0.052 ± 0.002	0.356 ± 0.011	0.037 ± 0.000	0.249 ± 0.004	0.253 ± 0.002
Falcarindiol (**43**)	30.960 ± 0.765	16.437 ± 0.220	29.615 ± 0.738	6.836 ± 0.042	54.795 ± 1.012	16.726 ± 0.072

APO, *A. polymorpha*. The amounts of the marker compounds are expressed as 'Mean ± standard deviation'.

Table S12. Mean amounts of the marker compounds in the methanol extracts of *A. reflexa* samples (mg/g)

Compound	ARE-01	ARE-02	ARE-03	ARE-04	ARE-05	ARE-06	ARE-07	ARE-08	ARE-09	ARE-10	ARE-11	ARE-12
Chlorogenic acid (**1**)	7.694 ± 0.369	8.596 ± 0.176	9.285 ± 0.119	7.997 ± 0.084	14.301 ± 0.427	4.589 ± 0.050	3.778 ± 0.032	8.762 ± 0.188	9.109 ± 0.143	8.507 ± 0.074	9.919 ± 0.180	5.664 ± 0.113
Caffeic acid (**2**)	0.381 ± 0.018	0.390 ± 0.013	0.358 ± 0.016	0.188 ± 0.006	0.188 ± 0.005	0.119 ± 0.002	0.297 ± 0.008	0.412 ± 0.009	0.357 ± 0.007	0.225 ± 0.001	0.190 ± 0.004	0.269 ± 0.004
Prim-O-glucosyl-cimifugin (**3**)	2.420 ± 0.092	2.880 ± 0.075	-	-	4.357 ± 0.059	-	-	2.530 ± 0.064	2.788 ± 0.061	-	-	4.952 ± 0.018
Cimifugin (**4**)	2.321 ± 0.093	1.379 ± 0.024	-	-	0.915 ± 0.031	-	-	2.258 ± 0.048	1.181 ± 0.015	-	-	2.111 ± 0.014
Ferulic acid (**7**)	0.678 ± 0.023	1.146 ± 0.011	2.593 ± 0.014	3.097 ± 0.058	0.968 ± 0.012	5.947 ± 0.034	2.390 ± 0.012	0.692 ± 0.010	0.441 ± 0.008	3.416 ± 0.024	3.156 ± 0.085	0.960 ± 0.009
Marmesin (**12**)	-	-	-	-	-	-	1.476 ± 0.015	-	-	-	-	-
sec-O-Glucosyl-hamaudol (**13**)	0.201 ± 0.012	0.254 ± 0.006	0.092 ± 0.002	0.106 ± 0.002	0.094 ± 0.001	0.070 ± 0.003	-	0.387 ± 0.010	0.652 ± 0.009	-	-	0.298 ± 0.003
Oxypeucedanin hydrate (**14**)	4.860 ± 0.207	6.903 ± 0.202	0.568 ± 0.007	0.592 ± 0.002	5.299 ± 0.081	0.465 ± 0.000	-	11.089 ± 0.230	4.323 ± 0.094	0.569 ± 0.007	0.796 ± 0.013	7.091 ± 0.048
Decursinol (**15**)	-	-	-	-	-	-	3.447 ± 0.029	-	-	-	-	-
Psoralen (**18**)	0.046 ± 0.003	0.151 ± 0.009	0.031 ± 0.001	0.018 ± 0.001	0.050 ± 0.002	0.060 ± 0.003	0.183 ± 0.003	0.102 ± 0.006	0.026 ± 0.001	0.063 ± 0.002	0.076 ± 0.005	-
Xanthotoxin (**22**)	0.058 ± 0.003	0.160 ± 0.006	0.082 ± 0.003	0.038 ± 0.001	0.061 ± 0.001	0.029 ± 0.001	0.353 ± 0.008	0.088 ± 0.010	0.118 ± 0.001	0.089 ± 0.002	0.104 ± 0.007	-
Bergapten (**25**)	0.437 ± 0.022	0.312 ± 0.010	0.131 ± 0.003	0.157 ± 0.000	0.256 ± 0.003	0.073 ± 0.004	0.206 ± 0.006	0.328 ± 0.003	0.133 ± 0.004	0.132 ± 0.001	0.182 ± 0.002	-
Ostenol (**26**)	2.186 ± 0.088	3.642 ± 0.056	-	-	3.896 ± 0.051	-	-	3.337 ± 0.060	1.410 ± 0.026	-	-	3.213 ± 0.026
Bisabolangelone (**27**)	9.145 ± 0.475	27.728 ± 0.653	9.004 ± 0.225	7.275 ± 0.115	3.976 ± 0.052	2.366 ± 0.040	0.276 ± 0.003	2.416 ± 0.054	0.079 ± 0.002	3.647 ± 0.053	9.039 ± 0.074	5.620 ± 0.048
Oxypeucedanin (**29**)	14.602 ± 0.639	27.543 ± 0.540	0.155 ± 0.002	0.135 ± 0.001	16.158 ± 0.231	0.089 ± 0.009	-	19.512 ± 0.393	2.381 ± 0.053	0.146 ± 0.002	1.159 ± 0.006	22.302 ± 0.141
Columbianetin acetate (**30**)	-	-	-	-	-	-	0.176 ± 0.003	-	-	-	-	-
Coniferyl ferulate (**31**)	1.425 ± 0.078	5.668 ± 0.197	1.795 ± 0.010	1.459 ± 0.023	-	-	-	-	-	0.804 ± 0.016	1.830 ± 0.032	1.088 ± 0.014
Imperatorin (**34**)	1.091 ± 0.048	6.636 ± 0.129	-	0.051 ± 0.000	0.876 ± 0.012	-	-	5.610 ± 0.109	1.562 ± 0.033	-	0.290 ± 0.001	6.429 ± 0.045

Ligustilide (**35**)	-	-	-	-	-	-	0.300 ± 0.005	-	-	-	-	-
Osthol (**37**)	5.839 ± 0.249	3.833 ± 0.077	-	-	5.290 ± 0.071	-	-	5.299 ± 0.101	8.372 ± 0.169	-	-	5.597 ± 0.036
Decursin (**38**)	-	3.111 ± 0.066	-	-	0.385 ± 0.005	-	68.772 ± 0.538	0.160 ± 0.006	-	-	1.462 ± 0.012	-
Decursinol angelate (**39**)	-	3.705 ± 0.072	-	-	0.347 ± 0.005	-	61.206 ± 0.481	0.178 ± 0.005	-	-	1.915 ± 0.011	-
Isoimperatorin (**40**)	8.041 ± 0.347	9.150 ± 0.194	0.309 ± 0.008	0.510 ± 0.005	7.319 ± 0.096	0.314 ± 0.001	-	7.536 ± 0.146	7.858 ± 0.162	0.284 ± 0.006	1.115 ± 0.011	10.956 ± 0.072
Falcarindiol (**43**)	-	1.949 ± 0.016	3.043 ± 0.032	3.608 ± 0.031	1.207 ± 0.016	10.695 ± 0.128	-	1.242 ± 0.069	-	7.224 ± 0.126	14.159 ± 0.128	2.009 ± 0.016

Compound	ARE-13	ARE-14	ARE-15	ARE-16	ARE-17	ARE-18	ARE-19	ARE-20	ARE-21	ARE-22	ARE-23	ARE-24
Chlorogenic acid (**1**)	13.492 ± 0.210	12.593 ± 0.244	16.356 ± 0.278	12.927 ± 0.100	11.994 ± 0.226	5.974 ± 0.227	7.073 ± 0.043	4.864 ± 0.039	6.395 ± 0.171	6.157 ± 0.050	12.764 ± 0.486	10.064 ± 0.299
Caffeic acid (**2**)	0.153 ± 0.002	0.203 ± 0.007	0.361 ± 0.005	0.238 ± 0.012	0.259 ± 0.006	0.174 ± 0.006	0.136 ± 0.002	0.265 ± 0.003	0.117 ± 0.001	0.247 ± 0.003	0.308 ± 0.008	0.500 ± 0.008
Prim-O-glucosyl-cimifugin (**3**)	3.778 ± 0.031	3.616 ± 0.053	6.535 ± 0.026	4.701 ± 0.063	2.787 ± 0.005	-	-	-	-	-	3.863 ± 0.095	4.719 ± 0.067
Cimifugin (**4**)	0.321 ± 0.024	1.830 ± 0.038	1.515 ± 0.010	1.012 ± 0.012	1.719 ± 0.009	-	-	-	-	-	1.405 ± 0.032	2.673 ± 0.023
Ferulic acid (**7**)	0.854 ± 0.003	0.658 ± 0.012	0.693 ± 0.005	0.667 ± 0.011	-	-	2.698 ± 0.033	4.607 ± 0.056	2.801 ± 0.054	3.866 ± 0.046	1.734 ± 0.033	0.606 ± 0.005
Marmesin (**12**)	-	0.297 ± 0.002	0.477 ± 0.001	0.254 ± 0.007	-	-	-	-	-	-	-	-
sec-O-Glucosyl-hamaudol (**13**)	0.067 ± 0.003	0.135 ± 0.002	0.320 ± 0.005	0.300 ± 0.003	0.366 ± 0.010	0.089 ± 0.001	0.081 ± 0.006	-	0.107 ± 0.010	0.090 ± 0.003	0.354 ± 0.002	0.534 ± 0.020
Oxypeucedanin hydrate (**14**)	6.499 ± 0.049	2.677 ± 0.048	8.861 ± 0.040	6.250 ± 0.061	6.502 ± 0.031	0.299 ± 0.005	0.385 ± 0.009	0.810 ± 0.009	0.474 ± 0.004	0.808 ± 0.005	8.544 ± 0.192	11.538 ± 0.123
Decursinol (**15**)	-	-	-	-	-	-	-	-	-	-	-	-
Psoralen (**18**)	0.031 ± 0.001	0.094 ± 0.001	0.186 ± 0.001	0.025 ± 0.001	0.057 ± 0.002	0.053 ± 0.003	0.293 ± 0.005	-	0.099 ± 0.003	0.072 ± 0.004	0.086 ± 0.002	0.061 ± 0.001
Xanthotoxin (**22**)	0.040 ± 0.000	0.132 ± 0.001	0.369 ± 0.008	0.031 ± 0.001	0.069 ± 0.000	0.099 ± 0.006	0.459 ± 0.005	0.126 ± 0.006	0.105 ± 0.003	0.064 ± 0.001	0.114 ± 0.002	0.034 ± 0.001
Bergapten (**25**)	0.222 ± 0.001	0.132 ± 0.001	1.758 ± 0.015	0.255 ± 0.004	0.254 ± 0.002	-	0.325 ± 0.002	0.247 ± 0.003	0.160 ± 0.006	0.100 ± 0.001	0.169 ± 0.004	0.225 ± 0.004
Ostenol (**26**)	4.453 ± 0.022	2.229 ± 0.022	3.908 ± 0.034	3.045 ± 0.016	2.313 ± 0.008	-	-	-	0.116 ± 0.006	-	3.802 ± 0.075	4.840 ± 0.034

Bisabolangelone (**27**)	11.435 ± 0.140	5.844 ± 0.053	3.694 ± 0.027	4.682 ± 0.058	2.793 ± 0.013	2.234 ± 0.023	3.134 ± 0.036	7.792 ± 0.103	5.911 ± 0.059	0.412 ± 0.007	0.696 ± 0.015	0.284 ± 0.006
Oxypeucedanin (**29**)	22.863 ± 0.240	9.134 ± 0.075	20.013 ± 0.130	14.746 ± 0.162	13.965 ± 0.055	-	0.104 ± 0.005	-	0.250 ± 0.001	-	7.825 ± 0.154	11.399 ± 0.095
Columbianetin acetate (**30**)	-	-	-	-	-	-	-	-	-	-	-	-
Coniferyl ferulate (**31**)	1.890 ± 0.037	1.137 ± 0.025	0.738 ± 0.041	1.726 ± 0.029	-	0.454 ± 0.019	1.356 ± 0.022	1.393 ± 0.021	1.612 ± 0.047	-	-	-
Imperatorin (**34**)	3.578 ± 0.039	0.894 ± 0.010	5.277 ± 0.036	2.376 ± 0.030	2.429 ± 0.024	-	0.038 ± 0.003	0.118 ± 0.001	-	0.127 ± 0.001	0.691 ± 0.036	1.740 ± 0.014
Ligustilide (**35**)	-	-	-	-	-	-	-	-	-	-	-	-
Osthol (**37**)	5.635 ± 0.061	4.978 ± 0.043	6.384 ± 0.035	6.929 ± 0.072	5.189 ± 0.009	-	-	-	-	-	8.220 ± 0.177	7.812 ± 0.057
Decursin (**38**)	-	15.900 ± 0.147	-	28.824 ± 0.344	0.163 ± 0.001	-	0.108 ± 0.001	-	-	0.284 ± 0.001	0.396 ± 0.009	-
Decursinol angelate (**39**)	-	16.549 ± 0.157	-	22.917 ± 0.281	0.158 ± 0.001	-	-	-	-	0.232 ± 0.002	0.419 ± 0.007	-
Isoimperatorin (**40**)	7.205 ± 0.086	3.275 ± 0.032	19.996 ± 0.130	3.546 ± 0.043	7.795 ± 0.028	0.322 ± 0.005	0.292 ± 0.004	0.874 ± 0.014	0.306 ± 0.004	0.559 ± 0.003	5.019 ± 0.114	6.554 ± 0.051
Falcarindiol (**43**)	1.027 ± 0.037	-	1.867 ± 0.018	1.426 ± 0.012	1.235 ± 0.022	12.221 ± 0.204	4.752 ± 0.016	15.942 ± 0.171	9.572 ± 0.100	6.193 ± 0.052	2.312 ± 0.038	0.660 ± 0.021

ARE, *A.reflexa*. The amounts of the marker compounds are expressed as 'Mean ± standard deviation'.

Table S13. Mean amounts of the marker compounds in the methanol extracts of *A. sinensis* samples (mg/g)

Compound	ASI-01	ASI-02	ASI-03	ASI-04	ASI-05	ASI-06	ASI-07	ASI-08	ASI-09	ASI-10	ASI-11	ASI-12
Chlorogenic acid (**1**)	0.677 ± 0.033	0.371 ± 0.022	4.514 ± 0.182	0.337 ± 0.024	0.595 ± 0.053	0.641 ± 0.024	3.385 ± 0.099	0.338 ± 0.010	0.251 ± 0.005	1.214 ± 0.054	0.416 ± 0.017	0.376 ± 0.012
Caffeic acid (**2**)	0.047 ± 0.005	0.031 ± 0.004	0.114 ± 0.015	0.024 ± 0.004	0.030 ± 0.004	0.044 ± 0.002	0.088 ± 0.004	0.042 ± 0.002	0.023 ± 0.002	0.071 ± 0.003	0.048 ± 0.003	0.024 ± 0.002
Ferulic acid (**7**)	2.209 ± 0.039	1.388 ± 0.011	1.163 ± 0.006	1.854 ± 0.019	1.978 ± 0.026	2.198 ± 0.051	0.747 ± 0.004	1.576 ± 0.013	1.853 ± 0.005	2.133 ± 0.046	2.055 ± 0.036	2.216 ± 0.024
Benzoic acid (**8**)	0.068 ± 0.003	0.043 ± 0.003	0.056 ± 0.007	0.049 ± 0.002	0.070 ± 0.006	0.064 ± 0.001	0.041 ± 0.003	0.048 ± 0.003	0.056 ± 0.001	0.093 ± 0.006	0.073 ± 0.003	0.045 ± 0.002
Senkyunolide I (**9**)	2.296 ± 0.070	1.699 ± 0.032	1.808 ± 0.033	2.526 ± 0.023	2.972 ± 0.046	3.088 ± 0.084	1.455 ± 0.013	1.411 ± 0.026	3.237 ± 0.004	3.168 ± 0.094	4.131 ± 0.069	3.114 ± 0.046
Senkyunolide H (**11**)	0.416 ± 0.016	0.304 ± 0.007	0.357 ± 0.008	0.477 ± 0.006	0.530 ± 0.008	0.546 ± 0.016	0.263 ± 0.003	0.274 ± 0.006	0.592 ± 0.007	0.577 ± 0.019	0.742 ± 0.014	0.568 ± 0.009
Senkyunolide A (**32**)	0.404 ± 0.014	0.423 ± 0.029	0.574 ± 0.005	0.597 ± 0.008	0.464 ± 0.009	0.369 ± 0.020	0.442 ± 0.005	0.694 ± 0.014	0.416 ± 0.011	0.974 ± 0.028	0.586 ± 0.033	0.532 ± 0.012
3-*n*-Butyl-phthalide (**33**)	0.435 ± 0.005	0.520 ± 0.004	0.150 ± 0.003	0.547 ± 0.007	0.363 ± 0.005	0.506 ± 0.009	0.164 ± 0.004	0.142 ± 0.001	0.508 ± 0.004	0.750 ± 0.015	0.779 ± 0.012	0.474 ± 0.004
Ligustilide (**35**)	30.033 ± 0.855	11.175 ± 0.207	8.616 ± 0.155	20.379 ± 0.191	32.023 ± 0.447	27.501 ± 0.715	6.402 ± 0.029	46.474 ± 0.834	22.380 ± 0.059	36.554 ± 1.038	18.361 ± 0.307	23.464 ± 0.331
Falcarindiol (**43**)	2.044 ± 0.088	0.723 ± 0.049	8.880 ± 0.128	1.490 ± 0.037	1.546 ± 0.016	1.987 ± 0.027	5.843 ± 0.191	2.627 ± 0.034	1.377 ± 0.027	1.624 ± 0.056	1.306 ± 0.028	1.588 ± 0.031
Levistilide A (**45**)	5.286 ± 0.178	1.866 ± 0.030	2.499 ± 0.047	1.701 ± 0.013	3.367 ± 0.060	5.295 ± 0.156	1.479 ± 0.020	1.971 ± 0.034	2.825 ± 0.015	2.693 ± 0.075	2.220 ± 0.037	2.266 ± 0.026

ASI, *A. sinensis*. The amounts of the marker compounds are expressed as 'Mean ± standard deviation'.

Table S14. Mean amounts of the marker compounds in the methanol extracts of *Conioselinum tenuissimum* samples (mg/g)

Compound	CTE-01	CTE-02	CTE-03	CTE-04	CTE-05	CTE-06	CTE-07	CTE-08
Chlorogenic acid (**1**)	1.128 ± 0.019	2.125 ± 0.039	3.062 ± 0.070	2.533 ± 0.045	2.775 ± 0.020	3.723 ± 0.077	2.488 ± 0.016	1.053 ± 0.008
Caffeic acid (**2**)	0.035 ± 0.001	0.053 ± 0.001	0.071 ± 0.001	0.049 ± 0.001	0.065 ± 0.002	0.036 ± 0.000	0.048 ± 0.000	-
Ferulic acid (**7**)	1.028 ± 0.005	0.847 ± 0.009	1.184 ± 0.015	1.090 ± 0.015	0.926 ± 0.006	1.236 ± 0.010	0.574 ± 0.006	0.620 ± 0.015
Benzoic acid (**8**)	0.069 ± 0.002	0.225 ± 0.004	0.056 ± 0.003	0.049 ± 0.002	0.038 ± 0.001	0.032 ± 0.002	0.125 ± 0.002	-
Senkyunolide I (**9**)	2.434 ± 0.019	3.834 ± 0.051	3.196 ± 0.043	2.334 ± 0.034	2.104 ± 0.017	2.324 ± 0.027	3.902 ± 0.033	0.894 ± 0.003
Senkyunolide H (**11**)	0.419 ± 0.004	0.536 ± 0.010	0.545 ± 0.008	0.384 ± 0.009	0.331 ± 0.003	0.377 ± 0.005	0.614 ± 0.013	0.155 ± 0.001
Bergapten (**25**)	-	-	-	-	-	-	-	-
Bisabolangelone (**27**)	0.086 ± 0.001	-	-	-	-	-	-	-
Coniferyl ferulate (**31**)	3.272 ± 0.046	0.248 ± 0.008	4.456 ± 0.095	4.966 ± 0.093	4.579 ± 0.074	9.532 ± 0.188	0.925 ± 0.009	8.968 ± 0.197
Senkyunolide A (**32**)	1.272 ± 0.014	-	2.118 ± 0.030	1.504 ± 0.023	1.110 ± 0.010	9.612 ± 0.113	3.285 ± 0.027	2.328 ± 0.016
3-*n*-Butyl-phthalide (**33**)	0.855 ± 0.007	1.292 ± 0.017	0.576 ± 0.006	0.496 ± 0.007	0.357 ± 0.002	1.140 ± 0.016	1.497 ± 0.009	0.294 ± 0.003
Ligustilide (**35**)	37.908 ± 0.270	11.013 ± 0.151	76.923 ± 1.056	63.354 ± 0.892	61.766 ± 0.482	58.015 ± 0.644	21.749 ± 0.174	110.078 ± 0.628
Falcarindiol (**43**)	0.603 ± 0.012	-	1.285 ± 0.037	1.355 ± 0.024	1.415 ± 0.016	1.440 ± 0.050	-	2.708 ± 0.048
Levistilide A (**45**)	3.853 ± 0.042	8.900 ± 0.116	6.863 ± 0.103	6.712 ± 0.118	5.775 ± 0.059	4.811 ± 0.068	6.918 ± 0.064	3.228 ± 0.028

CTE, *Conioselinum tenuissimum*. The amounts of the marker compounds are expressed as 'Mean ± standard deviation'.

Table S15. Mean amounts of the marker compounds in the methanol extracts of *Ostericum grosserratum* samples (mg/g)

Compound	OGR-01	OGR-02	OGR-03	OGR-04	OGR-05	OGR-06	OGR-07	OGR-08
Chlorogenic acid (**1**)	-	0.270 ± 0.001	0.400 ± 0.004	0.280 ± 0.012	0.634 ± 0.012	0.264 ± 0.002	0.327 ± 0.011	0.383 ± 0.010
Ferulic acid (**7**)	-	0.142 ± 0.002	0.257 ± 0.000	0.065 ± 0.001	0.230 ± 0.009	0.195 ± 0.001	-	0.173 ± 0.006
Psoralen (**18**)	0.049 ± 0.000	-	-	-	-	-	-	-
Xanthotoxin (**22**)	0.041 ± 0.000	-	-	-	-	-	-	-
Falcarindiol (**43**)	253.520 ± 4.443	304.869 ± 1.630	277.503 ± 1.905	355.783 ± 7.968	305.420 ± 4.119	432.044 ± 3.455	370.395 ± 11.298	209.759 ± 6.517

OGR, *Ostericum grosserratum*. The amounts of the marker compounds are expressed as ‘Mean ± standard deviation
